# Training and assessment of skills in neuraxial space access: a scoping review of educational approaches to lumbar puncture, epidural anaesthesia, and spinal anaesthesia

**DOI:** 10.1016/j.bja.2025.06.008

**Published:** 2025-07-07

**Authors:** Martine Siw Nielsen, Frederik Veitland Ilkjær, Anders Morten Grejs, Anders Bo Nielsen, Lars Konge, Anne Craveiro Brøchner

**Affiliations:** 1 Department of Anaesthesiology and Intensive Care, Lillebaelt University Hospital, Kolding, Denmark; 2 Research Unit of Medical Education, Department of Clinical Research, University of Southern Denmark, Odense, Denmark; 3 Department of Regional Health Research, Region of Southern Denmark, Odense, Denmark; 4 Department of Intensive Care Medicine, Aarhus University Hospital, Aarhus, Denmark; 5 Department of Clinical Medicine, Aarhus University, Aarhus, Denmark; 6 Department of Anaesthesiology and Intensive Care, Odense University Hospital, Svendborg, Denmark; 7 Copenhagen Academy for Medical Education and Simulation, Center for HR & Education, The Capital Region of Denmark, Copenhagen, Denmark; 8 Department of Clinical Medicine, University of Copenhagen, Copenhagen, Denmark

**Keywords:** assessment, education, epidural anaesthesia, lumbar puncture, neuraxial access, spinal anaesthesia, training

## Abstract

**Background:**

Neuraxial space access, including lumbar puncture, spinal anaesthesia, and epidural anaesthesia, is important in clinical practise for diagnostics and anaesthesia. Despite frequent use, standardised educational recommendations for training and assessing proficiency in these procedures are not well-integrated. The following research question was formulated: what is known from published literature to guide future educational recommendations for training and skills assessment of neuraxial space access with and without the use of ultrasound?

**Methods:**

On May 7, 2024, searches were performed in the Cochrane Library, MEDLINE (Ovid), Scopus, PubMed, CINAHL (EBSCO), and EMBASE (Ovid). Studies were eligible if they involved physicians, medical students, or nurses and focused on training or assessment of neuraxial space access skills. No comparator was required, and all study designs were included if outcomes could be assessed using Kirkpatrick framework. There were no restrictions on language or publication date. Methodological quality was assessed using the Medical Education Research Study Quality Instrument (MERSQI).

**Results:**

The review included 99 studies, and overall, 28 (28%) of the included studies were of low quality with a MERSQI score <9, whereas 22 (22%) were of high quality with a MERSQI score of ≥14. The designs were primarily cohort studies (68%) and RCTs (24%). Specialities represented were mainly anaesthesia (22%) and paediatrics (17%), but many were not reported (30%). Training modalities varied, predominantly using low-fidelity manikins (55%). Simulation-based methods enhanced procedural confidence and technical skills.

**Conclusions:**

Studies on education in neuraxial space access show substantial variation in educational approaches and assessment. Consistent findings indicate that simulation-based training enhances outcomes across multiple Kirkpatrick levels, although the studies generally have low methodological quality. Further high-quality research is needed, especially linking training and assessment to patient outcomes.

**Editor’s key points**

• Accessing the neuraxial space is a common procedure in medical practise, but a lack of standardisation in how to structure training and competency assessment makes it challenging to implement best practices.
• This scoping review article consolidates findings from 99 studies and synthesises various training approaches, assessment methods, and training outcomes.
• The findings highlight the importance of proficiency-based training for procedural skills. Simulation-based interventions are effective for building competency. Attention should be given to developing and gathering validity evidence for assessment tools to objectively ensure competency.

Access to the neuraxial space is a frequently used technique for performing lumbar punctures and neuraxial anaesthesia. These procedures share similarities in their procedural approach with the use of a needle to access either the spinal canal or the epidural space. The success of establishing neuraxial space access is highly dependent on the operator’s proficiency, as numerous studies have demonstrated that physicians with limited experience require more attempts and are less successful in achieving neuraxial space access compared with more trained colleagues.^1^, ^2^, ^3^ Training approaches and assessments vary but generally follow a procedure-based model with a fixed number of procedures required, a time-based model with a set training duration, or a proficiency-based model, particularly in the form of mastery learning, where training continues until each individual trainee meets the predefined performance standards. Simulation-based training plays an important role across these approaches. This includes the use of low-fidelity manikins that provide basic anatomical representations. It also involves high-fidelity manikins, which incorporate advanced technologies and feedback to simulate more complex scenarios.

The American Society of Anesthesiologists (ASA) offers detailed best practise guidelines for the use of neuraxial anaesthesia in the care of the obstetric patient, whereas the British Pain Society provides guidelines for performing neuraxial anaesthesia more generally.^4^^,^^5^ However, they do not provide specific recommendations on the training or skill acquisition needed to perform these procedures. Likewise, multiple consensus-based best practise guidelines comprehensively outline the indications, techniques, and potential challenges associated with lumbar puncture, but lack educational recommendations to ensure proficiency in the procedure.^6^^,^^7^

Similarly, to other procedures, the education of procedures establishing neuraxial space access should rely on the best available evidence from learning and clinical studies.^8^^,^^9^

To support this, the current scoping review was conducted to map the research published regarding the training and assessment of neuraxial space access with and without ultrasound and to identify existing knowledge gaps. The following research question was formulated: what is known from published literature to guide future educational recommendations in training and skills assessment of neuraxial space access with and without the use of ultrasound?

## Methods

### Protocol and registration

The protocol for this scoping review was drafted according to the Preferred Reporting Items for Systematic Review and Meta-Analysis Protocol (PRISMA-P) and the PRISMA Extension for Scoping Reviews (PRISMA-ScR) guidelines, and it was later published in an international peer-reviewed journal.^10^, ^11^, ^12^

### Eligibility criteria

To be considered in the review, published research needed to include a population of physicians, medical students, or nurses. Studies including other healthcare professions alongside the desired population were also considered, given that data specific to our target population could be extracted. Furthermore, the intervention should include education, training, or assessment in procedural neuraxial space access skills with or without the use of ultrasound. No comparator was required, but it could be a different type of education, training, or assessment in the procedure. Lastly, a criterion for inclusion was that the outcome should be measurable on at least one of Kirkpatrick's four levels of training evaluation: level 1, reaction (learners' reactions and satisfaction with the training programme); level 2, learning (competence demonstrated in an educational setting); level 3, impact (competence applied in the clinical setting, assessing skill transfer); or level 4, results (clinical outcomes influenced).^13^

All study designs were eligible for inclusion, including both quantitative and qualitative designs. Abstracts were likewise included if they adhered to the abovementioned criteria. Studies in all languages were accepted because of the availability of web-based translation tools. No restrictions were set on the publication date. Case reports, author responses, and commentaries were excluded.

### Information sources

To identify potentially relevant literature, the following databases were searched, and the references imported on May 7, 2024: Cochrane Library, MEDLINE (Ovid), Scopus, PubMed, CINAHL (EBSCO), and EMBASE (Ovid). The search strategy was developed based on the inclusion criteria and in collaboration with a research librarian at the University of Southern Denmark. The final search strategy for each database can be found in Supplementary Appendix 1. After the literature search, all identified studies were uploaded to EndNote20® (Alfasoft AB, Gothenburg, Sweden) software for duplicate removal. Subsequently, studies were uploaded to Covidence® (Veritas Health Innovation, Melbourne, VIC, Australia).

### Selection of sources of evidence

After duplicates were removed, two authors (MSN and FVI) independently reviewed the titles and abstracts from the search results, assessing their eligibility based on the inclusion criteria. Relevant manuscripts were then read in full text for potential inclusion. In cases of disagreement, a third author (ACB) was consulted to help resolve any discrepancies. Lastly, citations and references of articles included for data extraction were searched for additional relevant articles.

### Data charting process

Data from eligible studies were extracted using a standardised data extraction tool designed for this study. The data extraction template was developed and reviewed by all the authors before the beginning of the data extraction process. Data were independently extracted using a template in Covidence, from text, tables, or graphs by two authors (MSN and FVI), with any conflicts resolved by discussion with a third author (ACB).

### Data items

We extracted data on article characteristics (authors, publication year, country, conflicts of interest, study design), participant information (profession, medical speciality, number of participants enrolled, and number of participants completed), intervention characteristics (setting, skill trained/assessed, description of the intervention, duration of intervention, and hands-on training modality), outcome characteristics (outcomes, outcome measurement tools, and Kirkpatrick level for outcomes), and study conclusion.

### Critical appraisal of individual sources of evidence

The quality of the included educational studies was assessed using the Medical Education Research Study Quality Instrument (MERSQI) by two reviewers (MSN and FVI) independently, with discrepancies resolved during joint article review and discussion.

The MERSQI tool was created and evaluated to assess the methodological quality of education research studies.^14^ Unlike broader frameworks such as GRADE, which assess evidence quality across various fields, MERSQI specifically targets the key methodological elements relevant to education research, providing a detailed and focused approach to quality assessment. A final score was assigned from seven domains (with the possible score ranging 5–18), according to study design, number of institutions studied, response rate, type of data (self-assessment or objective measurements), validity of evaluation instruments, data analysis, and outcomes.

Because of the significant heterogeneity of study designs and interventions, a meta-analysis was not feasible.

### Synthesis of results

We grouped the studies by study characteristics (country, study design, population, and medical speciality), educational characteristics (setting, skills trained or assessed, and hands-on modality) along with outcomes (Kirkpatrick level), and study quality (MERSQI).

## Results

### Identified studies

**Fig 1:**
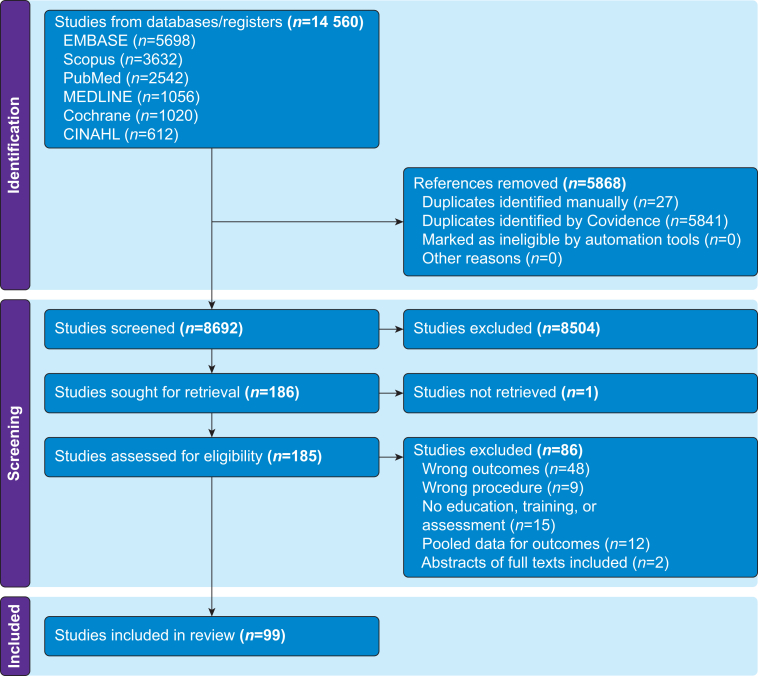
PRISMA flow chart.

An initial database search identified 14 560 studies, and after duplicate removal, 8692 studies were subjected to title and abstract screening. Title and abstract screening resulted in 186 studies for full text review. One study was not retrieved despite help from a librarian and e-mails to the corresponding author. Ultimately, 99 studies met the eligibility criteria and were included in the review (Fig. 1; Supplementary Appendix 2). No new studies were discovered during the review of citations and references of the included manuscripts.

### Study characteristics

In total, 22 countries were represented (Supplementary Appendix 3). Studies were mainly published within the past 10 years (61%), and most studies were conducted in the USA (55%) or Canada (10%). The primary study design was a cohort study (68%), followed by an RCT (24%) (Supplementary Appendix 3). The included trained population comprised medical doctors (73%), medical students (22%), nurses (1%), and a combination of the three (4%). Specialities represented were diverse, primarily anaesthesia (22%), paediatrics (17%), and internal medicine (11%), although many were not reported (30%) (Supplementary Appendix 3).

### Setting, skills trained, and training modalities

Training interventions were conducted in either a simulation centre (25%) or a clinical department (24%); however, dominantly, the setting was not described (42%) (Supplementary Appendix 4). The 99 studies covered adult lumbar puncture (55%), paediatric or infant lumbar puncture (22%), neuraxial anaesthesia (22%), and one study (1%) included both lumbar puncture and spinal anaesthesia (Supplementary Appendix 4). In 93 of the studies, the training intervention was based on a single hands-on modality, whereas six studies included more than one. The hands-on modalities included low-fidelity manikins (*n*=54) or not elaborated ‘simulation’ (*n*=18), live models (*n*=13) alongside mixed reality (*n*=4), cadavers (*n*=3), fruits (*n*=3), high-fidelity models (*n*=2), and virtual-reality simulators (*n*=1). Six studies did not elaborate on the training modality (Supplementary Appendix 4).

### Educational methods and effectiveness of training

#### Training in adult lumbar puncture

Training in lumbar puncture typically utilised simulation with manikins or cadavers, which effectively enhanced trainees’ confidence and skill levels.^15^, ^16^, ^17^, ^18^, ^19^, ^20^, ^21^, ^22^, ^23^, ^24^, ^25^, ^26^, ^27^, ^28^, ^29^, ^30^, ^31^, ^32^, ^33^, ^34^, ^35^, ^36^, ^37^, ^38^, ^39^, ^40^, ^41^, ^42^, ^43^, ^44^, ^45^, ^46^, ^47^, ^48^, ^49^, ^50^, ^51^, ^52^, ^53^, ^54^, ^55^, ^56^ Likewise, it improved trainees’ autonomy in clinical procedures and contributed to increased clinical success rates during patient procedures compared with pre-interventions and controls not receiving simulation-based training.^57^, ^58^, ^59^, ^60^, ^61^ Multiple studies incorporated lumbar puncture into a multiprocedural curriculum, which continued to demonstrate a positive impact on confidence and technical skills specific to lumbar puncture.^15^^,^^16^^,^^21^^,^^22^^,^^24^^,^^28^, ^29^, ^30^^,^^33^, ^34^, ^35^^,^^38^^,^^44^^,^^47^^,^^50^^,^^61^

Before a hands-on session, most programmes mandated theoretical instruction, delivered via instructor-led presentations, lectures, video resources, reading materials, or online modules.^15^^,^^18^^,^^22^^,^^23^^,^^29^^,^^31^^,^^33^^,^^35^, ^36^, ^37^^,^^39^^,^^41^^,^^45^^,^^46^^,^^48^, ^49^, ^50^, ^51^, ^52^, ^53^^,^^56^^,^^59^, ^60^, ^61^, ^62^, ^63^ One RCT highlighted that theoretical content focusing on the ‘how-and-why' was particularly beneficial for skill transfer compared with only knowing ‘how’ based on transferring learning to new problems.^48^ In six of the included studies, training designed as proficiency-based (training based on demonstrating the required level of skills) seemed to be beneficial for skills and superior to traditional training.^15^^,^^36^^,^^41^^,^^45^^,^^52^^,^^61^ Hands-on sessions were generally led by experienced instructors.^15^^,^^20^^,^^21^^,^^24^^,^^29^^,^^32^^,^^33^^,^^35^, ^36^, ^37^^,^^39^^,^^41^^,^^46^^,^^47^^,^^50^^,^^52^^,^^59^ Two studies concluded that peer training was equal to senior instructor-led training based on skills achievement.^19^^,^^27^ Additionally, one study reported that self-directed training resulted in longer skill retention compared with instructor-led approaches.^63^ Deliberate practise, defined as goal-oriented training with immediate feedback, was useful for outcomes based on Kirkpatrick levels 1, 2, and 4.^15^^,^^20^^,^^28^^,^^29^^,^^32^^,^^37^^,^^41^^,^^43^^,^^45^^,^^50^^,^^52^^,^^56^ Retention of skills after a simulation-based training programme was examined in multiple studies, where a considerable variation in overall retention was seen.^37^^,^^45^^,^^52^^,^^62^ One study, which followed participants for the longest period of 7 months, found that retention of lumbar puncture skills declined, with only 40% passing the minimum standard, down from 100% immediately after training.^52^

#### Training in infant/paediatric lumbar puncture

Simulation-based training in infant and paediatric lumbar puncture improved learners' procedural confidence and skills in six of the included studies.^64^, ^65^, ^66^, ^67^, ^68^, ^69^ Proficiency-based approaches, including mastery learning, promoted desirable clinical behaviours, were predictive of clinical success, and helped trainees achieve skill levels comparable with or exceeding those of senior colleagues in three studies.^70^, ^71^, ^72^ Some studies found that a single mastery learning session alone did not significantly improve procedural success and that an approach emphasising ‘invent and problem-solve, followed by instruction’ was equally effective to mastery-based learning.^40^^,^^73^ Just-in-time training, refresher training delivered immediately before a clinical procedure, was feasible and clinically valuable.^71^^,^^74^^,^^75^ Furthermore, incorporating simulation-based training into daily clinical shifts with senior instructors present led to increased procedural engagement, an outcome not achieved by simply providing training videos and simulation equipment without mandatory senior supervision.^76^^,^^77^

#### Training in epidural and spinal anaesthesia

In four studies on neuraxial anaesthesia training, proficiency-based training was found to be feasible within clinical departments, enhancing procedural confidence and resulting in better patient-related outcomes compared with procedural-based approaches.^78^, ^79^, ^80^, ^81^ Low-fidelity task-trainer education proved equal to high-fidelity simulation-based training, in terms of epidural catheter placement on patients, and a one-day simulation-based course early in anaesthesia speciality training increased the number of performed epidural catheter placements on patients compared with historical cohorts.^82^^,^^83^ Additionally, anaesthesiologists who completed an extra simulation-based training course, alongside standard clinical training, demonstrated improvements in spinal anaesthesia performance as assessed by patients, colleagues, and self-evaluation compared with anaesthesiologists with only clinical training.^84^

#### Training in ultrasound-assisted or guided procedures

Simulation-based training in ultrasound-assisted and ultrasound-guided lumbar puncture effectively improved procedural confidence compared with pre-intervention.^85^ Short sessions combining brief theory and up to 1.5 hours of hands-on practise were sufficient for skill development.^86^^,^^87^ Blended learning, incorporating online support alongside traditional methods, was shown to be superior compared with traditional learning alone for ultrasound-guided spinal anaesthesia in one study.^88^ Physicians trained in simulation-based settings through a structured curriculum or using mastery learning were able to successfully transfer these skills to patient procedures.^89^^,^^90^

### Learning curves

Learning curves for neuraxial anaesthesia procedures, including spinal, epidural, and combined spinal–epidural anaesthesia, have been characterised to identify the clinical procedural volume required to achieve competence via the cumulative sum analysis (CUSUM).^91^, ^92^, ^93^, ^94^, ^95^, ^96^, ^97^, ^98^, ^99^ Studies found that residents required between 45 and 71 attempts at spinal anaesthesia and between 46 and 90 attempts at epidural anaesthesia to achieve competency. Proficiency, defined as maintaining a failure rate of ≤10%, may require up to 75 epidural anaesthesia attempts. However, individual variability was noted, with some residents never reaching proficiency.^91^, ^92^, ^93^^,^^95^^,^^97^ Competence in combined spinal–epidural procedures was achieved after approximately 40 attempts in most residents, who had no prior experience in performing epidurals, based on a predefined acceptable failure rate of 20%.^99^ Preprocedural ultrasound increased success rates in epidural anaesthesia within the first 60 procedures compared with traditional techniques without using preprocedural ultrasound.^94^

### Assessment tools

Eight of the included studies gathered validity evidence for assessment tools.^100^, ^101^, ^102^, ^103^, ^104^, ^105^, ^106^, ^107^ Three of these focused on infant lumbar puncture in a simulation-based setting, gathering validity evidence for tools using the Objective Structured Assessment of Technical Skills (OSATS) scoring or other global rating scales.^101^^,^^102^^,^^104^ Two studies investigated assessment tools for adult lumbar puncture: one using a novel virtual-reality simulator with integrated metrics, and the other a low-fidelity phantom.^103^^,^^106^ One study developed an assessment tool with validity evidence for epidural anaesthesia in a clinical setting based on a global rating scale and a checklist.^107^ Likewise, one study gathered validity evidence for an assessment tool to discriminate proficiency between novices and experienced practitioners in spinal anaesthesia in a simulation-based setting.^105^ Finally, one study developed and gathered validity evidence for an assessment tool for regional anaesthesia, including both epidural and spinal anaesthesia. This assessment tool was explored in a clinical setting.^100^ No studies assessing ultrasound-assisted or guided skills in neuraxial space access were retrieved.

Another approach to assessment was explored in a study that examined both self-assessment and supervisor assessment of infant lumbar puncture performance. The study found a moderate agreement between the two evaluations.^108^

### Outcomes

Studies included either one outcome (*n*=69) or multiple (*n*=30). The most used measurement tool was Likert-based self-assessment (*n*=43), followed by clinical metrics (*n*=33) and procedural checklists (*n*=27), but seven categories of outcome assessment tools were presented (Supplementary Appendix 5). In terms of Kirkpatrick levels for outcomes, level 1 was the most prevalent (*n*=57), followed by level 2 (*n*=34), level 4 (*n*=34), and level 3 (*n*=4) (Supplementary Appendix 5).

### Medical Education Research Study Quality Instrument

The total Medical Education Research Study Quality Instrument (MERSQI) scores varied across studies (Supplementary Appendix 6), with a mean score of 11.6 points and a range from 6.5 to the maximum of 18 points (Supplementary Appendix 7). Twenty-four studies had a single-group post-test-only design, whereas the majority (*n*=39) used a single-group pre-test and post-test design. Twelve studies were classified as non-RCTs, and 24 studies were RCTs. A low score was often given in terms of including only one institution (*n*=88), and for only using subjective assessments (*n*=41).

Most studies (*n*=51) did not report any of the three sources of validity evidence for instrument evaluation included in MERSQI, whereas 17 studies reported only content validity. Additionally, 31 studies reported more than one type of validity evidence. Overall, 28 of the included studies were of low quality with a MERSQI score <9, whereas only 22 studies were of high quality with a MERSQI score of ≥14.

## Discussion

This scoping review summarises the various neuraxial educational strategies, which have been described to date in 99 studies. The studies are heterogeneous in terms of educational intervention, participant assessment, outcomes, and quality. Despite this, certain trends occur across the studies. The following discussion is based on adequate to high-quality studies.

### Low- and high-fidelity manikins

In general, training was based on low-fidelity manikins, with theoretical instruction before hands-on practise. In simulation-based training, the discussion between low-fidelity training and high-fidelity training centres on balancing realism, cost, accessibility, and learning outcomes. A single RCT that compared anaesthesiologists’ learning epidural placement on low-fidelity manikins *vs* high-fidelity manikins showed no difference in outcome.^82^ This is supported by multiple studies focusing on technical skills acquisition in other medical procedures.^109^, ^110^, ^111^, ^112^ These findings suggest that, despite the enhanced realism of high-fidelity manikins, they may not fully replicate the clinical environment. Likewise, a study has compared training on manikins with cadaver training, showing no significant difference in achieved technical skills.^113^ These findings support that it is the quality of the training environment, rather than its fidelity, which plays an important role in technical skills training (Table 1). Emerging technologies, such as augmented reality, have been explored to enhance training by providing real-time anatomical visualisation in neuraxial space access; however, sparse evidence exists to determine its position relative to low- and high-fidelity training.^114^

**Table 1:** Discussion summary table.

Discussion topic	Conclusions	Remaining research questions
Low- *vs* high-fidelity manikins	The quality of the training environment appears more important than the fidelity level in technical neuraxial skills training, as studies show no significant differences between low- and high-fidelity models or between manikins and cadavers	• Are there specific procedural skills or learner experience levels where training fidelity has a greater impact on outcomes?
Instructor-led- and self-directed training	Instructor-led training provides structured feedback and has shown immediate benefits in skill acquisition, but self-directed training may support better long-term skill retention in neuraxial access skills	• Are there specific learner characteristics that influence whether instructor-led or self-directed training is more effective? • How does the combination of instructor-led and self-directed training impact long-term skill retention?
Proficiency-based training and skills assessment	Proficiency-based training, particularly mastery learning, is an effective method for achieving competence in neuraxial access, accommodating individual learning differences better than time- or procedure-based approaches	• How can assessment tools be further developed to better evaluate ultrasound-assisted neuraxial access skills?
	The development of valid assessment tools remains critical for ensuring competence in both traditional and ultrasound-assisted techniques	
Skill retention	Skill retention after technical training tends to decline over time, but just-in-time training shows potential in preventing this loss by refreshing skills just before they are needed in practise. Variability in retention rates may depend on factors such as skill type, training intensity, and follow-up duration	• What are the optimal intervals and methods for delivering brush-up training to maintain skill retention in neuraxial access skills?
Self-assessment	Although self-assessments are common, there is a clear need for more research using objective clinical metrics to better evaluate skills and outcomes	• Research at Kirkpatrick level 4 is needed to demonstrate improved patient outcomes from skill acquisition in simulation-based training for neuraxial access.

### Instructor-led and self-directed training

Another trend was studies based on instructor-led training. Instructor-led training remains a foundational approach in medical education, offering structured guidance and feedback from a senior colleague or an experienced clinician with in-depth knowledge of the specific procedure that self-directed learning methods lack. Instructor-led training has proven to be superior in increasing practical skills immediately after a training intervention in terms of basic life support compared with self-directed training.^115^ However, in terms of training in neuraxial space access, a few studies compared instructor-led training with either peer training or self-directed training and found it to be equally effective in skills acquisition.^19^^,^^27^^,^^63^

In terms of securing skills retention months after the training sessions, self-directed training proved to result in longer skills retention in lumbar puncture,^63^ which is likewise found in self-directed basic life support in comparison with instructor-led training.^115^ In terms of self-directed training as noninferior to instructor-led training, this is also supported in research in other medical procedural skills acquisition.^116^^,^^117^ However, an advantage of instructor-led training may be the possibility of performing deliberate practise, which was often seen in the included literature. Deliberate practise is repetitive practise with immediate feedback, enabling learners to target specific areas for improvement and gradually achieve improvement. Deliberate practise proved useful on multiple outcomes when training neuraxial space access skills.^15^^,^^20^^,^^28^^,^^29^^,^^37^^,^^41^^,^^43^^,^^45^^,^^52^^,^^56^ In terms of the time for feedback during the learning session, a single study on novices learning spinal anaesthesia found end-of-task feedback to be more effective than continuous in-task feedback for securing both short-term and long-term procedural skills retention (Table 1).^118^

### Proficiency-based training and skills assessment

Proficiency-based training was an effective educational strategy used across all neuraxial space access procedures.^15^^,^^41^^,^^45^^,^^52^^,^^61^^,^^70^, ^71^, ^72^^,^^79^ Alternatives to proficiency-based training include time-based training (training based on the duration of the training), and procedure-based training (training based on completing a specific number of tasks). Proficiency-based training, including mastery learning, has proved a valuable tool in achieving competencies in multiple systematic reviews.^119^^,^^120^ Likewise, improvement in clinical performance after a structured simulation-based mastery learning has been shown and with this, a reduction in complications.^121^^,^^122^

Studies of neuraxial anaesthesia training demonstrated large inter-individual variability in learning curves, further emphasising the importance of proficiency-based approaches.^91^, ^92^, ^93^, ^94^, ^95^, ^96^, ^97^, ^98^, ^99^ The variability presented in these studies highlights the limitations of time- or procedure-based training approaches, where fixed thresholds fail to account for individual differences in skills acquisition.^123^ Proficiency-based training, including mastery-based learning, offers a more flexible framework by requiring trainees to meet predefined competency benchmarks before progressing, ensuring all trainees achieve competence while accommodating individual learning curves.^124^ Multiple studies indicate that proficiency training should be standard which is also supported by evidence regarding other procedures such as chest tube insertion and flexible bronchoscopy.^70^^,^^71^^,^^90^^,^^125^^,^^126^

An important component of proficiency-based training is the use of assessment tools with solid validity evidence to objectively measure individual trainees’ progress and ensure that they meet competency standards. Eight of the studies included in this review focused on gathering validity evidence for assessment tools in simulation-based and clinical settings, including various assessment tools such as combined checklists, global rating scales, OSATS, and specifically named tools including the Imperial College Surgical Assessment Device for Spinal Anaesthesia (ICSAD), OSATS-LP, and the Lumbar Puncture Assessment Tool (LumPAT).^100^, ^101^, ^102^, ^103^, ^104^, ^105^, ^106^, ^107^ Increasing the focus on mastery learning, through valid assessment tools, is further supported by a recently published editorial, which also highlights the importance of ensuring the reduction of inter-individual rater variability through mastery training in regional anaesthesia.^127^

As the field of medical education continues to evolve, the development and exploration of assessment tools will be continuously important in maintaining high standards of competence. Several assessment tools with solid evidence of validity exist for neuraxial space access but a notable gap exists in the availability of tools to assess the competence of physicians performing ultrasound-assisted and ultrasound-guided access (Table 1).

### Skills retention

When acquiring a new technical skill, loss of retention is a recognised challenge. Studies show that without a brush-up training, skills may deteriorate within a few months after initial training.^128^^,^^129^ This was evident in several studies within this review, which reported a notable decline in skill retention over time, though the extent of this loss varied considerably across studies.^37^^,^^45^^,^^52^ Such variation could be attributed to differences in skill type, training intensity, and follow-up periods, highlighting the complexity of retention dynamics. A few of the included studies examined just-in-time training.^71^^,^^74^^,^^75^ By refreshing the learner’s knowledge and skills shortly before they are needed in practise, just-in-time training may help counteract the decline of skills. Findings suggest that just-in-time training could be a feasible strategy to minimise skill decay before clinical performance (Table 1).^71^^,^^74^^,^^75^

### Self-assessment

The predominant use of Likert-based self-assessments in the included studies underlines a need for more research with a focus on objective and clinical metrics. Although self-assessments provide insights into trainee confidence and perceptions, the integration of objective clinical metrics and procedural checklists enhances the reliability of outcome evaluations. Kirkpatrick level 1, being self-assessment, is generally not recommended in medical education studies, as studies of self-assessment have shown that self-assessed abilities are uncorrelated with actual performance measures.^130^^,^^131^ Although satisfaction and skills were commonly assessed, fewer studies evaluated the impact on patient outcomes, highlighting the need for more studies that follow trainees into clinical practise to determine the skills transfer to patient treatment (Table 1).

### Medical Education Research Study Quality Instrument

Of the included literature, 28 were of low quality, with a MERSQI score <9, and only 22 studies were of high quality with a MERSQI score of ≥14. A total MERSQI score <9 is predicted to be rejected by journal editors,^132^ and a score of 14 or more is rated as high quality.^133^ As most studies did not report any of the three sources of validity evidence for instrument evaluation included in MERSQI, this also supports the need for assessment tools with solid evidence of validity, highlighting that the interpretation of study results should be considered in the given context of study, and cannot be generalised to all settings.

### Strengths and limitations

This scoping review presents a comprehensive overview of existing literature on training in neuraxial space access skills, representing the first review on this topic. A strength of this review is the comprehensive search strategy, which resulted in a relatively large number of studies. The thoroughness of the search was sustained by the review of citations and references in the included articles, which did not uncover any additional studies. However, this review also highlights certain limitations. The heterogeneity of the included studies and a rather low methodological quality presented challenges in synthesising results. Study variabilities were observed in multiple aspects, such as the training interventions and the range of educational outcomes measured. The existing metrics to evaluate neuraxial space access are weak, their reliability is untested, and the psychometrics have not been evaluated. Further high-quality research is needed, especially at Kirkpatrick level 4, to provide evidence of improved patient outcomes resulting from skill acquisition in neuraxial space access.

### Conclusions

Published studies on education and training in neuraxial space access show substantial variation in educational approaches, assessment methods, and study quality. Despite this, consistent trends indicate that simulation-based training enhances outcomes across multiple Kirkpatrick levels. Future educational programmes should start with a theoretical introduction, followed by training on low-fidelity manikins with a focus on deliberate practise. Training should be proficiency-based, requiring all trainees to continue practising until skills are ensured using an assessment tool with solid evidence of validity. To address skill retention, self-directed training or just-in-time training may be beneficial before clinical performance. Further high-quality research, particularly at Kirkpatrick level 4, is needed to demonstrate improved patient outcomes from skill acquisition in simulation-based training for neuraxial space access.

## Authors’ contributions

Conceptualisation: MSN, AMG, ABN, LK, ACB

Data collection: MSN, FVI, ACB

Formal analysis: MSN

Writing - original draft: MSN, ACB

Writing - review and editing: all authors

## Declaration of interest

The authors declare that they have no conflicts of interest.

## References

[1] de Filho G.R., Gomes H.P., da Fonseca M.H., Hoffman J.C., Pederneiras S.G., Garcia J.H. (2002). Predictors of successful neuraxial block: a prospective study. Eur J Anaesthesiol.

[2] Chen H., Kim R., Perret D., Hata J., Rinehart J., Chang E. (2016). Improving trainee competency and comfort level with needle driving using simulation training. Pain Med.

[3] Howard S.C., Gajjar A.J., Cheng C. (2002). Risk factors for traumatic and bloody lumbar puncture in children with acute lymphoblastic leukemia. JAMA.

[4] (2016). Practice guidelines for obstetric anesthesia: an updated report by the American Society of Anesthesiologists Task Force on Obstetric Anesthesia and the Society for Obstetric Anesthesia and Perinatology. Anesthesiology.

[5] Association of Anaesthetists (2010). Best practise in the management of epidural analgesia in the hospital setting.

[6] Engelborghs S., Niemantsverdriet E., Struyfs H. (2017). Consensus guidelines for lumbar puncture in patients with neurological diseases. Alzheimers Dement (Amst).

[7] Tumani H., Petereit H.F., Gerritzen A. (2020). S1 guidelines "lumbar puncture and cerebrospinal fluid analysis" (abridged and translated version). Neurol Res Pract.

[8] Vilmann P., Clementsen P.F., Colella S. (2015). Combined endobronchial and esophageal endosonography for the diagnosis and staging of lung cancer: European Society of Gastrointestinal Endoscopy (ESGE) Guideline, in cooperation with the European Respiratory Society (ERS) and the European Society of Thoracic Surgeons (ESTS). Endoscopy.

[9] Laursen C.B., Clive A., Hallifax R. (2021). European Respiratory Society statement on thoracic ultrasound. Eur Respir J.

[10] Moher D., Shamseer L., Clarke M. (2015). Preferred reporting items for systematic review and meta-analysis protocols (PRISMA-P) 2015 statement. Syst Rev.

[11] Tricco A.C., Lillie E., Zarin W. (2018). PRISMA Extension for Scoping Reviews (PRISMA-ScR): checklist and explanation. Ann Intern Med.

[12] Nielsen M.S., Ilkjær F.V., Grejs A.M., Nielsen A.B., Konge L., Brøchner A.C. (2024). Training and assessment of skills in neuraxial access-protocol of a scoping review. Acta Anaesthesiol Scand.

[13] Kirkpatrick D.L., Brown S.M., Seidner C.J. (1998). Evaluating Corporate Training: Models and Issues.

[14] Reed D.A., Cook D.A., Beckman T.J., Levine R.B., Kern D.E., Wright S.M. (2007). Association between funding and quality of published medical education research. JAMA.

[15] Augustine E.M., Kahana M. (2012). Effect of procedure simulation workshops on resident procedural confidence and competence. J Grad Med Educ.

[16] Stolarek I. (2007). Procedural and examination skills of first-year house surgeons: a comparison of a simulation workshop versus 6 months of clinical ward experience alone. N Z Med J.

[17] Westwood A., Hohler A. (2012). Lumbar puncture simulation training improves medical student knowledge and confidence (P06.027). Neurology.

[18] Ciccotto G., Nelson A., Blaya M. (2012). Lumbar puncture teaching module standardization. Ensuring patient safety through proper training (P06.024). Neurology.

[19] Dadoun A., Dubrovsky A., Bhanji F., Varshney T. (2015). Peer teaching: an effective method for simulation-based instruction. Can J Emerg Med.

[20] Crichlow A., Parsons J., Goswami V., Ponnuru S., Griswold S. (2017). Integration of a simulation based mastery learning lumbar puncture curriculum using observational learning into an emergency medicine intern boot camp. Acad Emerg Med.

[21] Melamed S., Robin B. (2017). Sim one, teach one-senior resident-led pediatric intern procedural training. Acad Pediatr.

[22] Meerkov M.S., Saba T.G. (2017). A simulation-based procedural curriculum for pediatric interns improves self-perceived competence. Acad Pediatr.

[23] Fischer J.B., Saba T. (2017). A simulation-based longitudinal procedural curriculum for pediatric residents improves self perceived competence. Acad Pediatr.

[24] Wong B., Nhoung H., Liu P., Sahai-Srivastava S. (2018). Developing a child neurology training program in Cambodia: a pilot study. Neurology.

[25] Valentine D., Allen A., Mirasol R., Kurzweil A. (2019). Outcomes of a boot camp for incoming neurology residents. Neurology.

[26] Antonenko A., Shanahan E., Slattery N. (2021). Improving lumbar puncture technique among intern trainees to enhance quality of care for patients. Age Ageing.

[27] Dugan L., Rei K., Ueno A., Mahutga J., Hantz T., Varma M. (2023). Assessing best practices in a simulated lumbar puncture workshop with medical students. Neurology.

[28] Toy S., McKay R.S., Walker J.L., Johnson S., Arnett J.L. (2017). Using learner-centered, simulation-based training to improve medical students' procedural skills. J Med Educ Curr Dev.

[29] Yee J., San Miguel C., Khandelwal S., Way D.P., Panchal A.R. (2022). Procedural curriculum to verify intern competence prior to patient care. West J Emerg Med.

[30] Sattler L.A., Schuety C., Nau M. (2020). Simulation-based medical education improves procedural confidence in core invasive procedures for military internal medicine residents. Cureus.

[31] Adam A., Mangtani A., Jacobs C., Daniel J. (2022). Clinical skills day: a novel approach to enhancing procedural skills teaching for foundation year one doctors. Cureus.

[32] Yanta C., Knepper L., Van Deusen R., Ruppert K. (2020). The use of hybrid lumbar puncture simulation to teach entrustable professional activities during a medical student neurology clerkship. MedEdPublish.

[33] Oxentenko A.S., Ebbert J.O., Ward L.E., Pankratz V.S., Wood K.E. (2003). A multidimensional workshop using human cadavers to teach bedside procedures. Teach Learn Med.

[34] Ocel J.J., Natt N., Tiegs R.D., Arora A.S. (2006). Formal procedural skills training using a fresh frozen cadaver model: a pilot study. Clin Anat.

[35] Patel M., Oosthuizen G., Child S., Windsor J.A. (2008). Training effect of skills courses on confidence of junior doctors performing clinical procedures. N Z Med J.

[36] Lenhard A., Moallem M., Marrie R.A., Becker J., Garland A. (2008). An intervention to improve procedure education for internal medicine residents. J Gen Intern Med.

[37] Conroy S.M., Bond W.F., Pheasant K.S., Ceccacci N. (2010). Competence and retention in performance of the lumbar puncture procedure in a task trainer model. Simul Healthc.

[38] Garrood T., Iyer A., Gray K. (2010). A structured course teaching junior doctors invasive medical procedures results in sustained improvements in self-reported confidence. Clin Med (Lond).

[39] Adachi K., Yoshimura A., Aso R. (2012). Clinical clerkship course for medical students on lumbar puncture using simulators. J Nippon Med Sch.

[40] Brydges R., Fiume A., Grierson L. (2022). Mastery versus invention learning: impacts on future learning of simulated procedural skills. Adv Health Sci Educ Theory Pract.

[41] Barsuk J.H., Cohen E.R., Caprio T., McGaghie W.C., Simuni T., Wayne D.B. (2012). Simulation-based education with mastery learning improves residents' lumbar puncture skills. Neurology.

[42] Cohen E.R., Barsuk J.H., Moazed F. (2013). Making July safer: simulation-based mastery learning during intern boot camp. Acad Med.

[43] Wayne D.B., Cohen E.R., Singer B.D. (2014). Progress toward improving medical school graduates' skills via a "boot camp" curriculum. Simul Healthc.

[44] Goolsby C.A., Goodwin T.L., Vest R.M. (2014). Hybrid simulation improves medical student procedural confidence during EM clerkship. Mil Med.

[45] Yeo C.T., Davison C., Ungi T., Holden M., Fichtinger G., McGraw R. (2015). Examination of learning trajectories for simulated lumbar puncture training using hand motion analysis. Acad Emerg Med.

[46] Katz L.M., Finch A., McKinnish T., Gilliland K., Tolleson-Rinehart S., Marks B.L. (2017). Teaching procedural skills to medical students: a pilot procedural skills lab. Educ Health (Abingdon).

[47] Shammari A., Inayah A., Afsar N.A. (2018). Evaluation of effectiveness of a paediatric simulation course in procedural skills for paediatric residents — a pilot study. J Pak Med Assoc.

[48] Cheung J.J.H., Kulasegaram K.M., Woods N.N., Brydges R. (2019). Why content and cognition matter: integrating conceptual knowledge to support simulation-based procedural skills transfer. J Gen Intern Med.

[49] von Cranach M., Backhaus T., Brich J. (2019). Medical students' attitudes toward lumbar puncture—and how to change. Brain Behav.

[50] Vusse L.V., Shepherd A., Bergam B., Andros J., Morris A. (2020). Procedure training workshop for internal medicine residents that emphasizes procedural ultrasound: logistics and teaching materials. MedEdPORTAL.

[51] Roehr M., Wu T., Maykowski P. (2021). The feasibility of virtual reality and student-led simulation training as methods of lumbar puncture instruction. Med Sci Educ.

[52] Hale C., Crocker J., Vanka A., Ricotta D.N., McSparron J.I., Huang G.C. (2021). Cohort study of hospitalists' procedural skills: baseline competence and durability after simulation-based training. BMJ Open.

[53] Boggs Z.D., Regalado L.E., Makary M.S. (2022). Procedural fundamentals for medical students: institutional outcomes of a novel multimodal course. Acad Radiol.

[54] Huang X., Yan Z., Gong C. (2023). A mixed-reality stimulator for lumbar puncture training: a pilot study. BMC Med Educ.

[55] Gaubert S., Blet A., Dib F. (2021). Positive effects of lumbar puncture simulation training for medical students in clinical practice. BMC Med Educ.

[56] Galen B., Conigliaro R. (2019). A curriculum for lumbar puncture training in internal medicine residency. MedEdPublish.

[57] Lilamand M., Vrillon A., Gonzales-Marabal L. (2023). Lumbar puncture training with healthcare simulation improves self-confidence and practical skills of French medical residents in geriatrics. Eur Geriatr Med.

[58] Sun C., Qi X. (2018). Evaluation of problem- and simulator-based learning in lumbar puncture in adult neurology residency training. World Neurosurg.

[59] Lenchus J., Issenberg S.B., Murphy D. (2011). A blended approach to invasive bedside procedural instruction. Med Teach.

[60] Munoz-Leija D., Diaz Gonzalez-Colmenero F., Ramirez-Mendoza D.A. (2024). Development and evaluation of an in-house lumbar puncture simulator for first-year resident lumbar puncture procedure learning. Cureus.

[61] Mourad M., Ranji S., Sliwka D. (2012). A randomized controlled trial of the impact of a teaching procedure service on the training of internal medicine residents. J Grad Med Educ.

[62] Burgess B., Nichols W., Jasani N., Reed J. (2011). Implementation of a lumbar puncture (LP) simulation teaching module. J Emerg Med.

[63] Brydges R., Nair P., Ma I., Shanks D., Hatala R. (2012). Directed self-regulated learning versus instructor-regulated learning in simulation training. Med Educ.

[64] Gaies M.G., Morris S.A., Hafler J.P. (2009). Reforming procedural skills training for pediatric residents: a randomized, interventional trial. Pediatrics.

[65] Vassallo J.C., Gouguenheim B., Ghiglione A. (2015). Lumbar puncture training using simulation-based educational strategies: experience in a clinical pediatric residency. Arch Argent Pediatr.

[66] McMillan H.J., Writer H., Moreau K.A. (2016). Lumbar puncture simulation in pediatric residency training: improving procedural competence and decreasing anxiety. BMC Med Educ.

[67] Elhadi M., Ahmed H., Khaled A. (2020). Informed self-assessment versus preceptor evaluation: a comparative study of pediatric procedural skills acquisition of fifth year medical students. BMC Med Educ.

[68] White M.L., Jones R., Zinkan L., Tofil N.M. (2012). Transfer of simulated lumbar puncture training to the clinical setting. Pediatr Emerg Care.

[69] Kilbane B.J., Adler M.D., Trainor J.L. (2010). Pediatric residents' ability to perform a lumbar puncture: evaluation of an educational intervention. Pediatr Emerg Care.

[70] Kessler D.O., Auerbach M., Pusic M., Tunik M.G., Foltin J.C. (2011). A randomized trial of simulation-based deliberate practice for infant lumbar puncture skills. Simul Healthc.

[71] Auerbach M., Fein D.M., Chang T.P. (2016). The correlation of workplace simulation-based assessments with interns' infant lumbar puncture success: a prospective, multicenter, observational study. Simul Healthc.

[72] Lydon S., McDermott B., Ryan E. (2019). Can simulation-based education and precision teaching improve paediatric trainees' behavioural fluency in performing lumbar puncture? A pilot study. BMC Med Educ.

[73] Kessler D.O., Arteaga G., Ching K. (2013). Interns' success with clinical procedures in infants after simulation training. Pediatrics.

[74] Kessler D.O., Chang T.P., Auerbach M. (2017). Screening residents for infant lumbar puncture readiness with just-in-time simulation-based assessments. BMJ Simul Technol Enhanc Learn.

[75] Guerra-Wallace M.S.N., Manole M. (2010). “Just-in-Time” Lumbar Puncture Simulation Training for Residents in the Pediatric Emergency Department. Pediatr Emerg Care.

[76] Goldman M.P., Rudd A.V., Baum S.C. (2022). Formative assessments promote procedural learning and engagement for senior pediatric residents on rotation in the pediatric emergency department. MedEdPORTAL.

[77] Goldman M.P., Palladino L.E., Malik R.N. (2022). A workplace procedure training cart to augment pediatric resident procedural learning. Pediatr Emerg Care.

[78] Srinivasan K.K., Gallagher A., O'Brien N. (2018). Proficiency-based progression training: an 'end to end' model for decreasing error applied to achievement of effective epidural analgesia during labour: a randomised control study. BMJ Open.

[79] Mohamed H., McAuliffe N., O'Connor R. (2021). Proficiency-based progression training: implementing a novel approach to training for epidural analgesia in labour. Int J Obstet Anesth.

[80] Wiggins L.L., Morrison S., Lutz C., O'Donnell J. (2018). Using evidence-based best practices of simulation, checklists, deliberate practice, and debriefing to develop and improve a regional anesthesia training course. AANA J.

[81] Udani A.D., Macario A., Nandagopal K., Tanaka M.A., Tanaka P.P. (2014). Simulation-based mastery learning with deliberate practice improves clinical performance in spinal anesthesia. Anesthesiol Res Pract.

[82] Friedman Z., Siddiqui N., Katznelson R., Devito I., Bould M.D., Naik V. (2009). Clinical impact of epidural anesthesia simulation on short- and long-term learning curve: high- versus low-fidelity model training. Reg Anesth Pain Med.

[83] Bisgaard C.H., Rodt S.A., Musaeus P., Petersen J.A.K., Rubak S.L.M. (2021). Early procedural training increases anesthesiology residents' clinical production: a comparative pre-post study of the payoff in clinical training. BMC Med Educ.

[84] Sadat M.S., Morteza K., Mohammad M.S., Shahram S. (2024). Effects of a novel blended virtual reality and clinical learning environment on the learning transfer of anesthesiology residents. Acta Medica Iranica.

[85] Gandhi T., Northcote A., Bloom G.B., Goraya H. (2023). Ultrasound-assisted lumbar puncture: a quality improvement project. Chest.

[86] Restrepo C.G., Baker M.D., Pruitt C.M., Gullett J.P., Pigott D.C. (2015). Ability of pediatric emergency medicine physicians to identify anatomic landmarks with the assistance of ultrasound prior to lumbar puncture in a simulated obese model. Pediatr Emerg Care.

[87] Krause C., Krause R., Dinh V. (2016). Utilizing a multimodal approach in teaching medical students ultrasound-guided procedures. J Investig Med.

[88] Wang T., Zhou Y., Xu M., Deng Y. (2024). Continuing medical education for attending physicians in anesthesia: feasibility of an innovative blended learning approach. Medicine (Baltimore).

[89] Shaikh F., Arzola C., Alexander S. (2021). Feasibility of ultrasound-assisted lumbar punctures performed by pediatric oncologists at the point of care. Pediatr Blood Cancer.

[90] Williams J. (2018). Simulation-based mastery learning improves lumbar puncture but not paracentesis performance. J Hosp Med.

[91] Kopacz D.J., Neal J.M., Pollock J.E. (1996). The regional anesthesia "learning curve". What is the minimum number of epidural and spinal blocks to reach consistency?. Reg Anesth.

[92] Konrad C., Schupfer G., Wietlisbach M., Gerber H. (1998). Learning manual skills in anesthesiology: is there a recommended number of cases for anesthetic procedures?. Anesth Analg.

[93] Naik V.N., Devito I., Halpern S.H. (2003). Cusum analysis is a useful tool to assess resident proficiency at insertion of labour epidurals. Can J Anaesth.

[94] Grau T., Bartusseck E., Conradi R., Martin E., Motsch J. (2003). Ultrasound imaging improves learning curves in obstetric epidural anesthesia: a preliminary study. Can J Anaesth.

[95] Guasch E., Díez J., Gilsanz F. (2010). [Monitoring skill acquisition in obstetric epidural puncture at a university hospital using the cumulative sum method]. Rev Esp Anestesiol Reanim.

[96] Ospina O.D.A., Medina Á.M.R., Marulanda M.C., Buitrago L.M.G. (2014). Cumulative Sum learning curves (CUSUM) in basic anaesthesia procedures. Colomb J Anesthesiol.

[97] Drake E.J., Coghill J., Sneyd J.R. (2015). Defining competence in obstetric epidural anaesthesia for inexperienced trainees. Br J Anaesth.

[98] Weil G., Motamed C., Biau D.J., Guye M.L. (2017). Learning curves for three specific procedures by anesthesiology residents using the learning curve cumulative sum (LC-CUSUM) test. Korean J Anesthesiol.

[99] Lew E., Allen J.C., Goy R.W.L., Ithnin F., Sng B.L. (2020). Determining competence in performing obstetric combined spinal-epidural procedures in junior anesthesiology residents: results from a cumulative sum analysis. Int J Obstet Anesth.

[100] Chuan A., Graham P.L., Wong D.M. (2015). Design and validation of the Regional Anaesthesia Procedural Skills Assessment Tool. Anaesthesia.

[101] Iyer M.S., Santen S.A., Nypaver M. (2013). Assessing the validity evidence of an objective structured assessment tool of technical skills for neonatal lumbar punctures. Acad Emerg Med.

[102] Gerard J.M., Kessler D.O., Braun C., Mehta R., Scalzo A.J., Auerbach M. (2013). Validation of global rating scale and checklist instruments for the infant lumbar puncture procedure. Simul Healthc.

[103] Henriksen M.J.V., Wienecke T., Thagesen H. (2017). Assessment of residents readiness to perform lumbar puncture: a validation study. J Gen Intern Med.

[104] Braun C., Kessler D.O., Auerbach M., Mehta R., Scalzo A.J., Gerard J.M. (2017). Can residents assess other providers' infant lumbar puncture skills?: validity evidence for a global rating scale and subcomponent skills checklist. Pediatr Emerg Care.

[105] Corvetto M.A., Fuentes C., Araneda A. (2017). Validation of the imperial college surgical assessment device for spinal anesthesia. BMC Anesthesiol.

[106] Xie S., Grimstrup S., Nayahangan L.J., Wang Z., Wan X., Konge L. (2023). Using a novel virtual-reality simulator to assess performance in lumbar puncture: a validation study. BMC Med Educ.

[107] Friedman Z., Katznelson R., Devito I., Siddiqui M., Chan V. (2006). Objective assessment of manual skills and proficiency in performing epidural anesthesia-video-assisted validation. Reg Anesth Pain Med.

[108] Reinhardt J.C., Grossman D., Kunkov S., Thode H. (2012). 4 intern self-assessment and prediction of lumbar puncture success. Ann Emerg Med.

[109] Tan S.C., Marlow N., Field J. (2012). A randomized crossover trial examining low- versus high-fidelity simulation in basic laparoscopic skills training. Surg Endosc.

[110] Saito S., Endo K., Sakuma Y., Sata N., Lefor A.K. (2023). Simulator fidelity does not affect training for robot-assisted minimally invasive surgery. J Clin Med.

[111] Finan E., Bismilla Z., Whyte H.E., Leblanc V., McNamara P.J. (2012). High-fidelity simulator technology may not be superior to traditional low-fidelity equipment for neonatal resuscitation training. J Perinatol.

[112] Nimbalkar A., Patel D., Kungwani A., Phatak A., Vasa R., Nimbalkar S. (2015). Randomized control trial of high fidelity vs low fidelity simulation for training undergraduate students in neonatal resuscitation. BMC Res Notes.

[113] Huri G., Gülşen M.R., Karmış E.B., Karagüven D. (2021). Cadaver versus simulator based arthroscopic training in shoulder surgery. Turk J Med Sci.

[114] McLeod G., McKendrick M., Portelli R., Husz Z., James G., McKendrick G. (2025). Hologram-assisted thoracic epidural insertion in the Thiel soft embalmed cadaver: proof of concept simulation study. Br J Anaesth.

[115] Bylow H., Karlsson T., Claesson A., Lepp M., Lindqvist J., Herlitz J. (2019). Self-learning training versus instructor-led training for basic life support: a cluster randomised trial. Resuscitation.

[116] Nematian H., Masoumnia A.M., Shakiba S. (2023). Comparison of instructor-led and video-based instruction in teaching suturing to medical students. J Surg Res.

[117] Vestergaard L.D., Løfgren B., Jessen C.L. (2017). A comparison of pediatric basic life support self-led and instructor-led training among nurses. Eur J Emerg Med.

[118] Lean L.L., Hong R.Y.S., Ti L.K. (2017). End-task versus in-task feedback to increase procedural learning retention during spinal anaesthesia training of novices. Adv Health Sci Educ Theory Pract.

[119] Cook D.A., Brydges R., Zendejas B., Hamstra S.J., Hatala R. (2013). Mastery learning for health professionals using technology-enhanced simulation: a systematic review and meta-analysis. Acad Med.

[120] Mazzone E., Puliatti S., Amato M. (2021). A systematic review and meta-analysis on the impact of proficiency-based progression simulation training on performance outcomes. Ann Surg.

[121] Barsuk J.H., Cohen E.R., Potts S. (2014). Dissemination of a simulation-based mastery learning intervention reduces central line-associated bloodstream infections. BMJ Qual Saf.

[122] Cohen E.R., Feinglass J., Barsuk J.H. (2010). Cost savings from reduced catheter-related bloodstream infection after simulation-based education for residents in a medical intensive care unit. Simul Healthc.

[123] Mahmood O., Jørgensen R., Nielsen K., Konge L., Russell L. (2022). Hands-on time in simulation-based ultrasound training - a dose-related response study. Ultrasound Int Open.

[124] McGaghie W.C. (2015). Mastery learning: it is time for medical education to join the 21st century. Acad Med.

[125] De Mol L., Van Herzeele I., Van de Voorde P. (2025). A structured simulation-based mastery learning curriculum in chest tube insertion results in superior skills compared to traditional training programs. World J Surg.

[126] Cold K.M., Wei W., Agbontaen K., Singh S., Konge L. (2025). Mastery learning guided by artificial intelligence is superior to directed self-regulated learning in flexible bronchoscopy training: an RCT. Respiration.

[127] McLeod G., Chuan A., McKendrick M. (2024). Attaining expertise in regional anaesthesia training using a multifactorial approach incorporating deliberate practice. Br J Anaesth.

[128] Nielsen M.S., Lundorff S.H., Hansen P.M. (2024). Anesthesiologists' skills in emergency cricothyroidotomy mandate a brush-up training after 3 months-a randomized controlled trial. Acta Anaesthesiol Scand.

[129] Nielsen M.S., Raben-Levetzau F.N., Andersen S.A.W., Wennervaldt K., Konge L., Nielsen A.B. (2023). Retention of emergency cricothyroidotomy skills: a multicenter randomized controlled trial. AEM Educ Train.

[130] Eva K.W., Regehr G. (2005). Self-assessment in the health professions: a reformulation and research agenda. Acad Med.

[131] Norman G. (2014). Data dredging, salami-slicing, and other successful strategies to ensure rejection: twelve tips on how to not get your paper published. Adv Health Sci Educ Theory Pract.

[132] Reed D.A., Beckman T.J., Wright S.M., Levine R.B., Kern D.E., Cook D.A. (2008). Predictive validity evidence for medical education research study quality instrument scores: quality of submissions to JGIM's Medical Education Special Issue. J Gen Intern Med.

[133] Lin H., Lin E., Auditore S., Fanning J. (2016). A narrative review of high-quality literature on the effects of resident duty hours reforms. Acad Med.

