# Supplementary material for: Training and assessment of skills in neuraxial space access: a scoping review of educational approaches to lumbar puncture, epidural anaesthesia, and spinal anaesthesia
Source: Br J Anaesth. 2025 Jul 7;135(4):1026–37. doi: 10.1016/j.bja.2025.06.008 (PMC12674033; doi:10.1016/j.bja.2025.06.008)
Supplement: Multimedia component 1 [file mmc1.docx]

**Appendix 1 - search strategies**

*The Cochrane Library (Cochrane Central Register of Controlled Trials and Cochrane Database of Systematic Reviews) from inception to latest issue (7/5.24)*

#1 Mesh Descriptor: [Physicians] explode all trees

#2 Mesh Descriptor: [Students, Medical] explode all trees

#3 Mesh Descriptor: [Nurse] explode all trees

#4 Physician*.ti,ab,kw OR doctor*.ti,ab,kw OR resident*.ti,ab,kw OR intern*.ti,ab,kw OR “medical student” .ti,ab,kw

#5 (#1 OR #2 OR #3 OR #4)

#6 Mesh Descriptor: [Anesthesia, Epidural] explode all trees

#7 Mesh Descriptor: [Anesthesia, Spinal] explode all trees

#8 Mesh Descriptor: [Spinal puncture] explode all trees

#9 ("epidural anesthesia" OR "epidural anaesthesia" OR "epidural analgesia" OR "epidural blockade" OR "spinal anaesthesia" OR "spinal anesthesia" OR "spinal blockade" OR "spinal analgesia" OR neuraxial blockade OR neuraxial anaesthesia OR neuraxial anesthesia OR neuraxial acess OR "central blockade" OR central acess OR caudal anaesthesia OR caudal anesthesia OR caudal block OR subarachnoid block OR subarachnoid anaesthesia OR subarachnoid anesthesia OR intradural block OR intradural anaesthesia OR intradural anesthesia OR ultrasound assisted epidural anaesthesia OR ultrasound assisted epidural anesthesia OR ultrasound assisted epidural blockade OR ultrasound assisted epidural OR ultrasound assisted spinal anaesthesia OR ultrasound assisted spinal anesthesia OR ultrasound assisted spinal blockade OR ultrasound assisted spinal OR ultrasound assisted neuraxial blockade OR ultrasound assisted neuraxial anasthesia OR ultrasound assisted neuraxial anesthesia OR ultrasound assisted neuraxial OR ultrasound assisted central blockade OR ultrasound assisted central access OR ultrasound assisted caudal anaesthesia OR ultrasound assisted caudal anesthesia OR ultrasound assisted caudal block OR ultrasound assisted subarachnoid block OR ultrasound assisted subarachnoid anaesthesia OR ultrasound assisted subarachnoid anesthesia OR ultrasound assisted intradural block OR ultrasound assisted intradural anaesthesia OR ultrasound assisted intradural anesthesia OR "lumbar puncture" OR "spinal puncture" OR spinal tap OR intrathecal puncture OR lumbosacral puncture) .ti,ab,kw

#10 (#6 OR #7 OR #8 OR #9)

#11 Mesh Descriptor: [Education] explode all trees

#12 Mesh Descriptor: [Simulation training] explode all trees

#13 Mesh Descriptor: [Curriculum] explode all trees

#14 ("education" OR education OR training OR "skills development" OR learning OR "simulation training" OR Curricula OR "training programme" OR skills OR test OR assessment OR "mastery learning" OR teaching).ti,ab,kw

#15 (#11 OR #12 OR #13 OR #14)

#16 (#5 AND #10 AND #15)

*MEDLINE (Ovid) 1946 to date of search (7/5.24)*

#1 physician.mp. or exp physicians/

#2 doctor*.mp.

#3 resident*.mp.

#4 intern*.mp.

#5 exp Students, Medical/ or Medical student*.mp.

#6 nurse.mp. or exp Nurses/

#7 (1 OR 2 OR 3 OR 4 OR 5 OR 6)

#8 epidural anesthesia.mp. or exp anesthesia, epidural/

#9 epidural analgesia.mp. or exp analgesia, epidural/

#10 exp anesthesia, spinal/ or spinal anaesthesia.mp.

#11 spinal puncture.mp. or exp Spinal Puncture/

#12(spinal blockade or spinal analgesia or neuraxial blockade or neuraxial anaesthesia or neuraxial access or central blockade or central access or caudal anaesthesia or caudal block or subarachnoid block or subarachnoid anaesthesia or intradural block or intradural anaesthesia or ultrasound assisted epidural anaesthesia or ultrasound assisted neuraxial epidural blockade or ultrasound assisted epidural or ultrasound assisted spinal anaesthesia or ultrasound assisted spinal blockade or ultrasound assisted spinal or ultrasound assisted neuraxial access or ultrasound assisted neuraxial central blockade or ultrasound assisted neuraxial central access or ultrasound assisted caudal anaesthesia or ultrasound assisted caudal block or ultrasound assisted subarachnoid block or ultrasound assisted subarachnoid anaesthesia or ultrasound assisted intradural block or ultrasound assisted intradural anaesthesia or lumbar puncture or spinal tap or intrathecal puncture or lumbosacral puncture).mp.

#13 (8 OR 9 OR 10 OR 11 OR 12)

#14 education.mp. or exp education/

#15 simulation training.mp. or exp simulation training/

#16 curriculum.mp. or exp Curriculum/

#17 exp teaching/ or teaching.mp.

#18 (training or skills development or learning or training programme or skills or test or assessment or mastery learning).mp.

#19 (14 OR 15 OR 16 OR 17 OR 18)

#20 (7 AND 13 AND 19)

*EMBASE (Ovid) 1974 to date of search (7/5.24)*

#1 physician.mp. or exp physicians/

#2 doctor*.mp.

#3 resident*.mp.

#4 intern*.mp.

#5 exp Students, Medical/ or Medical student*.mp.

#6 nurse.mp. or exp Nurses/

#7 (1 OR 2 OR 3 OR 4 OR 5 OR 6)

#8 epidural anesthesia.mp. or exp anesthesia, epidural/

#9 epidural analgesia.mp. or exp analgesia, epidural/

#10 exp anesthesia, spinal/ or spinal anaesthesia.mp.

#11 spinal puncture.mp. or exp Spinal Puncture/

#12(spinal blockade or spinal analgesia or neuraxial blockade or neuraxial anaesthesia or neuraxial access or central blockade or central access or caudal anaesthesia or caudal block or subarachnoid block or subarachnoid anaesthesia or intradural block or intradural anaesthesia or ultrasound assisted epidural anaesthesia or ultrasound assisted neuraxial epidural blockade or ultrasound assisted epidural or ultrasound assisted spinal anaesthesia or ultrasound assisted spinal blockade or ultrasound assisted spinal or ultrasound assisted neuraxial access or ultrasound assisted neuraxial central blockade or ultrasound assisted neuraxial central access or ultrasound assisted caudal anaesthesia or ultrasound assisted caudal block or ultrasound assisted subarachnoid block or ultrasound assisted subarachnoid anaesthesia or ultrasound assisted intradural block or ultrasound assisted intradural anaesthesia or lumbar puncture or spinal tap or intrathecal puncture or lumbosacral puncture).mp.

#13 (8 OR 9 OR 10 OR 11 OR 12)

#14 education.mp. or exp education/

#15 simulation training.mp. or exp simulation training/

#16 curriculum.mp. or exp Curriculum/

#17 exp teaching/ or teaching.mp.

#18 (training or skills development or learning or training programme or skills or test or assessment or mastery learning).mp.

#19 (14 OR 15 OR 16 OR 17 OR 18)

#20 (7 AND 13 AND 19)

*Scopus (7/5.24)*

#1 (TITLE-ABS-KEY ((physician* ) OR ( doctor* ) OR ( resident* ) OR ( intern* ) OR ( "Medical students" ) OR ( nurse ))

#2 (TITLE-ABS-KEY (("epidural anesthesia") OR ("epidural anaesthesia") OR ("epidural analgesia") OR ("epidural blockade") OR ("spinal anaesthesia") OR ("spinal anesthesia") OR ("spinal blockade") OR ("spinal analgesia") OR ("neuraxial blockade") OR ("neuraxial anaesthesia") OR ("neuraxial anesthesia") OR ("neuraxial access") OR ("central blockade") OR ("central access") OR ("caudal anaesthesia") OR ("caudal anesthesia") OR ("caudal block") OR ("subarachnoid block") OR ("subarachnoid anaesthesia") OR ("subarachnoid anesthesia") OR ("intradural block") OR ("intradural anaesthesia") OR ("intradural anesthesia") OR ("ultrasound assisted epidural anaesthesia") OR ("ultrasound assisted epidural anesthesia") OR ("ultrasound assisted epidural blockade") OR ("ultrasound assisted epidural") OR ("ultrasound assisted spinal anaesthesia") OR ("ultrasound assisted spinal anesthesia") OR ("ultrasound assisted spinal blockade") OR ("ultrasound assisted spinal") OR ("ultrasound assisted neuraxial blockade") OR ("ultrasound assisted neuraxial anaesthesia") OR ("ultrasound assisted neuraxial anesthesia") OR ("ultrasound assisted neuraxial access") OR ("ultrasound assisted central blockade") OR ("ultrasound assisted central access") OR ("ultrasound assisted caudal anaesthesia") OR ("ultrasound assisted caudal anesthesia") OR ("ultrasound assisted caudal block") OR ("ultrasound assisted subarachnoid block") OR ("ultrasound assisted subarachnoid anesthesia") OR ("ultrasound assisted intradural block") OR ("ultrasound assisted intradural anaesthesia") OR ("ultrasound assisted intradural anesthesia") OR ("lumbar puncture") OR ("spinal puncture") OR ("spinal tap") OR ("intrathecal puncture") OR ("lumbosacral puncture") )

#3 (TITLE-ABS-KEY ((education ) OR ( training ) OR ( "Skills development" ) OR ( learning ) OR ( "Simulation training" ) OR ( curricula ) OR ( "Training programme" ) OR ( skills ) OR ( test ) OR ( assessment ) OR ( "Mastery learning" ) OR ( teaching ))

#4 (1 AND 2 AND 3)

*PubMed (7/5.24)*

#1 **(Physician [MeSH Terms]) OR (Physician*)) OR (Doctor*)) OR (Resident*)) OR (Intern*)) OR ("Medical student")) OR (medical students [MeSH Terms])) OR (nurse [MeSH Terms])) OR (nurse)**

**#2 (epidural anesthesia[MeSH Terms]) OR ("epidural anesthesia")) OR ("epidural anaesthesia")) OR (epidural analgesia[MeSH Terms])) OR ("epidural analgesia")) OR ("epidural blockade")) OR (spinal anesthesia[MeSH Terms])) OR ("spinal anaesthesia")) OR ("spinal anesthesia")) OR ("spinal blockade")) OR ("spinal analgesia")) OR (neuraxial blockade)) OR (neuraxial anaesthesia)) OR (neuraxial anesthesia)) OR (neuraxial access)) OR ("central blockade")) OR ("central access")) OR ("caudal anaesthesia")) OR ("caudal anesthesia")) OR ("caudal block")) OR ("subarachnoid block")) OR ("subarachnoid anaesthesia")) OR ("subarachnoid anesthesia")) OR ("intradural block")) OR ("intradural anaesthesia")) OR ("intradural anesthesia")) OR ("ultrasound assisted epidural anaesthesia"[tiab:~0])) OR ("ultrasound assisted epidural anesthesia"[tiab:~0])) OR ("ultrasound assisted epidural blockade"[tiab:~0])) OR ("ultrasound assisted epidural"[tiab:~0])) OR ("ultrasound assisted spinal anaesthesia"[tiab:~0])) OR ("ultrasound assisted spinal anesthesia"[tiab:~0])) OR ("ultrasound assisted spinal blockade"[tiab:~0])) OR ("ultrasound assisted spinal"[tiab:~0])) OR ("ultrasound assisted neuraxial blockade"[tiab:~0])) OR (ultrasound assisted neuraxial anaesthesia)) OR (ultrasound assisted neuraxial anesthesia)) OR ("ultrasound assisted neuraxial access"[tiab:~0])) OR ("ultrasound assisted central blockade"[tiab:~0])) OR ("ultrasound assisted central access"[tiab:~0])) OR ("ultrasound assisted caudal anaesthesia"[tiab:~0])) OR ("ultrasound assisted caudal anesthesia"[tiab:~0])) OR ("ultrasound assisted caudal block"[tiab:~0])) OR ("ultrasound assisted subarachnoid block"[tiab:~0])) OR ("ultrasound assisted subarachnoid anesthesia"[tiab:~0])) OR ("ultrasound assisted intradural block"[tiab:~0])) OR ("ultrasound assisted intradural anaesthesia"[tiab:~0])) OR ("ultrasound assisted intradural anesthesia"[tiab:~0])) OR ("lumbar puncture")) OR (spinal puncture[MeSH Terms])) OR ("spinal puncture")) OR ("spinal tap")) OR ("intrathecal puncture")) OR ("lumbosacral puncture")**

**#3(education [MeSH Terms]) OR (Education)) OR (training [MeSH Terms])) OR (training)) OR ("Skills development")) OR (Learning)) OR (Simulation training [MeSH Terms])) OR ("Simulation training")) OR (Curricula)) OR ("Training programme")) OR (Skills)) OR (Test)) OR (Assessment)) OR (Mastery learning)) OR (Teaching)**

**#4 (1 AND 2 AND 3)**

*CINAHL (Ebsco) (7/5.24)*

#1 "Nurse" OR (MH "Nurses+") OR (MH "Physicians+") OR (MH "Students, Medical+") OR Intern* OR resident*

#2 (MH "Anesthesia, Spinal") OR (MH "Anesthesia, Epidural") OR (MH "Analgesia, Epidural") OR (MH "Spinal Puncture") OR (epidural blockade) OR (spinal blockade) OR (spinal analgesia) OR (neuraxial blockade) OR (neuraxial anaesthesia) OR (neuraxial access) OR (central blockade) OR (central access) OR (caudal anaesthesia) OR (caudal block) OR (subarachnoid block) OR (subarachnoid anaesthesia) OR (intradural block) OR (intradural anaesthesia) OR (ultrasound assisted epidural anaesthesia) OR (ultrasound assisted epidural blockade) OR (ultrasound assisted epidural) OR (ultrasound assisted spinal anaesthesia) OR (ultrasound assisted spinal blockade) OR(ultrasound assisted spinal) OR (ultrasound assisted neuraxial blockade) OR (ultrasound assisted neuraxial anasthesia) OR (ultrasound assisted neuraxial access) OR (ultrasound assisted central blockade) OR (ultrasound assisted central access) OR (ultrasound assisted caudal anaesthesia) OR (ultrasound assisted caudal block) OR (ultrasound assisted subarachnoid block) OR (ultrasound assisted subarachnoid anaesthesia) OR (ultrasound assisted intradural block) OR (ultrasound assisted intradural anaesthesia) OR (lumbar puncture) OR (spinal tap) OR (intrathecal puncture) OR (lumbosacral puncture)

#3 (MH "Education+") OR (MH "Learning+") OR (MH "Curriculum+/ED/EV") OR (Training) OR (Skills development) OR (Simulation training) OR (Curricula) OR (Training programme) OR (Skills) OR (Test) OR (Assessment) OR (Mastery learning) OR (Teaching)
