# Supplementary material for: Training and assessment of skills in neuraxial space access: a scoping review of educational approaches to lumbar puncture, epidural anaesthesia, and spinal anaesthesia
Source: Br J Anaesth. 2025 Jul 7;135(4):1026–37. doi: 10.1016/j.bja.2025.06.008 (PMC12674033; doi:10.1016/j.bja.2025.06.008)
Supplement: Multimedia component 2 [file mmc2.docx]

| **Author** | **Title** | **Year** | **Country** | **Study design** | **Participants** | **If M.D./nurse, speciality?** | | **Number of participants enrolled** | **Number of participants completed training** | **Setting (e.g. Simulation centre, clinical department etc.)** | **Skill trained/assessed** | **Description of intervention** | **Hands-on modality** | **Primary outcome** | **Primary outcome measurement tool** | **Kirkpatrick level for outcome** | **Study conclusion (from abstract)** | **Conflicts of interest** | **MERSQI Sum** |
| --- | --- | --- | --- | --- | --- | --- | --- | --- | --- | --- | --- | --- | --- | --- | --- | --- | --- | --- | --- |
| Adachi 2012 | Clinical clerkship course for medical students on lumbar puncture using simulators | 2012 | Japan | Cohort study | Medical students | | N/A | 153 | 153 | Simulation centre | Lumbar puncture | A short lecture on indications, contraindications, complications, consent, procedural notes, evidence for the procedural notes, and a review on current evidence on the procedure was given before hands-on. An instructor first demonstrated the technique while giving medical explanations. Students then tried the LP procedure using the simulator. The instructor observed each student and evaluated the number of procedures until successful insertion or measurement of cerebrospinal fluid (CSF) pressure. The instructor provided feedback to students. After the lecture and LP simulator practice, questionnaires were administered to the students to clarify their comprehension, technical achievement, and satisfaction. | Lumbar-Kun (Lumbar Puncture Simulator) and Lumbar-Kun II (Lumbar Puncture Simulator-II) (Kyoto Kagaku, Kyoto, Japan) | The effectiveness of the lumbar puncture clerkship course in the medical education program. | 6-point Likert Scale | 1 | The lumbar puncture simulators, Lumbar-Kun and Lumbar-Kun II, achieved excellent overall impressions and represent useful tools for training in lumbar puncture procedures. In addition to the simulators, an appropriate preparatory text and a short lecture before training seemed to increase the educational effect of this lumbar puncture clerkship course for medical students. | N/A | 7 |
| Adam 2022 | Clinical Skills Day: A Novel Approach to Enhancing Procedural Skills Teaching for Foundation Year One Doctors. | 2022 | United Kingdom | Cohort study | Medical doctors | | N/A | 32 | 14 | Simulation centre | Lumbar puncture | Information and explanation about lumbar puncture before being demonstrated the skills by one of the facilitators. Then the participants could practice the procedure in a simulated setting. | Simulation model - not further elaborated | Trainees’ confidence | 10-point Likert Scale | 1 | The use of Clinical Skills Days impacted positively on the FY1 doctors’ confidence in performing certain skills. Wider implementation of this promising approach for Foundation Doctors is recommended. | N/A | 7,5 |
| AguirreOspina 2014 | Cumulative Sum learning curves (CUSUM) in basic anaesthesia procedures | 2014 | Colombia | Observational study | Medical doctors | | Anaesthesia | 4 | 4 | Clinical department | Spinal and peridural anaesthesia | Assesment of spinal and epidural procedures in 4 residents and the development of a learning curve. | Patients | To prepare and analyse psychomotor learning curves in orotracheal intubation, central venous catheterization, radial artery catheterization, and spinal and peridural anaesthesia in anaesthesiology residents | CUSUM  Spinal: (Clinical measure)  1. Time from initial puncture until needle removal: less than10min. 2. Adequate depth of anaesthesia where only conscious sedation was required. 3. No physical assistance from the instructor (verbal assistance allowed). 4. Three (3) or less attempts. Failure criterion: Failure to meet the success criteria.   Epidural anaesthesia (Clinical Measure)   1. Time elapsed between the initial puncture and the removalof the needle: Less than 10min. 2. Adequate depth of anaesthesia where only conscious sedation was required. 3. Number of attempts less than, or equal to, 3. Failure criterion: Failure to meet success criteria. | 4 | CUSUM charts are tools that allow monitoring and early detection of changes in psychomotor learning in anaesthesia, providing meaningful information in relation to quality standards. | None | 10 |
| Antonenko 2021 | Improving lumbar puncture technique among intern trainees to enhance quality of care for patients | 2021 | Ireland | Cohort study | Medical doctors | | N/A | 25 | 16 | N/A | Lumbar puncture | Group teaching followed by one simulation-based practice attempt. | Low fidelity phantom - not further elaborated | Participants perception of training course and self-assessed confidence | 5-point Likert scale | 1 | This pilot study demonstrated interest and positive feedback for the LP simulation training from novice doctors with improved self-assessed confidence. Formal development of an LP simulation training curriculum is underway. The impact of this training on patient care should be assessed in practice. | N/A | 6,5 |
| Auerbach 2016 | The correlation of workplace simulation-based assessments with interns' infant lumbar puncture success a prospective, multicentre, observational study | 2016 | United States of America | Cohort study | Medical doctors | | Paediatrics Emergency Medicine | 1215 | 297 | Clinical department | Infant lumbar puncture | Just-in-time simulation-based skills refresher, when a patient needed infant lumbar puncture: First, a video describing the indications, contraindications, complications, necessary equipment, and key steps of the infant lumbar puncture. Next, they completed a simulation-based training facilitated by experts. The simulation-based practice session continued until a predefined mastery performance standard was achieved, followed by performance on patients. | Low-fidelity phantom (BabyStap; Laerdal Medical, Stockholm, Sweden) | The correlation of the supervisors’ GRS ratings with interns’ first clinical ILP success defined as obtaining cerebrospinal fluid with fewer than 1000 red blood cells per high- power field on the first needle pass of the first attempt (without previous attempts by other providers), or when cellcount was not available. | Global rating four-point anchored scale assessment and clinical measurement | 4 | A simulation-based assessment of interns conducted in the workplace before their first ILP has some value in predicting clinical ILP success. | N/A | 15 |
| Augustine 2012 | Effect of procedure simulation workshops on resident procedural confidence and competence. | 2012 | United States of America | Cohort study | Medical doctors | | Paediatrics | 72 | 72 | Clinical department | Lumbar puncture | Instructors received a 5–10-minute orientation immediately before the workshop detailing the equipment available and educational structure for the workshop. After reviewing the Indications, contraindications, and risks of each procedure, the leader demonstrated the proper technique for each procedure with high-fidelity patient simulators. Residents subsequently took turns performing each procedure and demonstrating proper use of the equipment. Residents were given feedback and repeated the procedure until he or she was deemed competent, at the discretion of the instructor. | High-fidelity simulator (not further elaborated) | The effect of a procedure simulation workshop on self-reported procedural confidence and competence | 6-point Likert Scale | 1 | Procedure simulation workshops improve resident self-reported procedural confidence and competence, particularly for procedures that are least performed. | N/A | 9,5 |
| Barsuk 2012 | Simulation-based education with mastery learning improves residents' lumbar puncture skills | 2012 | United States of America | Cohort study | Medical doctors | | Internal Medicine Neurology | 94 | 94 | N/A | Lumbar puncture | Intervention group (simulator-trained) internal medicine residents underwent a clinical skills examination using a 21-item lumbar puncture performance checklist (pretest). Subsequently, they completed a 3-hour education session featuring the New England Journal of Medicine video on lumbar puncture, an interactive lumbar puncture demonstration, and deliberate practice with directed feedback. Immediately after the educational intervention, simulator-trained residents were required to meet or exceed a minimum passing score on a clinical skills examination (post-test) using the checklist. Residents who did not achieve the minimum passing score engaged in more deliberate practice and were retested until the minimum passing score was reached.  Neurology residents (traditionally trained) completed a lumbar puncture clinical skills examination using the simulator and 21-item checklist (baseline test) and served as a comparison group. The definition of traditional training was conventional clinical neurology residency education at participating institutions. | Low-fidelity phantom (Kyoto Kagaku) | Performance of simulator- trained and traditionally trained residents on the clinical skills examinations | Minimum passing score on a 21-item lumbar puncture checklist | 2 | Residents who completed simulation-based mastery learning showed significant improvement in lumbar puncture procedural skills. Few neurology residents (control group) were competent to perform a simulated lumbar puncture despite clinical experience with the procedure. | N/A | 15,5 |
| Bisgaard 2021 | Early procedural training increases anesthesiology residents’ clinical production: a comparative pre-post study of the payoff in clinical training | 2021 | Denmark | Cohort study | Medical doctors | | Anaesthesia | 68 | 53 | Clinical departments | Spinal and epidural blocks | A one-day, full-scale simulation course in general anesthesia scenarios was offered to the intervention group residents in the third or fourth week of residency. Procedural skills training at the departments was performed using procedural simulators. A final year resident or an anesthesiology specialist supervised the simulation-based skills training sessions in all study groups.  Three control groups received traditional competency-based education. | Low-fidelity phantom (Lumbar Puncture Simulator II M43B (Kyoto Kagaku Co., Ltd., Japan)). | The effects of early competency-based procedural training on the number of performed spinal and epidurals from the Danish Anesthesia Database before and after the intervention. | Number of performed spinal and epidurals from the Danish Anesthesia Database | 3 | Early procedural training results in more productive residents and freed specialist time for additional supervision, other clinical tasks or research. This provides empirical support for a positive correlation between early focused training and increased independent production among residents. | N/A | 15 |
| Boggs 2022 | Procedural Fundamentals for Medical Students: Institutional Outcomes of a Novel Multimodal Course | 2022 | United States of America | Cohort study | Medical students | | N/A | 69 | 25 | University and clinical department | Lumbar puncture | Didactic lecture on lumbar puncture organized into sections as follows: introduction, procedure indications and contraindications, anatomical considerations, and procedure technique. The lecture presentation was image-rich and included brief instructional videos. A simulation session was offered corresponding the topic. Hands-on sessions were approximately 1-hour long and were organized as small group sessions with a proctor to student ratio of 1:5.  Lastly, the clinical patient care experiences comprised of atleast 16 hours of training with the IR peripheral vascular access team (PVAT). The participants served as observers, assistant operators, and primary operators, with progression of responsibilities as they advanced through these session | Low-fidelity phantom (Kyoto Kagaku Co., Ltd., Kyoto, Japan) | Participants confidence | 5-point Likert Scale | 1 | The creation of a medical-student-centric procedural course is feasible and fills a potential gap in undergraduate medical education. This study demonstrated that a comprehensive multimodal course, designed to include didactic, simulation and clinical experiences, increases participant exposure to, participation with, and confidence in bedside procedural performance abilities. | N/A | 7,5 |
| Braun 2017 | Can Residents Assess Other Providers' Infant Lumbar Puncture Skills?: Validity Evidence for a Global Rating Scale and Subcomponent Skills Checklist | 2017 | United States of America | Cohort study | Students, MD | | paediatric emergency medicine, neonatology and haematology | 60 | 60 | N/A | Validation of infant lumbar puncture assessment tool | Subjects were then asked to “perform an LP on the simulator, demonstrating all of the steps that you would do on a real patient to properly perform the procedure.” Videos were rated by 6 blinded raters. | Laerdal Baby Stap ILP simulator (Laerdal Medical, Stockholm, Sweden) | To provide validity evidence for infant lumbar puncture (ILP) checklist and global rating scale (GRS) instruments when used by residents to assess simulated ILP performances and to compare these metrics to previously obtained attending rater data. | Global rating scale (GRS) | 2 | This study provides validity evidence for the checklist in- strument when used by pediatric residents to assess ILP performances. Compared with attending raters, residents appeared to over-score subjects on the GRS instrument. | None | 13,5 |
| Brydges 2012 | Directed self-regulated learning versus instructor-regulated learning in simulation training | 2012 | Canada | Randomized Controlled Trial | Medical doctors | | Internal Medicine | 43 | 23 | Simulation centre | Lumbar puncture | Participants viewed a 10-minute video showing lumbar puncture on a real patient. Immediately after watching the instructional video, participants completed a videotaped pre-test in which they performed an LP procedure on the easy simulator model. After the pre-test, participants practised according to their assigned intervention.  Directed self-regulated learning: participants used their own simulators and progressed from the easy to difficult simulator models at their own pace. Participants had access to the instructional video and could control its playback ad libitum. Participants continued to practise until they decided to complete the post-test or until 35 minutes had expired, whichever came first. Immediately after the post-test, participants were given one-to-one access to an instructor for feedback and guidance. This inter- action lasted a maximum of 15 minutes; therefore, the maximum available practice time in the Directed self-regulated learning group amounted to 50 minutes.  Instructor-regulated learning: Participants practised with an instructor, in groups, at a trainee : instructor ratio of 4 : 1. During the 35 minutes of practice, the instructor and participants decided collectively whether to use the easy or difficult models and how to use the time. Immediately after the post-test, participants were able to spend an additional 15 minutes with the instructor (this feedback was not provided on a one-to-one basis). Therefore, the maximum available practice time in the IRL group amounted to 50 minutes. | Low-fidelity phantom (Kyoto Kagaku Co., Ltd, Kyoto, Japan) | To evaluate the effectiveness of directed self-regulated learning and instructor-regulated learning, respectively, for teaching lumbar puncture using simulation | Global Rating Scale and a modified version of a checklist. | 2 | Both instructor-regulated learning and directed self-regulated learning led to improved lumbar puncture performance immediately after practice. Whereas the instructor-regulated learning group’s skills declined after 3 months, the directed self-regulated learning group’s performance was maintained, suggesting a potential long-term benefit of this training. Participants in the directed self-regulated learning group also developed a more accurate relationship between confidence and competence following practice. Further research is needed to clarify the mechanisms of self-regulated learning and its role in simulation contexts. | N/A | 15 |
| Brydges 2022 | Mastery versus invention learning: impacts on future learning of simulated procedural skills | 2022 | Canada | Randomized Controlled Trial | Medical students | | N/A | 60 | 60 | N/A | Infant lumbar puncture | Mastery Learning Condition group: Infant lumbar puncture pre-test, followed by video, demonstration and practice with feedback. Then infant lumbar puncture post-test.   Problem-solving then instruction group: Infant lumbar puncture pre-test, followed by guided intervention activity with simulator followed by video, demonstration, and practice with instructors.  Instruction the practice group:  Infant lumbar puncture pre-test, followed by video, then instructor demonstration, followed by practice with feedback. | Low-fidelity phantom (LP Baby trainer (Simulab, Seattle, WA)) | Participants performance of infant lumbar puncture | 5-point Global Rating Scale | 2 | We suggest cautiously that the "invent and problem-solve, followed by instruction” approach was not inferior to the "Mastery Learning " approach, based on skill acquisition and "Preparation for Future Learning " assessment outcomes. With Mastery Learning anecdotally and empirically requiring more time, greater faculty involvement, and higher costs, our findings question the preference Mastery Learning has received relative to other instructional designs, especially in the healthcare simulation community. We encourage researchers to study the educational and resource impacts of instructional designs using non-inferiority designs. | N/A | 14 |
| Burgess 2011 | Implementation of a Lumbar Puncture (LP) simulation teaching module | 2011 | United States of America | Cohort study | Medical doctors | | Emergency medicine | 17 | 17 | N/A | Lumbar puncture | Participants initially completed a questionnaire to assess their lumbar puncture knowledge and performed a lumbar puncture simulation assessing procedural skills. Subsequently they participated in an lumbar puncture module lecture and simulation, emphasizing specific areas of deficiency. After the teaching module, they repeated the questionnaire and performed a second simulation. Seven months later they repeated the questionnaire and simulation | Lumbar puncture simulator (not elaborated) | Self-perceptive lumbar puncture skill level | 10-cm visual analogue scale | 1 | Resident education through a didactic and procedural module involving lumbar punctures can be used to enhance knowledge and skills of EM-1 residents, as well as maintain retention in the future. | N/A | 7,5 |
| Cheung 2019 | Why Content and Cognition Matter: Integrating Conceptual Knowledge to Support Simulation-Based Procedural Skills Transfer | 2019 | Canada | Randomized Controlled Trial | Medical students | | N/A | 66 | 66 | Not elaborated | Lumbar puncture | Participants had 25 min to review their assigned instructional video (Procedural Only, Integrated in Sequence, or Integrated for Causation).  All participants then attended a self-regulated simulation-based lumbar puncture training session to practice a simulated scenario of lumbar puncture on a part-task model (1 hour). During practice, participants had access to their instructional video (via laptop) and could alternate between practicing the scenario and reviewing the video.  After practice, participants were tested on the same scenario without access to the instructional video (post-test). One week later, participants returned for the follow-up session, requiring they complete a retention test, on the same scenario from the week prior, followed by a transfer test, on a new LP scenario. | Low-fidelity phantom (Lumbar Puncture Simulator II, Kyoto Kagaku Co., Ltd., Kyoto, Japan). | Different learning approaches impact on skills retention and transfer of lumbar puncture performance | Global rating scale | 2 | When teaching supports cognitive integration of how and why content, trainees are able to transfer learning to new problems because of their improved conceptual understanding. Instructional designs for procedural skills that integrate how and why content can help educators optimize what trainees learn from each repetition of practice. | N/A | 15,5 |
| Chuan 2015 | Design and validation of the Regional Anaesthesia Procedural Skills Assessment Tool. | 2015 | Australia | Cohort study | Medical doctors | | Anaesthesia | 9 | 9 | Clinical department | Neuraxial anaesthesia | A Delphi process was conducted to develop the checklist for regional anaesthesia procedures. Ten anaesthetists with expertise in regional anaesthesia were asked to score three scripted videos of nerve block performance (test) and after a one-month interval to re-score the same videos (retest) using the RAPS tool. | Patients | Design and validation of a regional anaesthesia procedural skills assessment tool | Combined checklist and global rating scale | 2 | The Regional Anaesthesia Procedural Skills tool used in this study is a valid and reliable assessment tool to score the performance of trainees for regional anaesthesia. | None | 13,5 |
| Ciccotto 2012 | Lumbar puncture teaching module standardization. Ensuring patient safety through proper training | 2012 | United States of America | Cohort study | Medical students | | N/A | 75 | 63 | Simulation centre | Lumbar puncture | Participants were presented to lumbar puncture procedure slide presentation followed by practical experience on lumbar puncture mannequins. | Low-fidelity phantom (not elaborated) | Participants confidence | Questionnaire (not elaborated) | 1 | SIMC training as the foundation of our teaching module had significant impact on procedure knowledge, proper technique, and comfort level in the group of medical students studied. A teaching module such as ours can be applied to most all procedural training and should be an integral component of medical student education and training | N/A | 8,5 |
| Cohen 2013 | Making july safer: Simulation-based mastery learning during intern boot camp | 2013 | United States of America | Cohort study | Medical doctors | | Internal medicine | 47 | 47 | Simulation centre | Lumbar puncture | The educational intervention consisted of three days of small group teaching sessions and individualized feedback and assessment. Interns rotated in groups of six through a lumbar puncture scenario. Teaching included both didactic content and the opportunity for simulation-based deliberate skills practice with individualized feedback. This was compared to interns from two prior training years who did not participate in the boot camp. | Low-fidelity phantom (Simulab Corporation, Seattle, Washington) | Performance of lumbar puncture in clinical skills examination compared to historical cohorts | 21-item checklist of procedural skills with a minimum passing score | 2 | A simulation-based mastery learning boot camp allows for individualized training, assessment, and documentation of competence before interns begin providing medical care. | N/A | 13,5 |
| Conroy 2010 | Competence and retention in performance of the lumbar puncture procedure in a task trainer model | 2010 | United States of America | Cohort study | Medical doctors | | Emergency medicine | 26 | 24 | N/A | Lumbar puncture | The residents viewed a 5-minute PowerPoint™ slide presentation covering technical aspects of the procedure, known complications, and complication rates. They also viewed a 15-minute video on performing the procedure. They then completed a baseline assessment of competency. After the baseline simulation-based assessment, the residents were given feedback on their performance by fourth-year residents or attending level emergency medicine physicians. The physicians giving feedback were instructed to use the critical actions checklist to guide the feedback given to the learners. The residents were then instructed to practice the procedure using the simulator. The residents self-recorded the number of practices attempts and, when they were confident that they could perform the steps of the procedure adequately, they notified one of the evaluators and performed a second procedure for postpractice assessment. Within 3-6 months a third test was performed to assess retention. | Low fidelity lumbar puncture phantom (NASCO, Fort Atkinson, WI) | To assess improvement and retention of procedural skills in lumbar puncture | Modified critical action checklist | 2 | This study demonstrated the achievement and retention of competency in the steps of the lumbar puncture procedure in a task trainer model | N/A | 14 |
| Corvetto 2017 | Validation of the imperial college surgical assessment device for spinal anesthesia | 2017 | Chile | Cohort study | Medical doctors | | Anesthesia | 30 | 30 | N/A | Spinal anesthesia (assessment tool) | Three groups of physicians with different levels of experience were video recorded performing a spinal anesthesia in a simulated lumbar puncture torso. Participants’ technical performance was assessed with ICSAD, a Global Rating Scale (GRS) and a specific Checklist. Differences between the 3 groups were determined by Kruskal-Wallis test with post hoc Dunn’s correction for multiple comparisons. | Low fidelity phantom (Gaumard® Lumbar puncture torso S411, Miami, USA) | To validate if The Imperial College Surgical Assessment Device could discriminate operators’ proficiency in spinal anesthesia | Global Rating Scale and a Checklist | 2 | This is the first validation study of ICSAD as an assessment tool for spinal anesthesia in a simulated model. Using ICSAD can discriminate proficiency between expert and novices and correlates with previously validated GRS. Its use in the assessment of spinal anesthesia proficiency provides complementary data to existing tools. Our results could be used to design future training programs with reliable goals to accomplish. | None | 13,5 |
| Crichlow 2017 | Integration of a simulation-based mastery learning lumbar puncture curriculum using observational learning into an emergency medicine intern boot camp | 2017 | United States of America | Cohort study | Medical doctors | | Emergency medicine | 14 | 14 | N/A | Lumbar puncture | The interns were divided into groups of 2 or 3 interns with 1 faculty facilitator and 1 lumbar puncture task trainer. While the first intern performed the lumbar puncture, the other(s) observed, and the faculty facilitator provided corrective feedback. When the attempt was completed, the interns alternated roles. Roles continued to alternate to allow for continued deliberate practice until the time limit for the session was reached. A post-test was then conducted individually with each intern to determine if they had achieved the minimum passing standard. | Low-fidelity phantom (not elaborated) | To evaluate if the minimum passing standard was achieved following simulation-based curriculum. | Checklist with a minimum passing score (not elaborated) | 2 | We have demonstrated that a simulation-based mastery learning lumbar puncture curriculum can be successfully integrated in a time-efficient and effective manner into an EM intern bootcamp curriculum by using structured group observational training. This study introduces new hypotheses for researchers to explore the impact of group training on procedural competency. | N/A | 11,5 |
| Dadoun 2015 | Peer teaching: An effective method for simulation-based instruction | 2015 | Canada | Randomized Controlled Trial | Medical students | | N/A | 62 | 62 | N/A | Lumbar puncture | 3 groups  Group 1 (control group) was initially taught via deliberate practice by an expert and then had 30 minutes of self-guided practice.   Group 2 (student teachers) were similarly taught by an expert and then had 30 minutes to teach a peer.   Group 3 (peer taught) was taught by group 2 and then given an additional 30 minutes of self-guided practice. All students had an equal amount of time with the task trainer  Assessments of students were conducted 4-6 weeks after training, with a previously validated 15-point checklist by a single blinded assessor. | Low-fidelity phantom (not elaborated) | Mean score on the 15-point checklist | 15-point checklist | 2 | The students taught by their peers achieved the same degree of competency as students taught by experts. This could potentially provide medical schools with valuable new resources for teaching. We did not observe a benefit to the learner from ‘teaching the skill’ to their peers, as compared to practicing on their own, though the study may have been underpowered to detect a small difference. Self-efficacy was no different between groups. | N/A | 13,5 |
| Drake 2015 | Defining competence in obstetric epidural anaesthesia for inexperienced trainees | 2015 | United Kingdom | Observation study | Medical doctors | | Anaesthesia | 105 | 81 | Clinical department | Epidural anaesthesia | Data from January 1996 to December 2011 from all obstetric epidurals by all trainees with no prior experience of obstetric an- aesthesia were extracted. Success rates were calculated. Midwife assessment of the quality of analgesia by the obstetric epidural throughout both labour and delivery as anything but ‘good’ was regarded as a ‘failure’. If there was any other additional analgesia hen the obstetric epidural was described as a ‘failure.’ CUSUM plots were generated with the acceptable success rate set at 65% and the unacceptable success rate set at 55% | Patients | Establishing a learning curve based on CUSUM for epidural anaesthesia | Midwife assessment. Failure: If spinal or general anaesthesia was used for operative delivery. Failure: If there was any other additional analgesia Failure: Complications  overall trainee success rate via CUSUM | 4 | CUSUM is useful for assessing trainee epidural competence. Trainees require approximately 50 attempts, as defined by CUSUM, to reach competence. | E.D. and J.C. – none declared. J.R.S. – has just left the BJA board. | 12 |
| Dugan 2023 | Assessing Best Practices in a Simulated Lumbar Puncture Workshop with Medical Students | 2023 | United States of America | Randomized Controlled Trial | Medical students | | N/A | 35 | N/A | N/A | Lumbar puncture | Participants were divided into groups: 1) Student-led, traditional method, 2) Student-led, Peyton’s, 3) Faculty-led, traditional, or 4) Faculty-led, Peyton’s. Groups had a participant: Teacher ratio of 2:1. Participants were taught how to perform a simulated lumbar puncture based on the specific teaching method before practicing one simulated lumbar puncture. Competence during independent simulated lumbar puncture attempts was assessed using binary checklist | N/A | Improvement in simulated lumbar puncture score between Halsted’s traditional “see one, do one, teach one” method and Peyton’s Four-Step Approach | Binary checklist | 2 | Contrary to prior research, we found Halsted’s traditional “see one, do one, teach one” method equally effective to Peyton’s Four-Step Approach for teaching simulated lumbar puncture workshops. Congruent with prior research, we found that student facilitators are equally effective as faculty facilitators when teaching simulated lumbar puncture workshops | N/A | 11,5 |
| Elhadi 2020 | Informed self-assessment versus preceptor evaluation: A comparative study of pediatric procedural skills acquisition of fifth year medical students | 2020 | Libya | Cohort study | Medical students | | N/A | 65 | 65 | N/A | Pediatric lumbar puncture | The course used a manikin and various clinical scenarios to simulate real-life cases. Each clinical scenario was first devised by a professor with two instructors; then, the students were allowed to voluntarily perform a certain skill under the supervision of the instructors, who provided feedback about any mistakes or misunderstandings. Participants completed informed self-assessment of their skills using a questionnaire. Self-assessment was defined as the involvement of participants in judging whether or not learning expectations were met or whether their ability to perform procedures were of an acceptable standard. The participants also rated their confidence in the procedure. Subsequently, their actual competency in each procedure was assessed based on preceptor evaluations using an objective structured clinical examination (OSCE) model. | Low- and medium fidelity phantom (not elaborated) | Improvement in procedural skill  Participants confidence | Objective structured clinical examination 4-point Likert Scale | 2 1 | High competence in several life-saving procedures was demonstrated among final-year medical students. The need for consistent and timely feedback, methods to increase medical students’ confidence, and further development and improvement of competency-based assessments are also highlighted. | N/A | 14 |
| Fischer 2017 | A simulation-based longitudinal procedural curriculum for pediatric residents improves self perceived competence | 2017 | United States of America | Cohort study | Medical doctors | | N/A | N/A | N/A | Clinical department | Lumbar puncture | Procedural workshops were held monthly during noon conference. Teaching methods included checklists, videos, knowledge assessments and hands-on simulation training. Self- perceived competence was assessed before and after each workshop using online surveys. | N/A | Self-perceived competence in lumbar puncture | Survey (not elaborated) | 1 | We present a feasible model of a longitudinal, simulation-based curriculum to improve resident self-perceived competence in procedures required by the Accreditation Council of Graduate Medical Education and others performed routinely during residency. Our next aim will be to investigate whether this improved competence leads to a greater number and quality of procedures performed on actual patients. | N/A | 7,5 |
| Friedman 2006 | Objective Assessment of Manual Skills and Proficiency in Performing Epidural Anesthesia-Video-Assisted Validation | 2006 | Canada | Cohort | Medical doctors | | Anesthesia | 6 | 6 | Clinical department | Epidural anesthesia | Over a 6-month period, the residents were videotaped three or four times when performing epidural catheter insertion. Each resident had early, middle, and late sessions videotaped during the rotation. Videos were evaluated by three blinded raters. | Patients | Validity and reliability of the assessment tool | Global-assessment scale and a 3-scale, 27-stage checklist | 4 | The results of our study show that scores on a system that consists of a global-rating form and a task-specific checklist had a significant relation to the number of epidural insertions performed (i.e., experi- ence). The interrater reliability of these assessment tools was very strong. Evaluation of technical skills by an objective tool under direct observation, as opposed to laboratory setting, may create a more reliable standard of assessment. Furthermore, residency programs could use these evaluations to identify deficiencies in teaching programs and trainees who require extra instruction. | N/A | 15 |
| Friedman 2009 | Clinical impact of epidural anesthesia simulation fidelity on short and long term learning curve | 2009 | Canada | Randomized Controlled Trial | Medical doctors | | Anaesthesia | 24 | 24 | Simulation centre and clinical department | Epidural anaesthesia | All participants attended didactic lectures on epidural catheter insertion, including anatomy, technical aspects, approach to common problems, complications, and contraindications.   The high-fidelity group spent 60 mins practicing epidural needle insertion on a simulator, that combines a physical interface with a virtual-reality display of needle progression. Each participant performed 4 insertions on the model, supervised by an experienced staff.   The low-fidelity group spent 60 minutes of supervised training on a greengrocer's model, which has been routinely used, and involves inserting an epidural needle into a banana. Each participant performed 4 insertions on the model, supervised by an experienced staff.   All participants then observed 5 procedures performed on patients and participated in an additional 5 procedures while supervised and helped by an attending anaesthesiologist.  Over a 6-month period, the subjects were videotaped 3 to 4 times, when performing independent epidural catheter insertion.  Each participant had baseline, middle, and late sessions videotaped during the rotation. | High-fidelity phantom (The Epidural Injection Simulator (Flinders MediTech Pty Ltd, Adelaide, Australia))  Low-fidelity phantom (Banana) | The effects of a high-fidelity epidural anaesthesia simulator on residents' ability to perform their first labor epidurals and on their learning, curve compared with a group having training with a low-fidelity model. | Global Rating Scale and Manual Skill Checklist | 4 | Our study shows that a simple model can be as useful for learning how to place an epidural catheter as an expensive anatomically correct simulator. New and more technologically advanced simulators should be compared against lower fidelity models to establish their utility and cost-effectiveness. | N/A | 16 |
| Gaies 2009 | Reforming procedural skills training for pediatric residents: A randomized, interventional trial | 2009 | United States of America | Randomized Controlled Trial | Medical doctors | | Paediatrics | 38 | 29 | N/A | Paediatric Lumbar puncture | The intervention group participated in a didactic session with faculty, observed the instructor performing the procedure, and then practiced the skills multiple times on a simulator under direct observation. Two days later both groups underwent a structured observational assessment on simulators. The control group learned procedures via traditional educational strategies (observation of more experienced clinicians) | Low-fidelity simulator (not elaborated) | Successful performance of the procedure on the simulator assessment at the end of the module. | Binary lumbar puncture checklist | 2 | Participants in the intervention group were more successful performing certain simulated procedures than controls when tested immediately after receiving the curriculum but demonstrated declining skills thereafter. Future efforts must emphasize retraining, and residents must have sufficient opportunities to practice skills learned in a formal curriculum. | None | 12,5 |
| Galen 2019 | A Curriculum for Lumbar Puncture Training in Internal Medicine Residency. | 2019 | United States of America | Cohort study | Medical doctors | | Internal medicine | 81 | 50 | N/A | Lumbar puncture | Residents prepared for the in-person training by watching the New England Journal of Medicine LP. The 1.5-hour group session (approximately 10 residents per session) began with a review of the questions in the post-video quiz, including a discussion of wrong answer choices. Next, an expert in the procedure demonstrated the lumbar puncture technique using the checklist and a commercially available mannequin. Each resident then performed a simulated lumbar puncture on the mannequin following the checklist with real-time feedback on his or her technique with peer and expert feedback. | Low-fidelity phantom (M43B Lumbar puncture simulator-II, Limbs & Things Ltd., Sussex Street, Bristol, UK) | Participants perception of usefulness of the curriculum.  Improvement in number of lumbar punctures performed on patients after completion of curriculum. | Survey  Log of number of lumbar punctures performed at graduation. | 1  3 | Despite not leading to an increase in LP rates, our curriculum was very well received by PGY-2 IM residents. | None | 12,5 |
| GANDHI 2023 | ULTRASOUND-ASSISTED LUMBAR PUNCTURE: A QUALITY IMPROVEMENT PROJECT | 2023 | United States of America | Cohort study | Medical doctors | | internal medicine and combined medicine-paediatrics | 67 | N/A | Simulation centre | Ultrasound-assisted lumbar puncture | Hands-on training and a didactic session on using ultrasound to assist in lumbar puncture in a simulated environment (not further elaborated).  Pre- and post-intervention surveys were performed. | N/A | Improvement in confidence of using ultrasound to assist in lumbar puncture | 4-point Likert Scale | 1 | Hands-on training and didactics in ultrasound-assisted lumbar puncture significantly increased the confidence in performing lumbar puncture and using ultrasound to assist in lumbar punctures. Creating a formalized curriculum and incorporating the same in residency training for internal medicine and combined medicine-paediatrics resident trainees will improve comfort and confidence in practicing this procedure. | None | 8,5 |
| Garrood 2010 | A structured course teaching junior doctor’s invasive medical procedures results in sustained improvements in self-reported confidence | 2010 | United Kingdom | Cohort study | Medical doctors | | N/A | 43 | 30 | N/A | Lumbar puncture | A one-day course was designed and implemented, providing small group training in lumbar puncture (LP). A brief talk outlining the indications, up-to-date guidelines, risks, issues of informed con- sent, technique and complications of each procedure were given before practical instruction in the technique. Attendees were asked to complete an anonymous questionnaire before, immediately after and three months after the course to rate their confidence in the skills taught on a scale of one (none) to 10 | Anatomical models (not further elaborated) | Confidence in lumbar puncture performance | 10-point Likert Scale | 1 | Significant improvements in self-reported confidence were seen for all procedures which were sustained at three months. Feedback was universally positive. Practical pre-clinical training may be a useful adjunct to patient-based training in invasive procedures. The course was particularly popular with foundation year trainees: ideally this training should be available before trainees’ first exposure in the clinical setting. | N/A | 8 |
| Gaubert 2021 | Positive effects of lumbar puncture simulation training for medical students in clinical practice | 2021 | France | Randomized controlled trial | Medical students | | N/A | 388 | 41 | Simulation centre and clinical department | Lumbar puncture | Simulation group: The training session’s theoretical objectives included the following items: 1) list the most frequent side effects and contraindications of LP; 2) list the precautions to be taken to prevent post-LP headache (PLPH); 3) diagnose and treat PLPH. The practical objective was to learn how to perform LP on the mannequin in sitting and lying position.  Control group:  Traditional “clinical training” at the hospital.  The first lumbar puncture performed by the student on a patient following the intervention, was evaluated in the study. | Low-fidelity phantom (Kyoto Kagaku) | 1) Improvement in procedural confidence  2) Success rate of first lumbar puncture in patients | 1. 10-point Likert scale (confidence pre/post)  2. The successful rate of the first LP performed by students. The need for technical assistance given by the supervisor  The successful collection of cerebro-spinal fluid (CSF), without any technical assistance from a supervisor | 1 4 | Simulation-based teaching was an effective way to improve students’ theoretical and practical knowledge. Whether this approach translates to other procedural skills in real clinical settings merits further study. The low participation rate in the study is due to the fact that students are not used to be included in educational studies and to the complexity of evaluation in routine clinical practice | None | 13 |
| Gerard 2013 | Validation of global rating scale and checklist instruments for the infant lumbar puncture procedure | 2013 | United States of America | Cohort study | Medical students and MDs | | Paediatric emergency medi-cine, neonatology and haematology attendings | 60 | 60 | Simulation centre | Infant lumbar puncture | Subjects were asked to ‘‘perform an LP on the simulator, demonstrating all of the steps that you would do on a real patient to properly perform the procedure.’’ After completion of all video recording sessions, 3 blinded, expert raters independently scored each subject’s video-recorded LP performance using the GRS tool. | Laerdal Baby strap neonatal task trainer (Laerdal, Wappinger Falls, NY) | To evaluate the validity and reliability of infant lumbar puncture tools in a simulated setting | Global Rating Scale (GRS) and 15-item checklist | 2 | This study provides some initial evidence to support the validity and reliability of the ILP-anchored GRS. Acceptable internal consistency was found for the checklist instrument. The GRS instrument outperformed the checklist in its discriminant ability and interrater agreement. | None | 13,5 |
| Goldman 2022 | A workplace procedure training cart to augment pediatric resident procedural learning | 2022 | United States of America | Cohort study | Medical doctors | | Paediatric | 24 | 13 | Clinical department | Paediatric Lumbar Puncture | A workplace procedure training cart was developed.  This allowed any resident at any stage of the procedural training trajectory to make use of the cart, either independently or with a preceptor.  The cart repurposed a standard bedside equipment mobile storage unit. To promote “learning” and “seeing,” a laptop computer was secured on top of the cart with links to several curated procedure videos. To facilitate “practice,” each labelled drawer stored SIM equipment needed for a given procedure along with a stocking checklist to ensure residents had all the necessary equipment to practice. To encourage residents to “prove” their skills, each drawer contained well-validated procedural competency assessment tools for the residents to use independently or for preceptors to use to standardize procedural coaching and formative feedback.   Furthermore, comparing resident participation in real patient procedures between the five months after cart implementation with the five months in the year prior. | Neonate simulator (not further elaborated) | 1) Perceived educational impact  2) Participation in clinical patient procedures after cart implementation compared to prior | 1) 5-point Likert scale  2) Clinical metrics (Electronic medical record) | 1 3 | A workplace procedural training cart was used mostly by paediatric interns. The cart cultivated residents' perceived confidence in real procedures but was not used by all residents or influenced residents' procedural behaviours in the pediatric emergency department. | N/A | 10,5 |
| Goldman 2022 | Formative Assessments Promote Procedural Learning and Engagement for Senior Pediatric Residents on Rotation in the Pediatric Emergency Department | 2022 | United States of America | Cohort study | Medical doctors | | Paediatrics | 35 | 31 | Clinical department | Low-fidelity Infant lumbar puncture phantom (not elaborated) | The resident completed the prequestionnaire prior to their scheduled informed consent OSCEs. OSCEs were completed from home via Zoom. Each OCSE took approximately 10 minutes per case (Week 1 and 2). Next, facilitators proctored procedural simulations during subsequent PED shifts. The procedural simulation took approximately 7.5 minutes to set up and complete. Upon completion of the procedural simulation, the resident immediately filled out the postquestionnaire and then received feedback from their facilitator (Week 2 and 3).  During the third week of the PED rotation, the lead facilitator reviewed all four videos and completed a Formative Feedback Report (FFR). | Simulation-based training (not elaborated). | 1) Residents’ confidences in the procedure  2) Number of lumbar punctures performed | 5-point Likert scale Clinical metrics (records) | 1 3 | Integrating feasible procedural formative assessments into the pediatric emergency department rotation had a positive impact on senior pediatric residents’ perceptions of their procedural knowledge, skills, confidence, and entrustment and was associated with increased procedural engagement. | None | 12,5 |
| Goolsby 2014 | Hybrid simulation improves medical student procedural confidence during EM clerkship | 2014 | United States of America | Cohort study | Medical students | | N/A | N/A | N/A | Simulation centre | Lumbar puncture | First, students completed a questionnaire at the beginning of the simulation lab assessing their confidence. Immediately after the simulation lab, they completed a second questionnaire. Finally, students completed the final questionnaire 3 weeks later at their final exam. In the morning session, students rotated through seven procedural trainers to teach students core EM skills. After the procedure training, the students each completed a patient scenario (septic from bacterial meningitis) | Low fidelity phantom (Kyoto Kagaku lumbar puncture simulator) | Confidence in performing lumbar puncture | 5-point Likert scale | 1 | Hybrid simulation training improves student confidence with procedural skills during an emergency medicine clerkship. | N/A | 7,5 |
| Grau 2003 | Ultrasound imaging improves learning curves in obstetric epidural anesthesia: A preliminary study | 2003 | Germany | Randomised controlled study | Medical doctors | | Anaesthesia | 10 | 10 | Clinical Department | Epidural anaesthesia with and without pre-procedural ultrasound | Two groups of residents performed their first 60 obstetric epidurals under supervision. One group used the conventional "LOR" technique. The other group proceeded in the same way but was supported by pre puncture ultrasound imaging. | Obstetric patients. | to evaluate the teaching possibilities of ultrasound imaging as a diagnostic approach to the epidural region n comparison to the rates of success obtained in a control group via learning curves | Success defined as:  Adequate obstetric epidural anaesthesia requiring a maximum of three attempts, reaching a VAS score of<1, while neither changing the anaesthesia technique, nor starting at another ver- tebral level. | 4 | Using ultrasound imaging for teaching epidural anaesthesia in obstetrics we found a higher rate of success during the first 60 attempts compared to conventional teaching. We believe this shows the possible value of ultrasound imaging for teaching and learning obstetric regional anaesthesia | N/A | 14 |
| Guasch 2010 | Monitoring skill acquisition in obstetric epidural puncture at a university hospital using the cumulative sum method | 2010 | Spain | Observational study | Medical doctors | | Anaesthesia | 9 | 9 | Clinical department | Epidural anaesthesia | The residents recorded their first 100 obstetric epidural analgesia procedures. This data was then analysed with the CUSUM method. | Patients | To develop and test an individualized tool for monitoring acquisition of technical skills based on the CUSUM approach | First-attempt success rate Need for help  CUSUM monitored | 4 | Although some third-year residents reach the targeted rate of success quickly, wer believe that close supervision is useful to control progress along the learning curve for individuals who need more help. | N/A | 10 |
| Guerra-Wallace 2010 | "Just-in-time" lumbar puncture simulation training for residents in the pediatric emergency department | 2010 | United States of America | Randomized Controlled Trial | Medical doctors | | N/A | 53 | 53 | Clinical department | Lumbar puncture in patients younger than 2 years old | Participants were randomized to undergo simulation training versus standard of care immediately prior to performing LPs on patients younger than two years old.  The intervention consisted of an educational video on LP preparation and performance, as well as direct practice on an infant LP simulator immediately prior to performance on the patient. | Infant lumbar puncture simulator (not elaborated) | Effectiveness of training intervention on improvement in clinical lumbar puncture success | Clinical metrics (patient chart review) | 4 | A randomized control trial of Just-in-time [ self-taught LP simulation for residents in the ED was feasible at our institution. A larger trial is needed to evaluate the effectiveness of Just in time [ LP simulation resident training. Our data found approximately 70% of residents complete the LP on the first attempt. To detect a 10% difference in LP success between groups (alpha 0.05, power 80%), an enrolment of 157 residents per arm would be needed. | N/A | 14 |
| Hale 2021 | Cohort study of hospitalists' procedural skills: Baseline competence and durability after simulation-based training | 2021 | United States of America | Cohort study | Medical doctors | | Internal medicine | 22 | 19 | Simulation centre | Lumbar puncture | Participants were videotaped performing lumbar puncture on a simulator to assess pretraining performance. Immediately after the baseline assessment, participants  received one-on-one, 60 min simulation-based training for each procedure by one of 3 instructors employing Peyton’s four-step model of procedure training as an educational framework. Training began with participants viewing a published lumbar puncture video. The instructor then demonstrated the procedure on the simulator, verbalising each of the steps as listed on the  checklist and in that specific order. The instructor then allowed the participant to practice on the simulator, while providing direct, specific feedback on the steps. The participants were allowed to practice as many times as they desired, either on particular steps or on the entire procedure from start to finish. In accordance with mastery learning practices once a participant reported that they had had sufficient practice, the instructor assessed their performance against the checklist. If they did not pass, they then iteratively received direct feedback and targeted practice on the steps that were not achieved until they were able to independently complete the entire procedure with achievement of the minimum passing score. A reassessment were conducted after 6 months. | Simulator (not elaborated) | Percentage of hospitalists obtaining passing scores on lumbar puncture  Self-reported procedural confidence | Minimum passing score checklist Survey | 2 1 | Hospitalists may be performing invasive bedside procedures without demonstration of adequate skill. A single evidence-based training intervention was insufficient to sustain skills for the majority of hospitalists over a short period of time. More stringent practices for certifying hospitalists who perform risky procedures are warranted, as well as mechanisms to support skill maintenance, such as periodic simulation-based training and assessment. | None | 13 |
| Henriksen 2017 | Assessment of Residents Readiness to Perform Lumbar Puncture: A Validation Study | 2017 | Denmark | Cohort study | Medical doctors and students | | Neurology, Internal Medicine and Emergency Medicine | 29 | 28 | Simulation centre | Lumbar puncture (assessment) | Development and validation of an assessment tool using clinician interviews and literature reviews. Study participants carried out the lumbar puncture procedure individually in a simulated setting and performances were video-recorded for assessment. | Low fidelity phantom Kyoto Kagaku Lumbar Puncture Simulator II (Kyoto, Japan) | To develop and collect validity evidence for an assessment tool for lumbar puncture performance including a standard to determine when trainees are ready for clinical practice. | Lumbar Puncture Assessment Tool (LumPAT) | 2 | We developed and demonstrated strong validity evidence for the lumbar puncture assessment tool. The tool can be used to assess readiness for practice | None | 13,5 |
| Huang 2023 | A mixed-reality stimulator for lumbar puncture training: a pilot study | 2023 | China | Cohort study | Medical doctors, students and nurses | | N/A | 40 | 40 | Simulation centre | Lumbar puncture | Participants received basic anatomy training of lumbar puncture, provided by instructors. After that, participants were guided to use the MR stimulator, including adjusting and testing HoloLens glasses, which provided a 3D image, to ensure comfort and accuracy of presentation. In the meantime, participants strengthened their understanding of spine anatomy through Glasses following the advice given by the trainer. The simulator used in the research was a common simulator without internal landmarks for lumbar puncture. Participant were asked to locate the L3-L4 interspace by palpation. The L3 and L4 spinous processes was correctly recognized, and the midpoint between the two spinous processes was used as the target of needle insertion. After the puncture space was accurately identified, the internal 3D image was projected on the simulator through the glasses, and the simulator with 3D image was presented on the computer screen at the same time. The trainees were allowed to understand the puncture position relative to the previously determined anatomical position.  The trainees were asked to practice needle inserting with anatomy in view through glasses. Once participants were confident with their skill, they were asked to insert a needle on simulator without anatomy in view. If the needle inserted correctly into the subarachnoid space, the fluid flew out of the needle, simulating the outflow of cerebrospinal fluid. | "Mixed Reality stimulator with HoloLens smart glasses (Microsoft) and a common simulator without internal landmarks for lumbar puncture." | Improvement in confidence | Survey (not elaborated) | 1 | The existing simulator was easy to be transformed into MR simulator. This study showed the usability and feasibility of MR simulator in lumbar puncture training. As a potentially good tool to simulated medical skills training, next, MR technology would be developed and evaluated in more clinical skills teaching scenarios | None | 8,5 |
| Iyer 2013 | Assessing the validity evidence of an objective structured assessment tool of technical skills for neonatal lumbar punctures | 2013 | United States of America | Cohort study | Medical doctors | | Paediatrics | 16 | 16 | N/A | Neonatal lumbar puncture (assessed) | Each resident was videotaped performing an LP on a simulation model. The six raters reviewed all of the videos (96 ratings). | Low-fidelity phantom (Laerdal Baby Stap, Laerdal Medical, Wappingers Falls, NY) | To develop an objective structured assessment of technical skills for neonatal LP (OSATS-LP) and to document validity evidence for the instrument | OSATS-LP rating instrument | 2 | The OSATS-LP has reasonable evidence in four of the five sources for test validity. This study serves as a launching point for using this tool in clinical environments such as the ED and, therefore, has the potential to provide real-time formative and summative feedback to improve resident skills and ultimately lead to improvements in patient care. | None | 13,5 |
| Katz 2017 | Teaching procedural skills to medical students: A pilot procedural skills lab | 2017 | United States of America | Cohort study | Medical students | | N/A | 180 | 106 | Cadaver laboratory | Lumbar puncture | [Intervention participants met 2 h once a week for 4 weeks. Students were asked to view NEJM procedural skills’ videos (http://www. nejm.org/multimedia/medical‑video) prior to laboratory sessions. Students were also provided with access to a commercially available spaced education program (QStream, Burlington, MA, USA). A single instructor supervised students individually as they practiced each procedural skill in 10 min intervals. Lumbar puncture was taught and practiced on cadavers.](http://www/) | Cadavers | Training interventions effect on confidence in performing a lumbar puncture | 4-point Likert scale | 1 | An innovative PSL may increase students’ confidence to perform procedural skills. Future studies will examine competency after a PSL. | None | 17 |
| KallidaikurichiSrinivasan 2018 | Proficiency-based progression training: an 'end to end' model for decreasing error applied to achievement of effective epidural analgesia during labour: a randomised control study | 2018 | Ireland | Randomized controlled trial | Medical doctors | | Anaesthesia | 17 | 14 | N/A | Lumbar epidural catheter | Participating trainees were randomly allocated to either group S (simulation training group) or group P (proficiency-based progression group).   During part one, trainees in both groups were given access to the same study material on labour epidural analgesia. An assessment test was done within 2 weeks of provision of the material to trainees in group  P only. Trainees in Group P were required to score a predefined pass percentage (80%) before they could proceed to the next phase of training. If the score was not met, additional time was given for the trainees to review the study material provided. No assessments were carried out at this stage of group S participants.  Part two comprised a standardised workshop (didactic session and simulation training session) run for each participant within 4weeks of receiving the study material. In group S, all participants received didactic teaching on the performance of labour epidural catheter placements (including all the metrics developed from the phase 1) followed by a simulation training session.The actual duration of simulator use was left to  the discretion of the trainees. No assessment was done at the end of their simulation training session.   In group P, all participants received didactic teaching, and the 74 metrics developed in phase 1 were described in detail using examples. A list of the metrics was provided to trainees in group P. Video recordings from phase 1 were used to illustrate how errors happen in ‘real-life’ clinical situations. Group P trainees received instruction on use of the same simulator. They were then required to practise, hands-on, each metric using the manikin. Focused feedback was given on how to avoid errors. Once the trainee had practised each metric, he or she demonstrated the procedure from the start to finish. Two assessors then used the study's validated assessment tool to independently score trainee performance on the simulator. Feedback on errors and critical errors identified during the procedure were provided. This process was repeated until the trainees attained a predetermined proficiency benchmark (as described earlier) on two consecutive procedures | Low-fidelity phantom (Manikin KKM43E, Cardiac services 2013, SISK Healthcare Group, UK) | Epidural failure rate | (i) accidental dural puncture; (ii) supervisor takeover  (iii) inadequate analgesia (presence of pain as perceived by the patient) | 4 | Procedure-specific metrics developed for labour epidural catheter placement discriminated the performance of experts and novices with an IRR of 0.88. Proficiency-based progression training resulted in a lower incidence of epidural failure compared to simulation only training. | None | 8,5 |
| Kessler 2011 | A randomized trial of simulation-based deliberate practice for infant lumbar puncture skills | 2011 | United States of America | Randomized Controlled Trial | Medical doctors | | Paediatric | 56 | 32 | Simulation centre | Infant lumbar puncture | A pre-test and questionnaire were collected. Individuals from both groups were then videotaped performing a lumbar puncture on a task trainer. Performance on this observed structured clinical examination (OSCE) was scored by the instructors, live, on the day of training, and later using video review by an author blinded to group assignment (M.T.) using a 15-item checklist of critical steps developed for this study.   Participants in both groups viewed an audiovisual presentation on the LP procedure. This 16-minute audiovisual presentation included the NEJM LP Procedure in Clinical Med-icine Video.  Participants in the intervention group then participated in a hands-on simulation-based coached deliberate practice training session in addition to the videos. This one-on- one session consisted of deliberate practice on the infant LP simulator while a faculty member coached them through the performance of 15 critical steps in the LP procedure. Trainees were required to master each step before moving on to the subsequent step, and therefore, the session was tailored to the individual learner based on their needs. Sessions were complete once the learner was ready and capable of demon-strating all critical steps flawlessly and independently from start to finish on the simulator. All trainees continued with repetitive deliberate practice until they could independently perform all tasks on the checklist. | Low fidelity phantom (Laerdal Baby Stap - Laerdal, Wappinger Falls, NY) | Improvement in clinical infant lumbar puncture skills  Improvement in procedural confidence | Self-reported clinical online data entry tool  4-point Likert scale | 4 1 | Participation in a simulation-based deliberate practice intervention can improve infant lumbar puncture skill. | None | 15 |
| Kessler 2013 | Interns' success with clinical procedures in infants after simulation training | 2013 | United States of America | Randomized Controlled Trial | Medical doctors | | Paediatric | 210 | 200 | Clinical department | Infant lumbar puncture | Participants viewed a 40-minute set of lumbar puncture videos published by New England Journal of Medicine and content developed by the study authors.  The simulation-based medical education mastery learning session occurred at the start of internship and used bench-top simulators and trained facilitators who guided participants in deliberate practice until they achieved a predefined level of mastery of the skill being taught. The training sessions continued until learners demonstrated independent mastery performance on the checklist (ie, sessions ranged from 20– 60 minutes depending on the learner) For 6 succeeding months participants reported clinical performance. | Low fidelity phantom (Laerdal Baby Stap ILP simulator (Laerdal Medical, Stockholm, Sweden)) | Improvement in clinical procedural success | Clinical metrics (an online self-report questionnaire for all ILP procedures on patients aged <365 days. LP success was defined as obtaining an adequate sample on the first attempt that had ,1000 red blood cells per high-power field on microscopic examination or that was described as clear) | 4 | Participation in a single simulation-based medical education mastery learning session was insufficient to affect paediatric interns’ subsequent procedural success. | None | 13,5 |
| Kessler 2017 | Screening residents for infant lumbar puncture readiness with just-in-time simulation-based assessments | 2017 | United States of America | Cohort study | Medical doctors | | Paediatric Emergency Medicine Combined track (depending on the hospital) | 1722 | 735 | Clinical department | Infant lumbar puncture | Participants completed a standardised published lumbar puncture curriculum composed of watching videos, demonstrating performance on a simulator and having individually coached practice sessions until a predefined mastery performance standard was achieved. A just-in-time readiness assessment would occur immediately prior to each resident’s first LP opportunity in the clinical setting. The resident was to perform a mock LP on a simulator while the supervisor observed and completed a global rating scale based on the resident’s performance. Residents who did not achieve passing score on the simulation assessment were instructed not to perform the clinical procedure.  Two groups were defined for comparison to our group that experienced the intervention as intended: (1) a historical group of residents from the 2 years prior (2010–2012) who with similar lumbar puncture training but no restriction from clinical performance by a threshold passing score; (2) the group of residents from the same 2 years (2012–2014) who performed a clinical lumbar puncture but did not participate in the intended readiness assessment (non-assessed participants). | Low-fidelity infant lumbar puncture phantom (BabyStap Laerdal Medical, Stavanger, Norway) | Improvement in procedural success following a just-in-time readiness assessment | Clinical metrics (Lab test) | 4 | Simulation-based readiness assessments performed in a point-of-care fashion were associated with several desirable behaviours but were not associated with greater clinical success with lumbar puncture. | None | 11 |
| Kilbane 2010 | Pediatric residents' ability to perform a lumbar puncture: Evaluation of an educational intervention | 2010 | United States of America | Cohort study | Medical doctors | | Paediatrics | 56 | 56 | Simulation centre Clinical department | Paediatric lumbar puncture | For the control group, clinical LP performance was monitored over a 6-month period during the final 6 months of their first year of training. For the experimental group, baseline LP knowledge and simulated LP performance were initially tested at the start of their first year of training. They then received a focused educational intervention. Six months after this intervention, the group repeated the LP knowledge and simulated LP assessment. Finally, these residents’ clinical LP performance was monitored during the final 6 months of their first year of training. | Low fidelity phantom (Laerdal Baby Stap neonatal task trainer (Laerdal, Wappingers Falls, NY)) | To evaluate the impact of an educational intervention on this skill in both a simulated and clinical environment. | Simulation assessment: 11-step dichotomous checklist  Clinical assessment, lumbar puncture success rate: CSF obtained by a study subject, regardless of number of attempts, with less than 5000 red blood cells and an uncontaminated CSF culture | 4 | After an educational intervention, paediatric first-year residents performed a simulated LP better than a group of second year residents who had greater clinical LP experience. The low number of clinical LPs performed limits our ability to determine the educational intervention’s impact in the clinical setting and reinforces the concern that recent changes to paediatric residencies may negatively impact residents’ procedural experience. | N/A | 15 |
| Konrad 1998 | Learning manual skills in anesthesiology: Is there a recommended number of cases for anesthetic procedures? | 1998 | Switzerland | Observational study | Medical doctors | | Anaesthesia | 11 | 11 | Clinical Department | Spinal and epidural anaesthesia | Eleven first-year residents were evaluated using a standardized self-evaluation questionnaire. The success or failure of the procedure to be tested was documented each day by the residents over an 1-yr period. | Patients | To investigate the learning process in anaesthesia, typical anaesthetic procedures were performed by inexperienced residents during their first year. | Success was defined as adequate technical performance - monitored using CUSUM.  When the procedure was successfully terminated without any physical help from a staff member, it was rated 1; when physical assistance by a staff  member was required, it was rated 0. The attending anaesthesiologist took over after a maximum of three attempts or an elapsed time period of 10 min for regional anaesthesia procedures or for arterial lines. | 4 | Learning curves are a valid tool for monitoring institutional and individual success. | N/A | 10 |
| Kopacz 1996 | The regional anesthesia learning curve: What is the minimum number of epidural and spinal blocks to reach consistency? | 1996 | United States of America | Observational Study | Medical doctors | | Anaesthesia | 7 | 7 | Clinical Department | Epidural and spinal blocks | Every regional anaesthetic technique attempted, and the degree of success were recorded over a 6 months period using a standard form. | Patients | To determine the minimum number of blocks a resident must perform to reach consistency during training in epidural and spinal blocks | Obtaining cerebrospinal fluid during attempted spinal anaesthesia Subsequent block during epidural placement | 4 | Approximately 20-25 procedures each are necessary before improvement in the techniques of spinal and epidural anaesthesia is demonstrated by residents in training. If a 90% success rate is desired, 45 and 60 attempts at spinal and epidural anaesthesia, respectively, may be necessary. | N/A | 12 |
| Krause 2016 | Utilizing a multimodal approach in teaching medical students ultrasound-guided procedures | 2016 | United States of America | Cohort study | Medical students | | N/A | 53 | 53 | N/A | Ultrasound-guided lumbar puncture | The training included fifteen-minutes of didactics and thirty-five minutes of hands-on training utilizing simulation manikins (not elaborated). The ultrasound-guided lumbar puncture was part of a multiple procedure curriculum. | Simulation manikin (not elaborated) | Confidence in procedure | 5-point Likert scale | 1 | This study suggests that teaching medical students with a fifty-minute session at a symposium can increase their confidence in performing US guided procedures both in the ability to perform the procedures and track the needle and in the technique of the procedures. This study supports the value of incorporating procedural training into undergraduate medical education. | N/A | 8,5 |
| Kulcsár 2013 | Preliminary evaluation of a virtual reality-based simulator for learning spinal anesthesia | 2013 | Ireland | Randomized Controlled Trial | Medical doctors | | N/A | 27 | 11 | Simulation centre | Dural puncture (lumbar puncture and spinal block) | Participants were randomly allocated to conventional or simulator-enhanced teaching groups. All participants received reading material in advance and a 40-minute standardized tutorial by one investigator in a PowerPoint presentation of the spinal anaesthesia technique. Participants were also shown a 10-minute video of an expert anaesthesiologist explaining and performing spinal anaesthesia’s Conventional Group had practical teaching of the equipment used, the positioning of themselves and the patient, the landmarks, and the direction of the needle. An orange was used to mimic the sensations to expect and the rate of advancement of the spinal needle. This took place in the form of an interactive discussion session. The Simulation Group received the same practical teaching, but instead of an orange, participants practiced on a simulator. The outcomes of the teaching were tested in two phases. Phase I: participants completed a written, multiple-choice questionnaire (MCQ) examination fol- lowed by simulator-based assessment Phase II was devoted to those participants for whom a suitable opportunity arose to perform spinal anaesthesia with supervision within three weeks of the teaching course. This phase consisted of structured observation of clinical performance of the procedure in the operating theatre environment. | VR-based simulator (a SenseGraphic Immersive workbench and a modified Phantom desktop with shutter glasses) An orange | Improvement in technical skills   Improvement in clinical spinal anaesthesia performance | Objective structured assessment of technical skills (OSATS) methods in simulation-based performance  Structured observation of clinical performance grading by an OSATS | 2 4 | Overall, no difference was measured between those taught with traditional methods and those, by a simulator-based program in regard to the performance of spinal anaesthesia. | N/A | 14 |
| Lean 2017 | End-task versus in-task feedback to increase procedural learning retention during spinal anaesthesia training of novices | 2017 | Singapore | Randomized Controlled Trial | Medical students | | N/A | 65 | 65 | N/A | Spinal anaesthesia | Upon learning from the instructional video, students were randomized to either IT or training with ET feedback. Prior to commencement of training, students were oriented to the equipment used for instruction, including the utility of the manikin as a simulation tool, the basic cleansing set and the spinal needles used. After a 5-min break, a blinded expert assessed the students using the checklist for spinal anaesthesia. A separate blinded expert performed a follow up retention test between 3 and 10 months after the initial test. | Epidural Anaesthesia Simulator trainer (life size unisex torso MW3 Kyoto Kagaku Co. Ltd.) | To investigate if continuous in-task (IT) or end-task feedback (ET) is more effective in teaching spinal anaesthesia to medical students. | Checklist | 2 | Both short-term and long-term learning retention of spinal anaesthesia ET feedback proved to be better (P\0.01) than IT feedback. The time taken for ET students was shorter at long-term testing. End-task feedback improves both short-term and long-term procedural learning retention. | N/A | 14,5 |
| Lenchus 2011 | A blended approach to invasive bedside procedural instruction | 2011 | United States of America | Cohort study | Medical doctors | | Internal Medicine | 85 | 76 | N/A | Lumbar puncture | Started with a 5 min course introduction. Then a written pre-test (10 min) was conducted, following by a skills check (15–20 min) – Those who had performed the indicated procedures on live patient prior to their arrival had their technical skills evaluated by using a procedural checklist. Those without prior experience in the performance of the procedure did not undergo the initial skills assessment, and therefore their data were not included in the final checklist analysis.  The training session began with a video instruction (The New England Journal of Medicine website (NEJM 2009) followed by a faculty-led discussion of the key topics. Faculty demonstration (approximately 30 min depending on procedure) – in the simulation laboratory. Individual practice (about 15 min depending on procedure) Procedural documentation (5 min). Post-written test. Clinical performance (variable) – subsequent to train-ing, participants formed the nucleus of a dedicated procedure service. Here, the participants operated under the direct supervision of an attending physician; the checklist used during the simulation-based instruction was also employed in the clinical area. | Low-fidelity phantom (Spinal injection simulator, item AB 1030, Armstrong Medical Industries, Inc. (Lincolnshire, IL)) | Improvement in technical lumbar puncture skills | Procedural checklist | 4 | A blended, standardized curriculum in invasive bedside procedural instruction can significantly improve performance in participants’ medical knowledge and technical skills. | None | 13,5 |
| Lenhard 2008 | An intervention to improve procedure education for internal medicine residents | 2008 | United States of America | Cohort study | Medical doctors | | Internal medicine | 39 | 39 | Clinical department | Lumbar puncture | The training was conducted over 2 weeks. On each of the first 5 days the residents received a 60-minute lecture created by the medical procedure service directors, viewed commercially available, instructional video recordings covering each procedure, and were taught how to use the procedure mannequins (Nasco, Modesto, CA, USA; Pacific Research Laboratories, Vashon, WA, USA). The first lecture discussed general aspects. The other lectures detailed each procedure. The faculty member would typically demonstrate the procedure on the mannequin and then observe the residents in its use until satisfied that they were correctly performing the manoeuvres; no fixed number of supervised repetitions on the mannequins were mandated.  After these educational activities, the team performed actual procedures as discussed. No formal assessment of residents’ knowledge was performed before performing supervised procedures on patients.  All medical procedure service activities involving the faculty physician took place in the afternoons; in the mornings the medical procedure service residents were strongly urged to practice repeatedly on the mannequins, and to review the lectures, video recordings, and textbook. No fixed number of unsupervised repetitions on the mannequins were mandated. They also used the mornings to each prepare a weekly 15- to 30-minute oral presentation on procedure related topics. The pre-medical procedure service survey was administered on the first day of the medical procedure service rotation before teaching occurred; the post-medical procedure service survey was administered on the final day of the rotation. Lumbar puncture was taught as part of a multiple procedure curriculum. | Mannequin (not elaborated) | Improvement in confidence | 5-point Likert scale | 1 | A comprehensive procedure service rotation of 2 weeks duration substantially improved residents’ comfort and self-perceived knowledge in performing invasive procedures. These benefits persisted at least to the end of the academic year. | None | 8,5 |
| Lew 2020 | Determining competence in performing obstetric combined spinal-epidural procedures in junior anesthesiology residents: results from a cumulative sum analysis | 2020 | Singapore | Observational study | Medical doctors | | Anaesthesia | 24 | 24 | Clinical department | combined spinal-epidural anaesthesia | Residents, with no prior experience performing epidurals, recorded all obstetric combined spinal-epidural procedures as a ‘success’ or ‘failure’, based on study criteria. Individual CUSUM graphs were plotted, with acceptable and unacceptable failure rates set at 20% and 35%, respectively. | Patients | to determine the minimum case experience required to demonstrate competence in performing obstetric combined spinal-epidural procedures using CUSUM | Success:   1) Procedure completed without physical assistance from another staff member  2. Number of attempts less than or equal to two; whereby each new skin puncture constitutes an attempt  3. Absence of inadvertent dura puncture, either witnessed or as suggested by clinical features  4. Absence of a repeat procedure for inadequate analgesia within four hours of initial administration  Monitored via CUSUM | 4 | Competence was achieved by 19/24 residents after the ACGME-required case experience of 40 combined spinal-epidural procedures, based on a predefined acceptable failure rate of 20%. In our experience, CUSUM analysis is useful in mon- itoring technical performance over time and should be included as an adjunct assessment method for determining procedural competence. | None | 10 |
| Lilamand 2023 | Lumbar puncture training with healthcare simulation improves self-confidence and practical skills of French medical residents in geriatrics | 2023 | France | Cohort study | Medical doctors | | Geriatric | 55 | 8 | Simulation centre | Lumbar puncture | Participants received a pre-training survey. Briefing and demonstration of LP performed on the simulator by the trainer (10 min).Training on lumbar puncture simulators with interactive feedback  (70 min). Each resident could perform at least one lumbar puncture using the four interchangeable lumbar puncture blocks, including at least two punctures under supervision by the session  trainer. Debriefing and interactive case report after training to share experience and point out the main issues related to lumbar puncture in older adults (10 min) A post-training survey was distributed immediately after the training. A follow-up survey was collected after 6 months post-training regarding clinical lumbar puncture performance. | Low-fidelity phantom (Kyoto Kagaku® reference KKM43E) | Improvement in skills level in clinical performance Improvement in confidence | Self-reported survey Self-reported survey | 1 4 | Residents were aware of the importance of mastering LP and requested additional training. Simulation may represent a major driver to improve their self-confidence and practical skills. | None | 10,5 |
| Lim 2016 | Low-Fidelity Haptic Simulation Versus Mental Imagery Training for Epidural Anesthesia Technical Achievement in Novice Anesthesiology Residents: A Randomized Comparative Study | 2016 | United States of America | Randomized Controlled Trial | Medical doctors | | Anaesthesia | 20 | 20 | N/A | Epidural anaesthesia | All participating residents received a didactic lecture on epidural anaesthesia, delivered by 1 of 2 board-certified anaesthesiologists with obstetric anaesthesiology subspecialty training. After the lecture, the residents were randomized into 2 groups. Group low-fidelity had 60 minutes of LF simulation training as a group (n = 10) for epidural catheter placement using a banana. Epidural catheter placement was attempted and supervised by 1 board-certified anaesthesiologist with obstetric anaesthesiology subspecialty training. Each resident progressed at his or her own pace until epidural catheter insertion, and adjustment was attained at least twice. Residents were permitted to interact with, and ask questions of, the instructing anaesthesiologist.  Group mental imaging had 60 minutes of scripted mental imaging training under the guidance of another board-certified anaesthesiologist with obstetric anesthe- siology subspecialty training. Residents were oriented to the parts of the epidural kit, epidural catheter placement was described stepwise in detail, and an anatomical spine model was used as a visual reference. No physical practice was performed. After 20 minutes of scripted guidance through mental rehearsal, the subjects engaged in a mental rehearsal for the task independently.     Immediately after the 60-minute group training sessions, residents from both groups individually performed epidural catheter placement on a high-fidelity, partial human task trainer for 3 consecutive trials on the same day, each under the direct observation of 1 of 7 skilled evaluators. | Banana | Improvement in epidural skill acquisition | A modified skills checklist | 2 | Mental Imagery is not different from low fidelity simulation training for epidural anaesthesia skill acquisition. Education in epidural anaesthesia with structured didactics and continual mental imagery training may suffice to prepare novice learners before an attempt on human subjects | None | 14,5 |
| Lydon 2019 | Can simulation-based education and precision teaching improve paediatric trainees' behavioural fluency in performing lumbar puncture? A pilot study | 2019 | Ireland | Cohort study | Medical doctors | | Paediatric | 39 | 38 | Clinical department | Infant lumbar puncture | Pre-test: Participants in the intervention group attended the simulation laboratory for baseline testing on the task trainer. Participants were told they would end the procedure following the writing-up of the procedure in the clinical note. Following these preparatory instructions, the participants were asked to carry out lumbar puncture ‘to the best of their ability’ and informed that the accuracy and duration of their performance would be assessed by one of the two trained observers.  Intervention: An overview of the intervention and the criterion for being deemed fluent was provided. Each performance of lumbar puncture was referred to as a frequency building trial. All trials were observed and timed by one of the two trained observers. The recording sheet required observers to provide a binary indication of whether each step was completed accurately or inaccurately. Upon conclusion of the trial, the observer provided the participant with corrective feedback.   Two post-tests were conducted following intervention.The retention post-test was conducted in the same manner as baseline testing but took place a minimum of one month post-intervention. The stability of behaviour was assessed by examining performance of lumbar puncture in the presence of distraction. Therefore, we utilised a series of interrupting questions that were not related to the ongoing task.  Comparator group testing: The 10 paediatric registrars in comparator group A visited the simulation laboratory individually. They were asked to perform lumbar puncture in test conditions equivalent to those used for baseline testing in the inter- vention group. Accuracy and duration of performance were recorded.  Audit period: A retrospective chart audit was conducted of the charts of patients under 12 months who had undergone lumbar puncture in the 12 month period beginning July 2016. For each chart, the individual responsible for carrying out the procedure was recorded (i.e., intervention group participant or paediatric registrar/specialist registrar/consultant) along with the lumbar puncture outcome. | Low-fidelity phantom (LumbarPuncture Baby, Simulab) | Improvement in behavioural fluency in infant lumbar puncture skills   Success of lumbar puncture performance in a clinical setting | Checklist  Clinical metrics (from audit) | 2 4 | The programme of simulation-based education with frequency building and precision teaching delivered produced behavioural fluency in lumbar puncture among paediatric trainees. Following the intervention, the performance of these participants was equivalent to, or greater than, that of senior paediatricians. This study supports the need for further research exploring the effectiveness of simulation-based education with precision teaching to train procedural skills to fluency, and the consideration of how best to explore the impact of these on patient outcomes. | None | 14 |
| McMillan 2016 | Lumbar puncture simulation in pediatric residency training: Improving procedural competence and decreasing anxiety | 2016 | Canada | Cohort study | Medical doctors | | Paediatric | 20 | 16 | N/A | Infant lumbar puncture | Participants completed a pre-test questionnaire including baseline information and were asked to complete the State-Trait Anxiety Inventory - State Anxiety Scale immediately prior to entering the room to perform the lumbar puncture. Immediately after completing the STAI-S, residents entered a room used for objective structured clinical examinations (OSCEs) on a task trainer. The performance of each resident was scored by direct observation. Within 1 month of completing the pre-test, the paediatric residents participated in an interactive teaching session. The session included a series of short videos demonstrating an lumbar puncture performed by an attending physician. Residents were also provided with an opportunity to practice lumbar puncture proficiency in small groups, using the paediatric lumbar puncture simulator, and to assemble the components of the lumbar puncture test kit (i.e. manometer to check opening pressure, opening tubes, etc.) under the supervision of an experienced neurologist.Residents were then scheduled to complete a post-test (i.e. repeat STAI-S and observed LP) within 4 months of the teaching session. | Low-fidelity phantom (Pediatric Lumbar Puncture Simulator - # KKM43C; Limbs and Things, Savannah, GA) | Changes in procedural lumbar puncture skills Changes in self-reported anxiety | 21-item lumbar puncture skills checklistState-Trait Anxiety Inventory - State Anxiety Scale | 21 | Lumbar puncture simulation training combined with an interactive training session may be a useful tool for improving procedural competence and decreasing anxiety levels, particularly among those at an earlier stage of residency training. | None | 12 |
| Meerkov 2017 | A simulation-based procedural curriculum for pediatric interns improves self-perceived competence | 2017 | United States of America | Cohort study | Medical doctors | | Paediatric | N/A | N/A | N/A | Lumbar puncture | Teaching methods included checklists, videos, knowledge assessments and simulation (not elaborated). Self-perceived procedural competence was assessed using a blinded survey completed immediately before and after the curriculum. Lumbar puncture was part of a multiple procedural curriculum. | Simulation training (not elaborated) | Self-perceived competence in lumbar puncture performance  Improvement in amount of clinical procedures performed | Survey Self-reported survey | 1 3 | Implementation of a simulation-based procedural curriculum at the onset of internship improved self-perceived procedural competence for almost all procedures. We aim to assess competence at the end of the 2016 intern year and continue longitudinal surveillance of procedures performed to clarify the impact of this curriculum and determine skill and confidence retention. | N/A | 6,5 |
| Melamed 2017 | Sim one, teach one-senior resident-led pediatric intern procedural training | 2017 | United States of America | Cohort study | Medical doctors | | Paediatric | 34 | 34 | N/A | Lumbar puncture | Interns received 4, 2-hour, senior resident-led simulation-based procedural training sessions. Interns’ procedural performance was videotaped and assessed by trained, blinded raters. Skills, confidence and knowledge were compared pre- and post-training. Lumbar puncture training was part of a multiple-procedural curriculum. | Simulation based training (not elaborated) | Improvement in procedural skills Improvement in procedural confidence | N/A Survey (not elaborated) | 2 1 | Senior paediatric resident-led simulation-based procedural training significantly improved paediatric interns’ procedural skill, knowledge and confidence, and enhanced senior residents’ confidence performing and teaching paediatric procedures. Simulation-based procedural trained interns outperformed 2nd year non-simulation-based procedural trained residents. Senior paediatric resident-led simulation-based procedural training is a feasible addition to paediatric procedural training and can easily be replicated using readily available resources. | N/A | 11,5 |
| Mohamed 2021 | Proficiency-based progression training: implementing a novel approach to training for epidural analgesia in labour | 2021 | Ireland | Cohort study | Medical doctors | | Anaesthesia | 12 | 12 | Clinical department | Epidural anaesthesia | The training course comprised two parts. During par tone, trainees had access to the pre‐course study materials at their convenience. Each participant undertook A theoretical test independently and was required to pass before moving on to the next training part. If the pass score was not achieved, additional time was given for the trainees to review the study material and another set of questions were provided.  Part two comprised a standardised workshop (teaching and simulation training session) which commenced with a trainer providing didactic teaching, including detailed descriptions of each pre‐defined metric. When both trainer and trainee were satisfied with the trainee’s understanding of each metric, a simulation training session was delivered. Participating trainees were instructed on the use of an epidural simulator in the presence of, and with advice from, the trainer. Then, each trainee was required to practise, hands‐on, each metric using the manikin. Once the trainee had demonstrated proficiency in each stage of the procedure, as judged by the trainer, and additionally had an opportunity to practise each metric to their satisfaction, they were invited to perform a “start to finish” manikin demonstration of the procedure without interruptions. This demonstration took place without input or guidance from the supervisor and was videotaped.   Two trained assessors experienced in PbP assessment who met the criteria for expert trainer, and who were unaware of the trainee’s identity, independently scored trainee performance on the simulator from the recorded videos.  Each participating trainee proceeded within two weeks of successfully completing the PbP course to perform clinical procedures in the labour ward. The first 15 epidural insertions by each trainee were followed up. Upon completion of each attempt, trainees documented the nature of supervision, and specified any particular difficulty or adverse event encountered | Low-fidelity epidural phantom (Manikin KKM43E, Cardiac services 2013, SISK Healthcare Group, UK) | Clinical feasibility of proficiency-based progression training in epidural insertion | Clinical metrics | 4 | In our experience, PbP training in epidural placement is feasible within existing departmental resources in a busy tertiary teaching hospital setting. | None | 15 |
| Mourad 2012 | A randomized controlled trial of the impact of a teaching procedure service on the training of internal medicine residents. | 2012 | United States of America | Randomized Controlled Trial | Medical doctors | | Internal Medicine | 45 | 44 | Simulation centre | Lumbar puncture | All interns received the standard procedure curricular instruction: a half-day simulation course dedicated to providing hands-on instruction with ultrasound technique, central venous catheter insertion, thoracentesis, and lumbar puncture performance. In addition, the intervention group participated in 10 didactic sessions that included procedure safety, informed consent, infection control and documentation practices, ultrasound basics, and in-depth, procedure-based lectures reviewing indications and contraindications, procedure preparation, anatomy, technique, troubleshooting, management of complications, and follow-up studies and their interpretation. These sessions were supplemented with independent readings and 3 evidence-based, interactive computer modules reviewing best practices for each procedure. Each intern was required to demonstrate procedure technique using step-by-step hands-on simulation with the procedure kit before performance in a clinical setting.  Lumbar puncture training was part of a multiple-procedure curriculum. | Simulation (not elaborated) | Improvement in autonomy of clinical procedures Improvement in procedural confidence | Clinical metrics 5-point Likert scale | 3 1 | A 2-week hospitalist-supervised procedure service rotation substantially improved residents’ experience, confidence, and knowledge in performing bedside procedures early in their training, with this effect sustained through the PGY-2 year. Standardized procedure service rotations are a viable solution for programs seeking to improve their procedure-based education. | None | 12 |
| Mousavi 2023 | Effects of a Novel Blended Virtual Reality and Clinical Learning Environment on the Learning Transfer of Anesthesiology Residents | 2023 | Iran | Randomized Controlled Trial | Medical doctors | | Anaesthesiology | 25 | 25 | Simulation centre and clinical department | Spinal anaesthesia | Participants were randomized to blended virtual reality/clinical environment training or only clinical environment training.   The blended virtual reality/clinical environment training was designed as followed: 1) video presentation of the spinal anaesthesia procedure through VR, complementary explanations by the  professor, and group questions and answers; 2) performance of the procedure on a virtual patient by the resident in the VR laboratory and corrective feedback from both the professor and the software; 3) performance of the procedure by the professor on a real patient in the operating room, with the resident observing the procedure, followed by group questions and answers; and 4) participation of the anaesthesiologist resident in the procedure on a real patient under the professor's supervision in the operating room and receiving corrective feedback. The total implementation time of the program was four weeks.   Clinical environment: The four training stages were completed over four weeks on real patients in the operating room; the residents' roles changed depending on their ability to  observe, participate, and perform independently. In this group, the residents were not allowed to perform the procedure independently until the professor’s approval due to patient safety risks. | Virtual reality patient (not elaborated) and Patients undergoing surgery requiring spinal anaesthesia | Improvement in learning transfer | A 360-degree assessment tool based on a five-point Likert scale | 4 | The blended virtual reality/clinical learning environment was more effective than clinical learning environment in improving residents’ learning transfer. Besides, an increase in scores indicated an improvement in professional competence. | N/A | 16 |
| Munoz-Leija 2024 | Development and Evaluation of An In-House Lumbar Puncture Simulator for First-Year Resident Lumbar Puncture Procedure Learning. | 2024 | Mexico | Randomized Control Study | Medical doctors | | Anaesthesiology | 11 | 11 | N/A | Lumbar puncture | Initially, both groups did a simulation of lumbar puncture with the simulator to obtain a basal measurement of their knowledge of the procedure and confidence in its performance. A baseline perceived confidence was measured with an online-based Likert-type survey.   Afterward, the residents attended a class given by experts belonging to the anaesthesiology service, where the procedure to perform an lumbar puncture was thoroughly explained, based on our adapted lumbar puncture requirements checklist. The control group continued with the traditional method (i.e., attending further classes and performing lumbar punctures under expert supervision), while the experimental group complemented their training with the continuous use of the simulator until they achieved at least 30 simulated procedures for 3 months.   Perceived confidence was measured again after 3 and 6 months.   Eighteen months after the intervention, the clinical performance of both groups was assessed by evaluating specific criteria in 10 patients from each group. The criteria evaluated included procedure time (from the start of local anaesthesia administration), the number of attempts made, and whether a redirection was needed. | Lumbar puncture simulator created for this project (3D printing and silicone casting, based on a lumbar spine CT) | Improvement in lumbar puncture performance Confidence in lumbar puncture skills | Clinical metrics 5-point Likert scale | 4 1 | Our study demonstrates that an in-house LP simulator is an effective and practical approach for first-year anaesthesiology residents to learn the LP procedure. This approach could be particularly useful in settings with limited resources and a lack of sufficient patients to practice on, as it provides an opportunity for faster learning and increased self-confidence. | None | 14 |
| Naik 2003 | Cusum analysis is a useful tool to assess resident proficiency at insertion of labour epidurals | 2003 | Canada | Observational study | Medical doctors | | Anaesthesia | 13 | 11 | Clinical department | epidural anaesthesia | Residents unfamiliar with epidural anaesthesia kept a log of their labour epidural successes and failures during a six-month hospital rotation. Failure was defined as a dural puncture or relinquishing the procedure to staff. Cusum analysis was performed using an acceptable failure rate of 10%. | Patients | to determine the number of epidural attempts required to attain proficiency over the six-month training period. | Successful epidural was an independently placed epidural catheter that provided some degree of analgesia, without physical assistance from a staff anaesthesiologist.  Monitored using CUSUM | 4 | After a period of training, residents are expected to perform the skill of labour epidural insertion independently. This study illustrates that some residents may need as many as 75 attempts to ensure proficiency. Training programs could use cusum to track the progress of their residents’ technical skills in order to guarantee an adequate experience. | N/A | 10 |
| Ocel 2006 | Formal procedural skills training using a fresh frozen cadaver model: A pilot study | 2006 | United States of America | Cohort study | Medical students | | N/A | 7 | 7 | Procedural Skills laboratory (cadaveric specimens) | Lumbar puncture | The students rotated around the stations, attempting the procedure on the mannequin before the cadaver.  Two physician educators supervised and provided feedback to students throughout the course.  In the mannequin and cadaver models success in lumbar puncture procedure, backflow of clear fluid indicated success.  A pre-procedure questionnaire documented students’ prior experience with each procedure. A post procedure questionnaire asked students to compare the mannequin and cadaver model for each procedure in terms of preference and perception of realism.  Lumbar puncture was part of a multi-procedural curriculum. | Cadaver Mannequin (not elaborated) | Improvement in lumbar puncture confidence | 5-pint Likert scale | 1 | The pilot course improved the overall confidence of the students in performing basic skills. In addition, despite the fact that the mannequin was somewhat easier to perform a number of procedures on, the fresh frozen cadaver was a more realistic model and the preferred model for practicing the skills. The fresh frozen cadaver is a feasible and valid instructional tool for training procedural skills and has the advantage of being more realistic than a typical mannequin model. | N/A | 7,5 |
| Oxentenko 2003 | A multidimensional workshop using human cadavers to teach bedside procedures | 2003 | United States of America | Cohort study | Medical doctors | | Internal Medicine | 143 | 143 | Simulation centre and cadaver laboratory | Lumbar puncture | The workshop was conducted over a 2-year period. The workshop was conducted 1 week before the start of their 1st year of residency and covered thoracentesis, paracentesis, lumbar puncture, arthrocentesis, and bone marrow biopsy. Two groups were simultaneously engaged in either a large-group didactic lecture with demonstrations or in small- group, hands-on experience with the cadavers. Staff and subspecialty fellows delivered the large-group didactic lectures. As each procedure was being reviewed and demonstrated by the discussant on a single cadaver model, a direct view and projected overhead display of the procedure were visible for residents to follow.  The hands-on experiences occurred in small groups of three to four residents per cadaver. Senior-level internal medicine residents and staff supervised the cadaver stations and answered questions. During each station, the residents were required to demonstrate proper technique for the procedure.  Lumbar puncture was part of a multi-procedural curriculum. | Cadaver | Improvement in procedural confidence Demonstration of skills required to safely complete a lumbar puncture | Likert scale survey (not elaborated) Procedural checklist (not elaborated) | 1 2 | Our multidimensional educational intervention may be an effective tool for improving the knowledge, skills, and attitudes required for performing bedside procedures. More research needs to be done to determine if this workshop increases the number and proficiency of bedside diagnostic procedures performed by residents. | N/A | 9 |
| Parlar-Chun 2019 | The effect of lumbar puncture simulations on pediatric residents | 2019 | United States of America | Cohort study | Medical doctors | | Paediatric | N/A | 105 | N/A | Paediatric Lumbar Puncture | Paediatric residents were surveyed at the end of each month regarding their confidence, success, need for multiple attempts, use of local anaesthetic, and use of stylet out technique Pre-intervention period was from July 2017 to December 2017. From January 2018 to June 2018, paediatric residents received monthly lumbar puncture simulation sessions at the beginning of the month that included a review of a lumbar puncture procedural checklist, viewing of POISE Paediatric Lumbar Puncture instructional video, and hands on practice on lumbar puncture simulation modules. | Simulation module (not elaborated) | Improvement in lumbar puncture success rate Improvement in lumbar puncture confidence | Clinical metrics survey Responses (not elaborated) | 4 1 | Monthly lumbar puncture simulations are not enough to increase LP success rates. However, simulation may be useful in increasing knowledge, experience, and confidence in the procedure. | N/A | 11,5 |
| Patel 2008 | Training effect of skills courses on confidence of junior doctors performing clinical procedures | 2008 | New Zealand | Cohort study | Medical doctors | | N/A | 33 | 32 | Advanced Clinical Skills Centre | Lumbar puncture | The course involved a video demonstration of each clinical procedure by senior clinicians of the appropriate specialty. This is followed by supervised practical workshops using a range of models specific to the procedure (not further elaborated). Surveys was distributed pre-course, post-course, and at 5 months' follow up.  Lumbar puncture was part of multi-procedural curriculum. | Simulation models (not elaborated) | Improvement in procedural confidence | 5-point Likert scale | 1 | The procedural skills course produced a significant increase in confidence in the short term, but this decreased unless there was ongoing clinical experience with the procedure. The benefit of short courses is eroded by the lack of reinforcement through continuing experience. | None | 9,5 |
| Reinhardt 2012 | Intern self-assessment and prediction of lumbar puncture success | 2012 | USA | Cohort | Medical doctors | | Paediatrics Emergency Medicine | N/A | N/A | N/A | Infant lumbar puncture | Mastery training with a video and then simulation training. A just-in-time training was performed. A questionnaire including a validated global skills assessment was completed by the intern/supervisors after the JIT and lumbar puncture. | Simulation (not further elaborated) | To determine the agreement between self-assessment and supervisor assessment of ILP performance | Global skills assessment instrument | 4 | The success rate of lumbar puncture performed by paediatric interns was low with a concordance between the paediatric interns’ self-assessment of their lumbar puncture skills and the success rate of the lumbar puncture. Despite similarly low levels of self-assessment of their lumbar puncture skills, higher lumbar puncture success rate was observed among emergency medicine interns. Supervising physicians’ ability to predict successful lumbar puncture by interns was low. | N/A | 13 |
| Restrepo 2015 | Ability of pediatric emergency medicine physicians to identify anatomic landmarks with the assistance of ultrasound prior to lumbar puncture in a simulated obese model | 2015 | United States of America | Cohort study | Medical doctors | | Paediatric emergency medicine | 19 | 19 | N/A | Ultrasound-assisted lumbar puncture | The educational intervention then began with a 1-hour lecture on ultrasound fundamentals. This was followed by 30 minutes of hands-on ultrasound training that included basic machine use, image archiving, transducer selection, and scanning techniques on a simulated vein block and LP mode. After completion of proctored scanning, each individual was then tested in lumbar puncture on the obese LP model. The participant was responsible for obtaining and saving ultrasound images in both the longitudinal and transverse planes. After images were obtained, the participants marked the lumbar model to indicate the planned needle insertion site, which should correlate to the interspinous space. The timing was started when the participant picked up the US probe to begin scanning and was completed when the participant finished marking the model. The same person documented successful fluid aspiration, which was the defining factor for deeming an LP attempt successful. This process was repeated 3 times by each participant, and then a postintervention questionnaire was completed. Two board-certified emergency medicine physicians with specialized ultrasound training independently reviewed the archived images obtained for adequacy. | Low-fidelity phantom (Blue Phantom, Redmond, Wash) | Improvement in confidence of the procedure | 5-point Likert scale | 1 | After a brief education intervention, paediatric emergency medicine physicians with little to no previous training in ultrasound can obtain adequate lumbar anatomic images and successfully perform lumbar puncture in a simulated obese model. Comfort level with ultrasound significantly improves with a short course in ultrasound fundamentals. | None | 9,5 |
| Roehr 2021 | The Feasibility of Virtual Reality and Student-Led Simulation Training as Methods of Lumbar Puncture Instruction | 2021 | United States of America | Randomized Controlled Trial | Medical students | | N/A | 25 | 25 | N/A | Lumbar puncture | All participants completed a 15-min orientation led by an emergency medicine physician explaining the lumbar puncture procedure, then watched a 2-min video created by the Student Led Independent Procedure Simulations (SLIPS). All students were then moved into a separate testing room and were individually recorded performing a pre-intervention lumbar puncture.  After baseline testing, students were randomly divided into the VR training group or the SLIPS training group.  Students in the standard SLIPS training group were taken to a procedural room and were shown the instructional lumbar puncture video from the original orientation again. They were subsequently subdivided into smaller groups with 1–3 students each and were given 45 min to practice on a task trainer together with SLIPS leaders providing hands-on instruction and feedback in real time at each station.  Students in the VR group were taken to a virtual reality training room. These students were subdivided into smaller groups of 1–3 students each and were given 45 min to practice performing a lumbar puncture on a VR trainer. | Low-fidelity lumbar puncture phantom (Simulab; Washington) VR lumbar puncture training module using Arivis InViewR (AG Imaging Science Unit, Phoenix, AZ) | Improvement in lumbar puncture skills Improvement in procedural confidence | Critical action checklist and time needed to complete the procedure  Survey (not elaborated) | 2 1 | Both virtual reality and student-led simulation training were useful training modalities, with hands-on simulation showing better results versus virtual reality training in this setting. | None | 14,5 |
| Sattler 2020 | Simulation-Based Medical Education Improves Procedural Confidence in Core Invasive Procedures for Military Internal Medicine Residents. | 2020 | United States of America | Cohort study | Medical doctors | | Internal medicine | 49 | 36 | Simulation centre | Lumbar puncture | The course included multiple two-hour sessions that were conducted over two separate days with each session including three of six procedural skills: lumbar puncture, arterial line, central line, thoracentesis, paracentesis and arthrocentesis. Participants were divided into small groups comprised of two or three residents that rotated through each skill station. Prior to simulation sessions, participants completed a survey on confidence and repeated the same survey after the session.  Lumbar puncture was part of a multi-procedure curriculum. | N/A | Improvement in confidence in procedure | 5-point Likert Scale | 1 | Internal medicine residents across all post-graduate year (PGY) levels at our institution lacked confidence to independently perform core internal medicine procedures. Utilizing simulation-based medical education as an adjunct to clinical training is well accepted by internal medicine trainees and resulted in significantly improved procedural confidence. This intervention was well received by trainees and could feasibly be replicated at other active-duty military internal medicine residency programs to assist with readiness. Research is currently in progress to correlate in-situ competency and evaluate clinical outcomes of this improved confidence. | None | 8 |
| Shaikh 2021 | Feasibility of ultrasound-assisted lumbar punctures performed by pediatric oncologists at the point of care. | 2021 | Canada | Cohort study | Medical doctors | | Paediatric oncologists | 3 | 3 | N/A | Ultrasound-assisted paediatric lumbar puncture | The study was separated into a Training phase and a Clinical phase. During the Training phase, three paediatric oncologists with no prior ultrasound training completed a structured curriculum. The learners began by reviewing written and online educational materials. One week later, they attended a lecture and introductory session on ultrasound. Then, the learners attended three hands-on simulation workshops where they performed ultrasound scanning on live models (young adult standardized patients).  During the Clinical phase, ultrasound-assisted LPs were performed by one of the three paediatric oncologists during routinely scheduled lumbar punctures for children with cancer.  All children had deep sedation or general anaesthesia induced by an anaesthesiologist and were positioned in the left lateral decubitus. Once sedated, ultrasound scanning was performed by one of the three paediatric oncologists. The point of insertion was marked by an adjacent marker or by a skin indent. The ultrasound gel was thoroughly removed, and the skin was cleaned and sterilized as normal. The lumbar puncture was performed following the ultrasound assessment. | Live paediatric models (1-18 years) | Procedural performance in patients in at least 30 patients within 6 months  Usefulness of procedure following a structured curriculum | Clinical Metrics   5-point Likert scale | 4 1 | Pediatric oncologists readily achieved competence in ultrasound-assisted lumbar punctures, and ultrasound was commonly perceived as helpful. It is feasible to proceed to a randomized trial of this procedure in pediatric cancer. | None | 11 |
| Shammari 2018 | Evaluation of effectiveness of a paediatric simulation course in procedural skills for paediatric residents — A pilot study | 2018 | Saudi Arabia | Cohort study | Medical doctors | | Paediatric | 16 | 13 | Simulation centre | Lumbar puncture | The course was conducted in two sessions, scheduled one week apart, offering a total of eight skills as given below. During the first session, the residents were trained in four techniques, namely: 1) lumbar puncture and cerebrospinal fluid (CSF) interpretation, 2) oral intubation, 3) bone marrow aspiration, and 4) critical airway management. During the second session,  conducted a week later, they were trained for the remaining 4 techniques: 1) chest tube insertion, 2) pleural tap, 3) insertion of central venous pressure line, and 4) arthrocentesis. The residents were divided into 4 groups. The skills were set in a series of 4 stations  Each group was assigned to start at a given station and rotate to the remaining stations in sequence, spending a uniformly fixed time period. All the residents were exposed to all the techniques at the stations and had ample time to practise the technique at the respective station, where the technique was demonstrated and then supervised by a consultant paediatrician/clinical skills team member. For each skill/task, a structured checklist was prepared and provided to all trainees for standardised training.   Lumbar puncture was part of a multi-procedural curriculum. | N/A | Improvement in procedural confidence Improvement in procedural skills | 5-point Likert scale Ojective Structured Clinical Examination (OSCE) | 1 2 | Simulation course was significantly successful in improving residents' clinical skills and confidence in performing critical tasks. | None | 13 |
| Stolarek 2007 | Procedural and examination skills of first-year house surgeons: A comparison of a simulation workshop versus 6 months of clinical ward experience alone | 2007 | New Zealand | Cohort | Medical doctors | | Surgeons | 10 | N/A | N/A | Lumbar puncture | Year 1 acted as “controls” and allowed the evaluation of clinical experience alone on clinical skills. For Year 2, a clinical skills simulation workshop was organised at the beginning of their first quarter prior to starting clinical work. Both groups were asked to fill out confidence questionnaires at the beginning and end of each quarter and in addition the intervention group filled in a questionnaire post workshop.   Skills workshop consisted of a full day’s training in the skills unit prior to starting clinical work in the first quarter, with the intervention group (Year 2) rotating through various simulation models  covering male catheterisation, ABGs, LP, I/M injection, fundoscopy, ECGs, prostate examination, spirometry, breast examination, and NG tube insertion.  All teaching was by senior medical staff and in small groups of 3–4 PGY1s. All had hands-on experience in trying out the procedures or examinations on the models, as well as the opportunity to discuss any issues. At the end of the workshop PGY1s were asked to fill out repeat questionnaires, as well as an evaluation form to assess the course’s usefulness towards a house surgeon job.   Lumbar puncture was part of a multi-prodedure curriculum. | Simulation model (not elaborated) | Improvment in self-rated competence Improvement in procedural confidence | 6-point Likert scale 5-point Likert scale | 1 | The findings suggest that a skills workshop prior to starting clinical work may approximate 6 months of clinical experience and improve baseline procedural and examination skills. | N/A | 8 |
| Sun 2018 | Evaluation of Problem- and Simulator-Based Learning in Lumbar Puncture in Adult Neurology Residency Training | 2018 | China | Randomized Controlled Trial | Medical doctors | | Neurology | 60 | 60 | N/A | Lumbar puncture | At the beginning of the study, PGY1 residents completed a questionnaire regarding performance level. After completing the questionnaire, an examination assessed residents’ performance of all steps and key points of lumbar puncture on an adult simulator (ZH-L260B), including checking the CSF opening pressure and judging whether the LP procedure was successful.   Group 1 completed LP training with traditional learning methods, whereas Group 2 completed training with PBSL. After pretesting, faculty including senior residents, attending physicians, or consultants who were not permitted to score the test discussed experiences with PGY1 residents in group 1, answered residents’ questions, showed a video of LP, and demonstrated how to perform LP. PGY1 residents in group 2 also attended the LP demonstration and additionally were asked to complete LP simulation training and learned procedure scripting, writing highlights of preparation for LP and LP performance based on specific information of real patients.GY1 residents had the same examination as a posttest. We followed the residents for the next year, assessing successful LPs. Attending or consultant who was blinded regarding the group assignment scored the performance.  After training, we assessed to what extent the PGY1 residents were ready to perform LP and asked them to complete questionnaires about the efficiency of training models. | Lumbar puncture model (Zhonghong, Shanghai, China (ZH-L260B) | Difference in number of succesfull clinical lumbar punctures between groups Improvement in procedural confidence | Clinical metrics (clinical skills checklist) 5-point Likert scale | 4 1 | Compared with traditional teaching model, problem and simulator-based learning for lumbar puncture training can develop overall surgical skills, including technical and nontechnical elements, improving performance. Residents in the problem and simulator based learning group were more confident and effective in performing lumbar puncture. | N/A | 14 |
| Toy 2017 | Using Learner-Centered, Simulation-Based Training to Improve Medical Students' Procedural Skills. | 2017 | United States of America | Cohort study | Medical students | | N/A | 24 | 24 | N/A | Lumbar puncture | Pre/posttraining assessments of confidence, knowledge, and procedural skills were performed using a survey with a 5-point Likert scale, a 40-item multiple-choice test (same items were  used in pre/posttest), and procedural checklists, respectively. For each procedural skill, a station was developed with the appropriate simulation model and other equipment necessary for each specific procedure (not elaborated). Lumbar puncture was part of a multi-procedural curriculum. | Simulation model (not elaborated) | Improvement in procedural confidence Improvement in procedural skills  Satisfaction with simulation-course | 5-point Likert scale Procedural checklist Satisfaction survey | 1 2 1 | The simulation sessions allowed each medical student to receive individual attention from 2 residents for each procedure. Students’ written comments indicated that this training modality was well received. Results showed that medical students improved their self-confidence, knowledge, and skills in the procedures. | None | 12 |
| Udani 2014 | Simulation-Based Mastery Learning with Deliberate Practice Improves Clinical Performance in Spinal Anesthesia | 2014 | USA | Randomised controlled study | Medical doctors | | Anesthesia | 21 | 21 | N/A | Spinal anaesthesia | A baseline assessment of each participant performing a subarachnoid block was made on a task-trainer.The video-recorded performances at baseline were later scored by two authors.  Intervention: Mastery Learning with Deliberate Practice Model. One to 5 days after completion of the base curriculum residents were videotaped performing subarachnoid blocks in the operating room on 3 consenting patients. | Low fidelity phantom (Lumbar Puncture Simulator II, Kyoto Kagaku, Japan) | To determine if adding simulation-based deliberate practice to a base curriculum improved performance of a SAB. | 16-item checklist | 4 | The base curriculum significantly improved resident SAB performance. Deliberate practice training added a significant, independent, incremental benefit. The clinical impact of the deliberate practice intervention in the OR on patient care is unclear. | None | 16 |
| Valentine 2019 | Outcomes of a boot camp for incoming neurology residents | 2019 | United States of America | Cohort study | Medical doctors | | Neurology | N/A | 6 | N/A | Lumbar puncture | A one-week intensive “boot camp” was developed for incoming residents. in place of clinical duties for the first week of July, they completed a series of interactive didactL92:L95ics covering basic neuroanatomy and physiology, the neurologic exam, urgent clinical presentations (stroke, seizure, headache, neuromuscular crises), neuroradiology, and lumbar punctures. The week concluded with a case-based review. To assess efficacy, residents were sent a post-week self-assessment (with both Likert scales and write-in sections) regarding their comfort and preparation for clinical neurology. Faculty members were sent a survey several months later asking them to compare that cohort’s preparation to that of the preceding five years. | N/A | Improvement in self-assessment efficacy Improvement in procedural confidence | Likert scale (not elaborated) Survey (not elaborated) | 1 1 | A one-week “boot camp” may better prepare incoming residents for clinical neurology, and mitigate anxiety related to transQ92:Q93itioning to a new residency program | Dr. Mirasol has stock in PGE Poland. Dr. Mirasol has stock in Exxon Mobil. Dr. Mirasol has stock in EQT. The institution of Dr. Mirasol has received research support from Robert Wood Johnson Medical School. The institution of Dr. Mirasol has received research support from Jannsen Pharmaceutical. | 6,5 |
| Vassallo 2015 | Lumbar puncture training using simulation-based educational strategies. Experience in a clinical pediatric residency | 2015 | Argentina | Cohort study | Medical doctors | | Pediatric | 112 | 112 | Simulation centre | Infant lumbar puncture | Strategies included, in a sequential order, the introduction of theoretical aspects using the bibliography and audiovisual resources available at the hospital’s online campus and subsequent practice of lumbar puncture in a 3-month-old infant phantom on a lateral recumbent position that allowed to make a puncture and collect cerebrospinal fluid. At each training session, the level of confidence was measured before and after the procedure, and a checklist was developed to verify an adequate compliance with each step of the procedure | Low-fidelity infant lumbar puncture phantom (not elaborated) | Improvement in procedural confidence Competence in procedural skills | 5-point Likert scale Clinical checklist | 1 2 | The simulated lumbar puncture training model has been introduced as an educational strategy of our Pediatric Residency Program. | None | 10 |
| Vilasagar 2013 | Does a pediatric lumbar puncture program lead to sustained improvement in resident skill? | 2013 | United States of America | Cohort study | Medical doctors | | Pediatric Emergency medicine | 53 | 53 | N/A | Infant lumbar puncture | The intervention group received training, which consisted of a short didatic session, a 10-minute video, a quiz, and then observed practice on a an infant simulator model. Success rate and number of attempts were compared pre- and post-course, and between intervention and control groups. | Low-fidelity infant lumbar puncture phantom (Laerdal Baby Stap TM) | Improvement in long-term skills retention (defined as success rate and number of attempts) | Self-reported clinical success (obtainment of cerebrospinal fluid) | 4 | Long-term lumbar puncture skill retention was not achieved with a single lumbar puncture course, despite training the trainers. Residents may benefit from frequent lumbar puncture training sessions in order to improve lumbar puncture skill with time. Further studies are needed to determine whether frequent lumbar puncture sessions could improve lumbar puncture skill. | N/A | 12,5 |
| vonCranach 2019 | Medical students' attitudes toward lumbar puncture—And how to change | 2019 | Germany | Cohort study | Medical students | | N/A | 159 | 112 | N/A | Lumbar puncture | The first 45 min consisted of a standardized theoretical overview of various aspects of LP including indications, contraindications, risks, complications and their management, needle types, and analysis of cerebrospinal fluid using projected slides. An extra section covered nontechnical aspects such as the creation of an adequate environment and empathetic communication. After a standardized demonstration by the instructor, each participant had between 15 and 20 min to perform the LP procedure on phantoms. | Low-fidelity phantom (Spinal Simulator 1, 3B Scientific, Hamburg, Germany) | Improvement in self-assessed procedural competence Improvement in procedural confidence | 5-point Likert scale 5-point Likert scale | 1 1 | A single standardized lumbar puncture seminar with simulation training alters medical students' attitudes toward lumbar puncture through improving their level of knowledge and confidence. This may have important implications in doctors‐to‐be on their stance toward  lumbar puncture and resultant advice to future patients regarding this important procedure. | None | 9 |
| Vusse 2020 | Procedure Training Workshop for Internal Medicine Residents that Emphasizes Procedural Ultrasound: Logistics and Teaching Materials | 2020 | United States of America | Cohort study | Medical doctors | | Internal medicine | 204 | 82 | Simulation centre | Lumbar puncture | This 4-hour annual workshop trained interns in basic US skills, US-marked paracentesis, US-marked thoracentesis, US-guided peripheral intravenous catheter placement, and lumbar puncture. A few weeks ahead of orientation, residency program staff emailed participants a list of required preparatory work to complete prior to the workshop. Before attending the workshop, interns viewed several online videos.  Each workshop began in a large group. After interns had completed a brief preworkshop questionnaire in hard copy, the organizer introduced the workshop schedule and locations of the learning stations. Interns then rotated through the learning stations in predetermined small groups of four to five interns. Interns spent the majority of time with their hands on the equipment, practicing manual tasks with verbal guidance frominstructors. Immediately after the workshop, residency program staff emailed all participants a questionnaire we developed to assess the workshop’s impact and gather feedback.  Lumbar puncture was part of a multi-procedural curriculum. | Task trainer (not elaborated) | Improvement in self-assessed independence and confidence | 5-point Likert scale 5-point Likert scale | 1 | This internal medicine intern orientation workshop on procedures and procedural ultrasound was well received and increased participants’ confidence and sense of independence. This publication contains materials needed to reproduce the training experience. | None | 7,5 |
| Wang 2024 | Continuing medical education for attending physicians in anesthesia: Feasibility of an innovative blended learning approach | 2024 | China | Randomized Controlled Trial | Medical doctors | | Anaesthesia | 27 | 27 | Simulation centre | Ultrasound-guided spinal anaesthesia | The control group received instruction through PowerPoint presentations, operation videos, spinal teaching equipment, and 3D body anatomy software. Practical skills training involved simulated teaching equipment at the clinical skills training center, enhancing their operational capabilities.  The experimental group underwent a blended teaching  approach. A private online WeChat group was created to provide online learning support monitored by 2 tutors and for peer discussion. Firstly, a questionnaire was administered to evaluate theoretical knowledge and operational experience of students (pre-assessment). Subsequently, students received PPT materials and operation videos (the first phase of bridge-in), followed by organized online discussions to address student inquiries (the first phase of participatory learning).In the offline sessions, clinical scenarios such as scoliosis or severe obesity was showcased and comprehensive clinical status was analyzed to emphasize the significance of ultrasound-guided spinal anesthesia (bridge-in). Students were guided by tutors to focus on learning objectives (probe orientation, basic views, transverse and paramedian sagittal scan, and accurate identification). For participatory learning, theoretical content was presented by tutors who engaged students through watching videos, using 3D body anatomy software, peer interaction and discussions. Practical skills training  involved simulated teaching equipment.  Operation durations required to locate positions in the simulated teaching equipment were documented for all participating students. | Simulation (not elaborated) | Participants perspectives on the blended learning approach course  Improvement in ultrasound-guided spinal anesthesia performance time following a blended learning approach course | Survey Time | 1 2 | Most participants preferred blended learning as it was more effective than traditional learning. Suggestions for enhancement included enhanced online interactivity with trainers and the inclusion of case analysis. Integration  of blended teaching incorporating BOPPPS and SPOC methodologies holds promise for enhancing the efficiency of skill training among anesthesiologists. Blended learning may become a viable and well-received option among anesthesia clinicians in China. | None | 12,5 |
| Wayne 2014 | Progress toward improving medical school graduates' skills via a "boot camp" curriculum | 2014 | United States of America | Cohort study | Medical students | | N/A | 78 | 78 | Simulation centre | Lumbar puncture | Three cohort comparison groups who completed the same assessments were used to evaluate the impact of the boot camp intervention versus either no intervention or the same boot camp experience delivered 2 months later. The intervention course consisted of 2 days (16 hours) of small group simulation-based education and individualized feedback and skills assessment.  The intervention involved MSOP competencies and specific skills including (a) physical examination techniques (cardiac auscultation); routine technical procedures including (b) paracentesis and (c) lumbar puncture (LP); (d) recognition and management of patients with life-threatening conditions (intensive care unit [ICU] clinical skills/mechanical ventilation); and (e) communication with patients and families. Students were divided into groups of 4 to 6 and rotated through a standardized educational intervention for each of the 5 competencies. For paracentesis and lumbar puncture procedures, students received a demonstration of a procedure using a paracentesis simulator developed at Northwestern and the Kyoto Kagaku (Torrance, CA) lumbar puncture simulator and engaged in deliberate practice of each procedure with feedback from a trained faculty facilitator. Students completed a skills assessment after each educational intervention.   Lumbar puncture was part of a multi-procedural curriculum. | Low-fidelity phantom (Kyoto Kagaku, Torrance, CA) | Improvement in procedural performance Improvement in procedural self-confidence Participants perception of course | Lumbar puncture checklist  100-point Likert scale 5-point Likert scale | 2 1 1 | A 2-day simulation-based boot camp for graduating medical students boosted a variety of clinical skills to levels significantly higher than PGY-1 historical controls. Simulation-based education shows promise to help ensure that medical school graduates are prepared to begin postgraduate training. | None | 14,5 |
| Weil 2017 | Learning curves for three specific procedures by anesthesiology residents using the learning curve cumulative sum (LC-CUSUM) test | 2017 | France | Observational study | Medical doctors | | Anaesthesia | 18 | 18 | Clinical department | Thoracic epidural anaesthesia | During three consecutive rotations, data from 18 residents (six per rotation) were collected for all performed procedures. The procedure was considered a failure if epidural  analgesia was insufficient in the postoperative care unit, if more than two punctures were needed, if the resident was unable to insert the catheter, or if the dura mater was perforated when the catheter was inserted.  Epidural anesthesia was among multiple monitored procedures | Patients | To monitor the learning process and failure rate of anaesthesia residents training for specific subspecialty anaesthesia procedures. | Failure: Resident unable  to insert the catheter  more than two attempts  inefficient analgesia  dura mater punctures  Monitored using LC-CUSUM | 4 | A single 6-month rotation in a reference teaching centre may not be sufficient to train residents to perform specific or sub-specialty procedures as required. A regional learning network may be useful. More patient-based data are  necessary to conduct a risk adjustment analysis for such specific procedures. | N/A | 10 |
| Westwood 2012 | Lumbar puncture simulation training improves medical student knowledge and confidence | 2012 | United States of America | Cohort study | Medical students | | N/A | 17 | 17 | N/A | Lumbar puncture | Medical students during the neurology rotation were surveyed on their perspective on lumbar puncture simulation (not further elaborated). | Simulator (not further elaborated) | Improvement i procedural confidence | 5-point Likert scale | 1 | Medical students find the lumbar puncture simulation training useful. It increases their levels of confidence and understanding of the indications and risks and benefits of the procedure. Further study into the translation of simulation expertise to positive procedure outcome will be explored. | Dr. Westwood has nothing to disclose. Dr. Hohler has received personal compensation for activities with Teva Neuroscience as a speaker. | 7,5 |
| White 2012 | Transfer of simulated lumbar puncture training to the clinical setting | 2012 | United States of America | Cohort study | Medical doctors | | Paediatric Internal medicine-paediatric | 23 | 21 | Clinical department | Infant lumbar puncture | Before the training course, all of the interns were given a pretest and survey that assessed their baseline knowledge of lumbar punctures.  The residents participated in a 1-hour LP training course. The course consisted of a brief evidence-based presentation, reviewing anatomy, indications, complications, and techniques for performing LPs followed by hands-on practice of LPs. After this presentation, all participants individually demonstrated LP performance on a neonatal mannequin using the Baby Stap model (Laerdal, Wappingers Falls, NY). To better recreate an actual experience, sterile gloves, lumbar puncture trays, pressure manometers, and mock parental consents with actual forms were used during the simulation. After completing the LP training course, all the interns were individually assessed while performing an LP on an actual pediatric patient. A standardized checklist (Table 1) was used that consisted of 10 steps, with one 2-part step for a total of 11 actions to perform. The follow-up assessment occurred, on average, 5 months after the initial training course. | Low fidelity phantom (Baby Stap model (Laerdal, Wappingers Falls, NY) | Improvement in procedural confidence Improvement in procedural skills | 5-point Likert scale Clinical measure (standardized checklist) | 1 4 | A task trainer-based course improved the confidence and knowledge about an important paediatric procedure. This confidence and knowledge can translate to actual clinical practice. Further investigations are necessary to support this knowledge and skill translation. | N/A | 13,5 |
| Wiggins 2018 | Using evidence-based best practices of simulation, checklists, deliberate practice, and debriefing to develop and improve a regional anesthesia training course | 2018 | United States of America | Cohort study | Nurses | | Anaesthesia | 52 | 49 | N/A | Spinal and epidural anaesthesia | Development of the blended curriculum for spinal and epidural anaesthesia training included online pre-course didactic content, checklists, deliberate practice with mastery learning elements, and experiential learning with expert instructors. Completion of online materials took approximately 4 hours, as reported by the expert instructors.  Participants were required to achieve a threshold of 80% on the baseline assessment in order to progress to the hands-on component. A precourse/postcourse survey on attitudes was administered. The hands-on practice included use of 2 specific task trainers | Low-fidelity phantom (Life/form Spinal Injection Simulator, Nasco Inc)  Low-fidelity phantom (The Obese Adult Lumbar Puncture/Epidural trainer (Simulab)) | Improvement in procedural confidence and comfort  Participants perception of course value | Survey (not elaborated) Survey (not elaborated) | 1 | Forty-nine CRNAs completed all course components, including meeting all skill training thresholds through deliberate practice and use of validated checklists. Knowledge and confidence levels demonstrated significant gains. | None | 8,5 |
| Williams 2018 | Simulation-based mastery learning improves lumbar puncture but not paracentesis performance | 2018 | United States of America | Randomized Controlled Trial | Medical doctors | | N/A | 88 | 88 | N/A | Ultrasound-guided lumbar puncture | The SBT cohort received 4 hours of instruction on ultrasound-guided lumbar puncture and paracentesis task trainers. The session followed an evidenced-based pedagogical framework that included readings and videos prior to the session as well as simulation-based mastery learning. Three months after the training, learners completed a 5-point Likert scale asking if they were comfortable performing the procedure without supervision and/or teaching the procedure to others.  Following the training, when the interns performed these procedures on patients, their supervisors used a validated global rating scale to rate their performance as novice, beginner, competent, or proficient.  Lumbar puncture was part of multi-procedural curriculum. | Lumbar puncture task trainers (not elaborated) | Improvement in comfort performing the procedure without supervision  Comfort teaching the procedure to others  Improvement in clinical lumbar puncture performance | 5-point Likert scale 5-point Likert scale A validated global rating scale | 1 1 4 | Though no subjective change in paracentesis and lumbar puncture confidence was found, an objective improvement in the interns’ performance of lumbar punctures on patients was seen after simulation-based training. Simulation-based training likely failed to improve performance of paracentesis because of the already high level of competence, confidence and knowledge of this procedure within the control group. Further study is needed to investigate whether this improved knowledge of paracentesis and lumbar puncture results in reduced procedural complications after simulation-based training. | N/A | 15 |
| Wong 2018 | Developing a child neurology training program in Cambodia: A pilot study | 2018 | Cambodia | Cohort study | Medical doctors | | Paediatric Internal Medicine Psychiatry Neurosurgery | 40 | 30 | N/A | Lumbar puncture | An intensive one-week course covering a wide range of child neurology topics. A multi-pronged teaching style including didactics, interactive small groups, peer simulation (lumbar puncture and neurologic exam skills), and hospital bedside rounds was implemented. The course was taught by a child neurology resident, adult neurology resident, and adult neurology professor. Topics were accompanied by pre/post MCQ tests, and attitude questions using Likert scales. Lumbar puncture was part of a multi-procedural curriculum. | Simulation (not elaborated) | Improvement in procedural confidence | Likert scale (not elaborated) | 1 | Intensive short courses in child neurology with emphasis on interactive and multi-modal learning can successfully bridge the knowledge gap amongst medical providers in Cambodia. The success of this course provides a roadmap for other educators to implement training programs in resource limited settings through utilizing modalities conventional to US medical training programs but considered novel to some developing institutions. | N/A | 7,5 |
| Xie 2023 | Using a novel virtual-reality simulator to assess performance in lumbar puncture: a validation study | 2023 | China | Cohort study | Medical students and MDs | | neurosurgery departments, departments of internal medicine (including neurology, emergency care unit), and  anaesthesiology | 110 | 110 | Simulation centre | Lumbar puncture | A 5-minute video illustrating a virtual reality simulation of a lumbar puncture was shown to each participant before the test. Then each participant performed a  lumbar puncture procedure on the simulator. | Virtual Reality Lumbar Puncture simulator (Virtual  Puncture Surgery Platform, CXV-CS-PVO80, Shanghai, China) | to investigate validity evidence for a simulator-based test in lumbar puncture and establish a pass/fail standard | Simulator metrics | 2 | This study provides validity evidence for a simulator-based test of lumbar puncture competence. The test can help ensure basic competence at the end of a simulation-based training program for trainees, i.e., a mastery  learning training program. | None | 13,5 |
| Yanta 2020 | The Use of Hybrid Lumbar Puncture Simulation to Teach Entrustable Professional Activities During a Medical Student Neurology Clerkship. | 2020 | United States of America | Cohort study | Medical students | | N/A | 84 | 70 | N/A | Lumbar puncture | Within the first four days of the clerkship, the students completed an online Likert-style survey assessing their level of confidence and perceived skill with these activities.   On the fifth day of the 28-day clerkship, the students then gathered for the hybrid simulation experience. During this experience, they first simulated obtaining informed consent for a lumbar puncture from a standardized patient with a chief complaint of monocular vision loss concerning for optic neuritis. Feedback was immediately provided to the students based on an informed consent checklist provided to the standardized patients. The students then received instruction from neurology faculty or senior resident preceptors on performing LP with the use of a specialized manikin. They were then able to practice the procedure with real-time feedback provided by preceptors. The students then entered orders on the “CSF” into a simulated EMR, again obtaining real-time feedback from preceptors. Following the simulation exercise, the students filled out a similar Likert-style survey to assess their confidence and perceived skill with these three activities. | Manikin (not elaborated) | Improvement in self-perceived confidence and self-perceived skills | Likert scale (not elaborated) | 1 | Hybrid LP simulation was effective in increasing medical student confidence and perceived skill with EPAs 4, 11, and 12. | None | 8,5 |
| Yee 2022 | Procedural Curriculum to Verify Intern Competence Prior to Patient Care. | 2022 | United States of America | Cohort study | Medical doctors | | Emergency medicine | 38 | 38 | Simulation centre | Lumbar puncture | This included asynchronous pre-training with videos followed by a face-to-face training event. These videos, which included the New England Journal of Medicine procedure series (Year One), were assigned prior to the resident’s scheduled time in the simulation laboratory. Participants were then provided face-to-face training including pretesting assessments, faculty demonstration,  time for deliberate practice, and a post-test to confirm the achievement of competence. This experience was facilitated by faculty who were trained to teach the five procedures and score the checklist assessments. Face-to-face skills training events were held over three days to accommodate all interns.  One faculty member was assigned to each participant for the day. During pretesting, checklists were used to obtain participants’ individual baseline performance on the procedures.  Each participant’s performance was compared against the MPS derived from standard setting. Afterward, a faculty member then demonstrated the procedures for the group of participants. Participants were able to ask questions and were given clarity on critical procedural steps. Following this, each participant then returned to their assigned faculty member for deliberate practice and receipt of individualized feedback. Participants were given up to 45 minutes of deliberate practice per procedure. At a minimum, the individual participant’s time commitment for all five procedures was estimated at nine hours if they were able to pass each post-test on their first attempt after one session of deliberate practice.  Lumbar puncture was part of a multi-procedural curriculum. | Task trainers (not elaborated) | Improvement in procedural skills | Procedural checklist with a minimum passing score | 2 | All incoming first-year EM residents demonstrated procedural competence on five different procedures using a mastery-based educational framework. A competency-based EM curriculum allowed for demonstration of procedural competence prior to resident participation in supervised clinical patient care. | None | 12 |
| Yeo 2015 | Examination of learning trajectories for simulated lumbar puncture training using hand motion analysis | 2015 | Canada | Cohort study | Medical students | | N/A | 25 | 25 | Simulation centre | Lumbar puncture | Study subjects completed a total of three or four sessions, depending on when stable proficiency was reached. There were intervals of 6 weeks between each session. The study was terminated after session 4 as the majority of subjects had reached stable proficiency at this point. Each subject spent approximately 30 minutes to 1 hour per session at the Queen’s University Clinical Simulation Centre. Prior to session 1, subjects were instructed to complete an online LP module that introduced the indications and risks of the procedure, key anatomical landmarks, and the standard equipment used for LP.   At session 2, subjects underwent hand-motion-analysis assessment on arrival and were given immediate feedback. Subjects were instructed to practice the entire procedure until they felt ready to be reassessed. At sessions 3 and 4, each subject completed an hand-motion-analysis assessment at arrival. Subjects who demonstrated technical proficiency at the outset of the session were deemed stably proficient and excused from further training. Subjects who did not display stable proficiency proceeded with practice and reassessment until two consecutive benchmarks were achieved. Only subjects who did not demonstrate stable proficiency at the outset of session 3 were scheduled for session 4  For this study, technical proficiency was defined as the completion of two consecutive hand-motion-analysis assessments that met expert benchmarks for the metrics of total procedure time and translational and rotational hand motion. | Low-fidelity phantom (The Lumbar Puncture Simulator II manikin (Kyoto Kagaku, Japan)) | Achievement of procedural proficiency  Skills retention | A modified procedural checklist | 2 | These results show that the majority of students require three to four sessions of deliberate practice to achieve a sustainable level of proficiency in the LP procedure. There is considerable variation in learning progression and retention of technical proficiency. These results have important implications for the design and resource requirements of a competency-based medical education program targeting LP training. | None | 12 |
