# Supplementary material for: Training and assessment of skills in neuraxial space access: a scoping review of educational approaches to lumbar puncture, epidural anaesthesia, and spinal anaesthesia
Source: Br J Anaesth. 2025 Jul 7;135(4):1026–37. doi: 10.1016/j.bja.2025.06.008 (PMC12674033; doi:10.1016/j.bja.2025.06.008)
Supplement: Multimedia component 3 [file mmc3.docx]

*Appendix 3 – Study characteristics*

| **Country of study, n= 99** | **n (%)** |
| --- | --- |
| United States of America | 54 (55) |
| Canada | 10 (10) |
| Ireland | 5 (5) |
| China | 4 (4) |
| France | 3 (3) |
| United Kingdom | 3 (3) |
| New Zealand | 2 (2) |
| Denmark | 2 (2) |
| Germany | 2 (2) |
| Singapore | 2 (2) |
| Argentina | 1 (1) |
| Australia | 1 (1) |
| Cambodia | 1 (1) |
| Chile | 1 (1) |
| Colombia | 1 (1) |
| Iran | 1 (1) |
| Japan | 1 (1) |
| Libya | 1 (1) |
| Mexico | 1 (1) |
| Saudi Arabia | 1 (1) |
| Spain | 1 (1) |
| Switzerland | 1 (1) |
| **Year of publication** | |
| 2020-2024 | 26 (26) |
| 2015-2019 | 34 (34) |
| 2010-2014 | 27 (27) |
| - 2009 | 12 (12) |
| **Study design** | |
| Cohort study | 67 (68) |
| Randomised controlled trial | 24 (24) |
| Observational study | 8 (8) |
| **Population** | |
| Medical doctors | 72 (73) |
| Medical students | 22 (22) |
| Nurses | 1 (1) |
| Combined, both medical doctors, medical students, and nurses | 4 (4) |
| **Medical specialty** | |
| Anaesthesia | 22 (22) |
| Paediatrics | 17 (17) |
| Internal medicine (not further elaborated) | 11 (11) |
| Multiple specialties | 11 (11) |
| Emergency medicine | 4 (4) |
| Neurology | 2 (2) |
| Geriatrics | 1 (1) |
| Surgeons | 1 (1) |
| Not reported | 30 (30) |
