# Supplementary material for: Training and assessment of skills in neuraxial space access: a scoping review of educational approaches to lumbar puncture, epidural anaesthesia, and spinal anaesthesia
Source: Br J Anaesth. 2025 Jul 7;135(4):1026–37. doi: 10.1016/j.bja.2025.06.008 (PMC12674033; doi:10.1016/j.bja.2025.06.008)
Supplement: Multimedia component 4 [file mmc4.docx]

*Appendix 4 – Setting, skills trained, and training modalities*

| **Setting (as categorized in articles)** | **n (%)** |
| --- | --- |
| Simulation Centre | 25 (25) |
| Clinical department | 24 (24) |
| Simulation Centre and clinical department | 4 (4) |
| Cadaver laboratory | 2 (2) |
| Simulation Centre and cadaver laboratory | 1 (1) |
| University and clinical department | 1 (1) |
| Not reported | 42 (42) |
| **Procedure** | **n (%)** |
| Adult lumbar puncture | 50 (51) |
| Infant lumbar puncture | 15 (15) |
| Epidural anaesthesia | 9 (9) |
| Spinal and epidural anaesthesia | 7 (7) |
| Paediatric lumbar puncture | 6 (6) |
| Spinal anaesthesia | 4 (4) |
| Ultrasound-assisted lumbar puncture | 2 (2) |
| Ultrasound-guided lumbar puncture | 2 (2) |
| Epidural anaesthesia and ultrasound-assisted epidural anaesthesia | 1 (1) |
| Lumbar puncture and spinal anaesthesia | 1 (1) |
| Ultrasound-assisted paediatric lumbar puncture | 1 (1) |
| Ultrasound-guided spinal anaesthesia | 1 (1) |
| **Hands-on modality (some studies included more than one modality)** | |
| Low-fidelity mannequins | 54 |
| Simulation (not elaborated) | 18 |
| Live models | 13 |
| Mixed reality | 4 |
| Cadaver | 3 |
| Fruit (banana or orange) | 3 |
| High-fidelity model | 2 |
| Virtual reality | 1 |
| Not reported | 6 |
