# Supplementary material for: Training and assessment of skills in neuraxial space access: a scoping review of educational approaches to lumbar puncture, epidural anaesthesia, and spinal anaesthesia
Source: Br J Anaesth. 2025 Jul 7;135(4):1026–37. doi: 10.1016/j.bja.2025.06.008 (PMC12674033; doi:10.1016/j.bja.2025.06.008)
Supplement: Multimedia component 5 [file mmc5.docx]

*Appendix 5 – outcome measurement tools, and Kirkpatrick level for outcome*

| **Outcome measurement tool (some studies included more than one)** | **n** |
| --- | --- |
| Likert scale-based self-assessment | 43 |
| Clinical metrics | 33 |
| Procedural checklist | 27 |
| Survey (not elaborated) | 17 |
| Global rating scale | 12 |
| Objective Structured Clinical Examination (OSCE) | 6 |
| Other or not reported | 5 |
| **Kirkpatrick level for outcomes (some studies included more than one outcome)** | |
| Kirkpatrick level 1 | 57 |
| Kirkpatrick level 2 | 34 |
| Kirkpatrick level 3 | 6 |
| Kirkpatrick level 4 | 34 |
