# Supplementary material for: Training and assessment of skills in neuraxial space access: a scoping review of educational approaches to lumbar puncture, epidural anaesthesia, and spinal anaesthesia
Source: Br J Anaesth. 2025 Jul 7;135(4):1026–37. doi: 10.1016/j.bja.2025.06.008 (PMC12674033; doi:10.1016/j.bja.2025.06.008)
Supplement: Multimedia component 6 [file mmc6.docx]

| **Author and year** | **Title** | **Study design, points** | **Sampling: Institutions, points** | **Sampling: Response rate, points** | **Type of data, points** | **Validity evidence for evaluation instrument: Internal structure, points** | **Validity evidence for evaluation instrument: Content, points** | **Validity evidence for evaluation instrument: Relationships to other variables, points** | **Data analysis: Appropriate for study design and type of data, points** | **Data analysis: Complexity, points** | **Outcome, points** | **Total score, points** |
| --- | --- | --- | --- | --- | --- | --- | --- | --- | --- | --- | --- | --- |
| Adachi 2012 | Clinical clerkship course for medical students on lumbar puncture using simulators | 1 (Single group cross-sectional or single group posttest only) | 0.5 (1 institution) | 1.5 (more than 75%) | 1 (Assessment by study participant) | 0 (Not reported) | 0 (Not reported) | 0 (Not reported) | 1 (Data analysis appropriate for study design and type of data) | 1 (Descriptive analysis only) | 1 (Satisfaction, attitudes, perceptions, opinions, general facts) | 7 |
| Adam 2022 | Clinical Skills Day: A Novel Approach to Enhancing Procedural Skills Teaching for Foundation Year One Doctors. | 1.5 (Single group pretest and posttest) | 0.5 (1 institution) | 0.5 (less than 50% or not reported) | 1 (Assessment by study participant) | 0 (Not reported) | 0 (Not reported) | 0 (Not reported) | 1 (Data analysis appropriate for study design and type of data) | 2 (Beyond descriptive analysis) | 1 (Satisfaction, attitudes, perceptions, opinions, general facts) | 7,5 |
| AguirreOspina 2014 | Cumulative Sum learning curves (CUSUM) in basic anaesthesia procedures | 1 (Single group cross-sectional or single group posttest only) | 0.5 (1 institution) | 1.5 (more than 75%) | 1 (Assessment by study participant) | 0 (Not reported) | 1 (Reported) | 0 (Not reported) | 1 (Data analysis appropriate for study design and type of data) | 1 (Descriptive analysis only) | 3 (Patient/health care outcome) | 10 |
| Antonenko 2021 | Improving lumbar puncture technique among intern trainees to enhance quality of care for patients | 1 (Single group cross-sectional or single group posttest only) | 0.5 (1 institution) | 1 (50%-74%) | 1 (Assessment by study participant) | 0 (Not reported) | 0 (Not reported) | 0 (Not reported) | 1 (Data analysis appropriate for study design and type of data) | 1 (Descriptive analysis only) | 1 (Satisfaction, attitudes, perceptions, opinions, general facts) | 6,5 |
| Auerbach 2016 | The correlation of workplace simulation-based assessments with interns' infant lumbar puncture success a prospective, multicenter, observational study | 1 (Single group cross-sectional or single group posttest only) | 1,5 (more than 2 institutions) | 0.5 (less than 50% or not reported) | 3 (Objective measurement) | 1 (Reported) | 1 (Reported) | 1 (Reported) | 1 (Data analysis appropriate for study design and type of data) | 2 (Beyond descriptive analysis) | 3 (Patient/health care outcome) | 15 |
| Augustine 2012 | Effect of procedure simulation workshops on resident procedural confidence and competence. | 1.5 (Single group pretest and posttest) | 0.5 (1 institution) | 1.5 (more than 75%) | 1 (Assessment by study participant) | 0 (Not reported) | 0 (Not reported) | 1 (Reported) | 1 (Data analysis appropriate for study design and type of data) | 2 (Beyond descriptive analysis) | 1 (Satisfaction, attitudes, perceptions, opinions, general facts) | 9,5 |
| Barsuk 2012 | Simulation-based education with mastery learning improves residents' lumbar puncture skills | 2 (Nonrandomized, 2 group) | 1,5 (more than 2 institutions) | 1.5 (more than 75%) | 3 (Objective measurement) | 1 (Reported) | 1 (Reported) | 1 (Reported) | 1 (Data analysis appropriate for study design and type of data) | 2 (Beyond descriptive analysis) | 1.5 (Knowledge, skills) | 15,5 |
| Bisgaard 2021 | Early procedural training increases anesthesiology residents’ clinical production: a comparative pre-post study of the payoff in clinical training | 2 (Nonrandomized, 2 group) | 1,5 (more than 2 institutions) | 1.5 (more than 75%) | 3 (Objective measurement) | 0 (Not reported) | 1 (Reported) | 1 (Reported) | 1 (Data analysis appropriate for study design and type of data) | 2 (Beyond descriptive analysis) | 2 (Behaviors) | 15 |
| Boggs 2022 | Procedural Fundamentals for Medical Students: Institutional Outcomes of a Novel Multimodal Course | 1.5 (Single group pretest and posttest) | 0.5 (1 institution) | 0.5 (less than 50% or not reported) | 1 (Assessment by study participant) | 0 (Not reported) | 0 (Not reported) | 0 (Not reported) | 1 (Data analysis appropriate for study design and type of data) | 2 (Beyond descriptive analysis) | 1 (Satisfaction, attitudes, perceptions, opinions, general facts) | 7,5 |
| Braun 2017 | Can Residents Assess Other Providers' Infant Lumbar Puncture Skills?: Validity Evidence for a Global Rating Scale and Subcomponent Skills Checklist | 1 (Single group cross-sectional or single group posttest only) | 0.5 (1 institution) | 1.5 (more than 75%) | 3 (Objective measurement) | 1 (Reported) | 1 (Reported) | 1 (Reported) | 1 (Data analysis appropriate for study design and type of data) | 2 (Beyond descriptive analysis) | 1.5 (Knowledge, skills) | 13,5 |
| Brydges 2022 | Mastery versus invention learning: impacts on future learning of simulated procedural skills | 3 (Randomized controlled trial) | 1 (2 institutions) | 1.5 (more than 75%) | 3 (Objective measurement) | 1 (Reported) | 1 (Reported) | 0 (Not reported) | 1 (Data analysis appropriate for study design and type of data) | 2 (Beyond descriptive analysis) | 1.5 (Knowledge, skills) | 15 |
| Brydges 2012 | Directed self-regulated learning versus instructor-regulated learning in simulation training | 3 (Randomized controlled trial) | 0.5 (1 institution) | 1 (50%-74%) | 3 (Objective measurement) | 1 (Reported) | 0 (Not reported) | 1 (Reported) | 1 (Data analysis appropriate for study design and type of data) | 2 (Beyond descriptive analysis) | 1.5 (Knowledge, skills) | 14 |
| Burgess 2011 | Implementation of a Lumbar Puncture (LP) simulation teaching module | 1.5 (Single group pretest and posttest) | 0.5 (1 institution) | 1.5 (more than 75%) | 1 (Assessment by study participant) | 0 (Not reported) | 0 (Not reported) | 0 (Not reported) | 1 (Data analysis appropriate for study design and type of data) | 1 (Descriptive analysis only) | 1 (Satisfaction, attitudes, perceptions, opinions, general facts) | 7,5 |
| Cheung 2019 | Why Content and Cognition Matter: Integrating Conceptual Knowledge to Support Simulation-Based Procedural Skills Transfer | 3 (Randomized controlled trial) | 0.5 (1 institution) | 1.5 (more than 75%) | 3 (Objective measurement) | 1 (Reported) | 1 (Reported) | 1 (Reported) | 1 (Data analysis appropriate for study design and type of data) | 2 (Beyond descriptive analysis) | 1.5 (Knowledge, skills) | 15,5 |
| Chuan 2015 | Design and validation of the Regional Anaesthesia Procedural Skills Assessment Tool. | 1 (Single group cross-sectional or single group posttest only) | 0.5 (1 institution) | 1.5 (more than 75%) | 3 (Objective measurement) | 1 (Reported) | 1 (Reported) | 1 (Reported) | 1 (Data analysis appropriate for study design and type of data) | 2 (Beyond descriptive analysis) | 1.5 (Knowledge, skills) | 13,5 |
| Ciccotto 2012 | Lumbar puncture teaching module standardization. Ensuring patient safety through proper training | 1.5 (Single group pretest and posttest) | 0.5 (1 institution) | 1.5 (more than 75%) | 1 (Assessment by study participant) | 0 (Not reported) | 0 (Not reported) | 0 (Not reported) | 1 (Data analysis appropriate for study design and type of data) | 2 (Beyond descriptive analysis) | 1 (Satisfaction, attitudes, perceptions, opinions, general facts) | 8,5 |
| Cohen 2013 | Making july safer: Simulation-based mastery learning during intern boot camp | 2 (Nonrandomized, 2 group) | 0.5 (1 institution) | 1.5 (more than 75%) | 3 (Objective measurement) | 1 (Reported) | 1 (Reported) | 0 (Not reported) | 1 (Data analysis appropriate for study design and type of data) | 2 (Beyond descriptive analysis) | 1.5 (Knowledge, skills) | 13,5 |
| Conroy 2010 | Competence and retention in performance of the lumbar puncture procedure in a task trainer model | 1.5 (Single group pretest and posttest) | 0.5 (1 institution) | 1.5 (more than 75%) | 3 (Objective measurement) | 1 (Reported) | 1 (Reported) | 1 (Reported) | 1 (Data analysis appropriate for study design and type of data) | 2 (Beyond descriptive analysis) | 1.5 (Knowledge, skills) | 14 |
| Corvetto 2017 | Validation of the imperial college surgical assessment device for spinal anesthesia | 1 (Single group cross-sectional or single group posttest only) | 0.5 (1 institution) | 1.5 (more than 75%) | 3 (Objective measurement) | 1 (Reported) | 1 (Reported) | 1 (Reported) | 1 (Data analysis appropriate for study design and type of data) | 2 (Beyond descriptive analysis) | 1.5 (Knowledge, skills) | 13,5 |
| Crichlow 2017 | Integration of a simulation based mastery learning lumbar puncture curriculum using observational learning into an emergency medicine intern boot camp | 1 (Single group cross-sectional or single group posttest only) | 0.5 (1 institution) | 1.5 (more than 75%) | 3 (Objective measurement) | 0 (Not reported) | 0 (Not reported) | 1 (Reported) | 1 (Data analysis appropriate for study design and type of data) | 2 (Beyond descriptive analysis) | 1.5 (Knowledge, skills) | 11,5 |
| Dadoun 2015 | Peer teaching: An effective method for simulation-based instruction | 3 (Randomized controlled trial) | 0.5 (1 institution) | 1.5 (more than 75%) | 3 (Objective measurement) | 0 (Not reported) | 1 (Reported) | 0 (Not reported) | 1 (Data analysis appropriate for study design and type of data) | 2 (Beyond descriptive analysis) | 1.5 (Knowledge, skills) | 13,5 |
| Drake 2015 | Defining competence in obstetric epidural anaesthesia for inexperienced trainees | 1 (Single group cross-sectional or single group posttest only) | 0.5 (1 institution) | 1.5 (more than 75%) | 3 (Objective measurement) | 0 (Not reported) | 1 (Reported) | 0 (Not reported) | 1 (Data analysis appropriate for study design and type of data) | 1 (Descriptive analysis only) | 3 (Patient/health care outcome) | 12 |
| Dugan 2023 | Assessing Best Practices in a Simulated Lumbar Puncture Workshop with Medical Students | 3 (Randomized controlled trial) | 0.5 (1 institution) | 0.5 (less than 50% or not reported) | 3 (Objective measurement) | 0 (Not reported) | 0 (Not reported) | 0 (Not reported) | 1 (Data analysis appropriate for study design and type of data) | 2 (Beyond descriptive analysis) | 1.5 (Knowledge, skills) | 11,5 |
| Elhadi 2020 | Informed self-assessment versus preceptor evaluation: A comparative study of pediatric procedural skills acquisition of fifth year medical students | 1.5 (Single group pretest and posttest) | 0.5 (1 institution) | 1.5 (more than 75%) | 3 (Objective measurement) | 1 (Reported) | 1 (Reported) | 1 (Reported) | 1 (Data analysis appropriate for study design and type of data) | 2 (Beyond descriptive analysis) | 1.5 (Knowledge, skills) | 14 |
| Fischer 2017 | A simulation-based longitudinal procedural curriculum for pediatric residents improves self perceived competence | 1.5 (Single group pretest and posttest) | 0.5 (1 institution) | 0.5 (less than 50% or not reported) | 1 (Assessment by study participant) | 0 (Not reported) | 0 (Not reported) | 0 (Not reported) | 1 (Data analysis appropriate for study design and type of data) | 2 (Beyond descriptive analysis) | 1 (Satisfaction, attitudes, perceptions, opinions, general facts) | 7,5 |
| Friedman 2006 | Objective Assessment of Manual Skills and Proficiency in Performing Epidural Anesthesia-Video-Assisted Validation | 1 (Single group cross-sectional or single group posttest only) | 0.5 (1 institution) | 1.5 (more than 75%) | 3 (Objective measurement) | 1 (Reported) | 1 (Reported) | 1 (Reported) | 1 (Data analysis appropriate for study design and type of data) | 2 (Beyond descriptive analysis) | 3 (Patient/health care outcome) | 15 |
| Friedman 2009 | Clinical impact of epidural anesthesia simulation fidelity on short and long term learning curve | 3 (Randomized controlled trial) | 0.5 (1 institution) | 1.5 (more than 75%) | 3 (Objective measurement) | 1 (Reported) | 1 (Reported) | 0 (Not reported) | 1 (Data analysis appropriate for study design and type of data) | 2 (Beyond descriptive analysis) | 3 (Patient/health care outcome) | 16 |
| Gaies 2009 | Reforming procedural skills training for pediatric residents: A randomized, interventional trial | 3 (Randomized controlled trial) | 0.5 (1 institution) | 1.5 (more than 75%) | 3 (Objective measurement) | 0 (Not reported) | 0 (Not reported) | 0 (Not reported) | 1 (Data analysis appropriate for study design and type of data) | 2 (Beyond descriptive analysis) | 1.5 (Knowledge, skills) | 12,5 |
| Galen 2019 | A Curriculum for Lumbar Puncture Training in Internal Medicine Residency. | 2 (Nonrandomized, 2 group) | 0.5 (1 institution) | 1 (50%-74%) | 3 (Objective measurement) | 0 (Not reported) | 0 (Not reported) | 0 (Not reported) | 1 (Data analysis appropriate for study design and type of data) | 2 (Beyond descriptive analysis) | 3 (Patient/health care outcome) | 12,5 |
| Gandhi 2023 | ULTRASOUND-ASSISTED LUMBAR PUNCTURE: A QUALITY IMPROVEMENT PROJECT | 1.5 (Single group pretest and posttest) | 0.5 (1 institution) | 0.5 (less than 50% or not reported) | 1 (Assessment by study participant) | 0 (Not reported) | 0 (Not reported) | 1 (Reported) | 1 (Data analysis appropriate for study design and type of data) | 2 (Beyond descriptive analysis) | 1 (Satisfaction, attitudes, perceptions, opinions, general facts) | 8,5 |
| Garrood 2010 | A structured course teaching junior doctors invasive medical procedures results in sustained improvements in self-reported confidence | 1.5 (Single group pretest and posttest) | 0.5 (1 institution) | 1 (50%-74%) | 1 (Assessment by study participant) | 0 (Not reported) | 0 (Not reported) | 0 (Not reported) | 1 (Data analysis appropriate for study design and type of data) | 2 (Beyond descriptive analysis) | 1 (Satisfaction, attitudes, perceptions, opinions, general facts) | 8 |
| Gaubert 2021 | Positive effects of lumbar puncture simulation training for medical students in clinical practice | 3 (Randomized controlled trial) | 0.5 (1 institution) | 0.5 (less than 50% or not reported) | 3 (Objective measurement) | 0 (Not reported) | 0 (Not reported) | 0 (Not reported) | 1 (Data analysis appropriate for study design and type of data) | 2 (Beyond descriptive analysis) | 3 (Patient/health care outcome) | 13 |
| Gerard 2013 | Validation of Global Rating Scale and Checklist Instruments for the Infant Lumbar Puncture Procedure | 1 (Single group cross-sectional or single group posttest only) | 0.5 (1 institution) | 1.5 (more than 75%) | 3 (Objective measurement) | 1 (Reported) | 1 (Reported) | 1 (Reported) | 1 (Data analysis appropriate for study design and type of data) | 2 (Beyond descriptive analysis) | 1.5 (Knowledge, skills) | 13,5 |
| Goldman 2022 | A workplace procedure training cart to augment pediatric resident procedural learning | 1 (Single group cross-sectional or single group posttest only) | 0.5 (1 institution) | 1 (50%-74%) | 3 (Objective measurement) | 0 (Not reported) | 0 (Not reported) | 0 (Not reported) | 1 (Data analysis appropriate for study design and type of data) | 2 (Beyond descriptive analysis) | 2 (Behaviors) | 10,5 |
| Goldman 2022 | Formative Assessments Promote Procedural Learning and Engagement for Senior Pediatric Residents on Rotation in the Pediatric Emergency Department | 1.5 (Single group pretest and posttest) | 0.5 (1 institution) | 1.5 (more than 75%) | 3 (Objective measurement) | 0 (Not reported) | 1 (Reported) | 0 (Not reported) | 1 (Data analysis appropriate for study design and type of data) | 2 (Beyond descriptive analysis) | 2 (Behaviors) | 12,5 |
| Goolsby 2014 | Hybrid simulation improves medical student procedural confidence during EM clerkship | 1.5 (Single group pretest and posttest) | 1,5 (more than 2 institutions) | 0.5 (less than 50% or not reported) | 1 (Assessment by study participant) | 0 (Not reported) | 0 (Not reported) | 0 (Not reported) | 0 (Data analysis inappropriate for study design or type of data) | 2 (Beyond descriptive analysis) | 1 (Satisfaction, attitudes, perceptions, opinions, general facts) | 7,5 |
| Grau 2003 | Ultrasound imaging improves learning curves in obstetric epidural anesthesia: A preliminary study | 3 (Randomized controlled trial) | 0.5 (1 institution) | 1.5 (more than 75%) | 3 (Objective measurement) | 0 (Not reported) | 0 (Not reported) | 0 (Not reported) | 1 (Data analysis appropriate for study design and type of data) | 2 (Beyond descriptive analysis) | 3 (Patient/health care outcome) | 14 |
| Guasch 2010 | Monitoring skill acquisition in obstetric epidural puncture at a university hospital using the cumulative sum method | 1 (Single group cross-sectional or single group posttest only) | 0.5 (1 institution) | 1.5 (more than 75%) | 1 (Assessment by study participant) | 0 (Not reported) | 1 (Reported) | 0 (Not reported) | 1 (Data analysis appropriate for study design and type of data) | 1 (Descriptive analysis only) | 3 (Patient/health care outcome) | 10 |
| Guerra-Wallace 2010 | "Just-in-time" lumbar puncture simulation training for residents in the pediatric emergency department | 3 (Randomized controlled trial) | 0.5 (1 institution) | 1.5 (more than 75%) | 3 (Objective measurement) | 0 (Not reported) | 0 (Not reported) | 0 (Not reported) | 1 (Data analysis appropriate for study design and type of data) | 2 (Beyond descriptive analysis) | 3 (Patient/health care outcome) | 14 |
| Hale 2021 | Cohort study of hospitalists' procedural skills: Baseline competence and durability after simulation-based training | 1.5 (Single group pretest and posttest) | 0.5 (1 institution) | 1.5 (more than 75%) | 3 (Objective measurement) | 1 (Reported) | 1 (Reported) | 0 (Not reported) | 1 (Data analysis appropriate for study design and type of data) | 2 (Beyond descriptive analysis) | 1.5 (Knowledge, skills) | 13 |
| Henriksen 2017 | Assessment of Residents Readiness to Perform Lumbar Puncture: A Validation Study | 1 (Single group cross-sectional or single group posttest only) | 0.5 (1 institution) | 1.5 (more than 75%) | 3 (Objective measurement) | 1 (Reported) | 1 (Reported) | 1 (Reported) | 1 (Data analysis appropriate for study design and type of data) | 2 (Beyond descriptive analysis) | 1.5 (Knowledge, skills) | 13,5 |
| Huang 2023 | A mixed-reality stimulator for lumbar puncture training: a pilot study | 1.5 (Single group pretest and posttest) | 0.5 (1 institution) | 1.5 (more than 75%) | 1 (Assessment by study participant) | 0 (Not reported) | 0 (Not reported) | 0 (Not reported) | 1 (Data analysis appropriate for study design and type of data) | 2 (Beyond descriptive analysis) | 1 (Satisfaction, attitudes, perceptions, opinions, general facts) | 8,5 |
| Iyer 2013 | Assessing the validity evidence of an objective structured assessment tool of technical skills for neonatal lumbar punctures | 1 (Single group cross-sectional or single group posttest only) | 0.5 (1 institution) | 1.5 (more than 75%) | 3 (Objective measurement) | 1 (Reported) | 1 (Reported) | 1 (Reported) | 1 (Data analysis appropriate for study design and type of data) | 2 (Beyond descriptive analysis) | 1.5 (Knowledge, skills) | 13,5 |
| Kallidaikurichi-Srinivasan 2018 | Proficiency-based progression training: an 'end to end' model for decreasing error applied to achievement of effective epidural analgesia during labour: a randomised control study | 3 (Randomized controlled trial) | 0.5 (1 institution) | 1.5 (more than 75%) | 3 (Objective measurement) | 1 (Reported) | 1 (Reported) | 1 (Reported) | 1 (Data analysis appropriate for study design and type of data) | 2 (Beyond descriptive analysis) | 3 (Patient/health care outcome) | 17 |
| Katz 2017 | Teaching procedural skills to medical students: A pilot procedural skills lab | 2 (Nonrandomized, 2 group) | 0.5 (1 institution) | 1 (50%-74%) | 1 (Assessment by study participant) | 0 (Not reported) | 0 (Not reported) | 0 (Not reported) | 1 (Data analysis appropriate for study design and type of data) | 2 (Beyond descriptive analysis) | 1 (Satisfaction, attitudes, perceptions, opinions, general facts) | 8,5 |
| Kessler 2013 | Interns' success with clinical procedures in infants after simulation training | 3 (Randomized controlled trial) | 1,5 (more than 2 institutions) | 1.5 (more than 75%) | 1 (Assessment by study participant) | 0 (Not reported) | 1 (Reported) | 1 (Reported) | 1 (Data analysis appropriate for study design and type of data) | 2 (Beyond descriptive analysis) | 3 (Patient/health care outcome) | 15 |
| Kessler 2011 | A randomized trial of simulation-based deliberate practice for infant lumbar puncture skills | 3 (Randomized controlled trial) | 0.5 (1 institution) | 1 (50%-74%) | 1 (Assessment by study participant) | 1 (Reported) | 1 (Reported) | 0 (Not reported) | 1 (Data analysis appropriate for study design and type of data) | 2 (Beyond descriptive analysis) | 3 (Patient/health care outcome) | 13,5 |
| Kessler 2017 | Screening residents for infant lumbar puncture readiness with just-in-time simulation-based assessments | 2 (Nonrandomized, 2 group) | 1,5 (more than 2 institutions) | 0.5 (less than 50% or not reported) | 1 (Assessment by study participant) | 0 (Not reported) | 0 (Not reported) | 0 (Not reported) | 1 (Data analysis appropriate for study design and type of data) | 2 (Beyond descriptive analysis) | 3 (Patient/health care outcome) | 11 |
| Kilbane 2010 | Pediatric residents' ability to perform a lumbar puncture: Evaluation of an educational intervention | 2 (Nonrandomized, 2 group) | 0.5 (1 institution) | 1.5 (more than 75%) | 3 (Objective measurement) | 1 (Reported) | 1 (Reported) | 0 (Not reported) | 1 (Data analysis appropriate for study design and type of data) | 2 (Beyond descriptive analysis) | 3 (Patient/health care outcome) | 15 |
| Konrad 1998 | Learning manual skills in anesthesiology: Is there a recommended number of cases for anesthetic procedures? | 1 (Single group cross-sectional or single group posttest only) | 0.5 (1 institution) | 1.5 (more than 75%) | 1 (Assessment by study participant) | 0 (Not reported) | 0 (Not reported) | 0 (Not reported) | 1 (Data analysis appropriate for study design and type of data) | 2 (Beyond descriptive analysis) | 3 (Patient/health care outcome) | 10 |
| Kopacz 1996 | The regional anesthesia learning curve: What is the minimum number of epidural and spinal blocks to reach consistency? | 1 (Single group cross-sectional or single group posttest only) | 0.5 (1 institution) | 1.5 (more than 75%) | 3 (Objective measurement) | 0 (Not reported) | 0 (Not reported) | 0 (Not reported) | 1 (Data analysis appropriate for study design and type of data) | 2 (Beyond descriptive analysis) | 3 (Patient/health care outcome) | 12 |
| Krause 2016 | Utilizing a multimodal approach in teaching medical students ultrasound-guided procedures | 1.5 (Single group pretest and posttest) | 1,5 (more than 2 institutions) | 0.5 (less than 50% or not reported) | 1 (Assessment by study participant) | 0 (Not reported) | 0 (Not reported) | 0 (Not reported) | 1 (Data analysis appropriate for study design and type of data) | 2 (Beyond descriptive analysis) | 1 (Satisfaction, attitudes, perceptions, opinions, general facts) | 8,5 |
| Kulcsár 2013 | Preliminary evaluation of a virtual reality-based simulator for learning spinal anesthesia | 3 (Randomized controlled trial) | 0.5 (1 institution) | 0.5 (less than 50% or not reported) | 3 (Objective measurement) | 1 (Reported) | 0 (Not reported) | 0 (Not reported) | 1 (Data analysis appropriate for study design and type of data) | 2 (Beyond descriptive analysis) | 3 (Patient/health care outcome) | 14 |
| Lean 2017 | End-task versus in-task feedback to increase procedural learning retention during spinal anaesthesia training of novices | 3 (Randomized controlled trial) | 0.5 (1 institution) | 1.5 (more than 75%) | 3 (Objective measurement) | 1 (Reported) | 1 (Reported) | 0 (Not reported) | 1 (Data analysis appropriate for study design and type of data) | 2 (Beyond descriptive analysis) | 1.5 (Knowledge, skills) | 14,5 |
| Lenchus 2011 | A blended approach to invasive bedside procedural instruction | 1.5 (Single group pretest and posttest) | 0.5 (1 institution) | 1.5 (more than 75%) | 3 (Objective measurement) | 0 (Not reported) | 1 (Reported) | 0 (Not reported) | 1 (Data analysis appropriate for study design and type of data) | 2 (Beyond descriptive analysis) | 3 (Patient/health care outcome) | 13,5 |
| Lenhard 2008 | An intervention to improve procedure education for internal medicine residents | 1.5 (Single group pretest and posttest) | 0.5 (1 institution) | 1.5 (more than 75%) | 1 (Assessment by study participant) | 0 (Not reported) | 0 (Not reported) | 0 (Not reported) | 1 (Data analysis appropriate for study design and type of data) | 2 (Beyond descriptive analysis) | 1 (Satisfaction, attitudes, perceptions, opinions, general facts) | 8,5 |
| Lew 2020 | Determining competence in performing obstetric combined spinal-epidural procedures in junior anesthesiology residents: results from a cumulative sum analysis | 1 (Single group cross-sectional or single group posttest only) | 0.5 (1 institution) | 1.5 (more than 75%) | 1 (Assessment by study participant) | 0 (Not reported) | 1 (Reported) | 0 (Not reported) | 1 (Data analysis appropriate for study design and type of data) | 1 (Descriptive analysis only) | 3 (Patient/health care outcome) | 10 |
| Lilamand 2023 | Lumbar puncture training with healthcare simulation improves self-confidence and practical skills of French medical residents in geriatrics | 1.5 (Single group pretest and posttest) | 1,5 (more than 2 institutions) | 0.5 (less than 50% or not reported) | 1 (Assessment by study participant) | 0 (Not reported) | 0 (Not reported) | 0 (Not reported) | 1 (Data analysis appropriate for study design and type of data) | 2 (Beyond descriptive analysis) | 3 (Patient/health care outcome) | 10,5 |
| Lim 2016 | Low-Fidelity Haptic Simulation Versus Mental Imagery Training for Epidural Anesthesia Technical Achievement in Novice Anesthesiology Residents: A Randomized Comparative Study | 3 (Randomized controlled trial) | 0.5 (1 institution) | 1.5 (more than 75%) | 3 (Objective measurement) | 0 (Not reported) | 1 (Reported) | 1 (Reported) | 1 (Data analysis appropriate for study design and type of data) | 2 (Beyond descriptive analysis) | 1.5 (Knowledge, skills) | 14,5 |
| Lydon 2019 | Can simulation-based education and precision teaching improve paediatric trainees' behavioural fluency in performing lumbar puncture? A pilot study | 2 (Nonrandomized, 2 group) | 0.5 (1 institution) | 1.5 (more than 75%) | 3 (Objective measurement) | 0 (Not reported) | 0 (Not reported) | 1 (Reported) | 1 (Data analysis appropriate for study design and type of data) | 2 (Beyond descriptive analysis) | 3 (Patient/health care outcome) | 14 |
| McMillan 2016 | Lumbar puncture simulation in pediatric residency training: Improving procedural competence and decreasing anxiety | 1.5 (Single group pretest and posttest) | 0.5 (1 institution) | 1.5 (more than 75%) | 3 (Objective measurement) | 0 (Not reported) | 1 (Reported) | 0 (Not reported) | 1 (Data analysis appropriate for study design and type of data) | 2 (Beyond descriptive analysis) | 1.5 (Knowledge, skills) | 12 |
| Meerkov 2017 | A simulation-based procedural curriculum for pediatric interns improves self-perceived competence | 1.5 (Single group pretest and posttest) | 0.5 (1 institution) | 0.5 (less than 50% or not reported) | 1 (Assessment by study participant) | 0 (Not reported) | 0 (Not reported) | 0 (Not reported) | 0 (Data analysis inappropriate for study design or type of data) | 2 (Beyond descriptive analysis) | 1 (Satisfaction, attitudes, perceptions, opinions, general facts) | 6,5 |
| Melamed 2017 | Sim one, teach one-senior resident-led pediatric intern procedural training | 2 (Nonrandomized, 2 group) | 0.5 (1 institution) | 1.5 (more than 75%) | 3 (Objective measurement) | 0 (Not reported) | 0 (Not reported) | 0 (Not reported) | 1 (Data analysis appropriate for study design and type of data) | 2 (Beyond descriptive analysis) | 1.5 (Knowledge, skills) | 11,5 |
| Mohamed 2021 | Proficiency-based progression training: implementing a novel approach to training for epidural analgesia in labour | 1 (Single group cross-sectional or single group posttest only) | 0.5 (1 institution) | 1.5 (more than 75%) | 3 (Objective measurement) | 1 (Reported) | 1 (Reported) | 1 (Reported) | 1 (Data analysis appropriate for study design and type of data) | 2 (Beyond descriptive analysis) | 3 (Patient/health care outcome) | 15 |
| Mourad 2012 | A randomized controlled trial of the impact of a teaching procedure service on the training of internal medicine residents. | 3 (Randomized controlled trial) | 0.5 (1 institution) | 1.5 (more than 75%) | 1 (Assessment by study participant) | 0 (Not reported) | 0 (Not reported) | 1 (Reported) | 1 (Data analysis appropriate for study design and type of data) | 2 (Beyond descriptive analysis) | 2 (Behaviors) | 12 |
| Mousavi 2023 | Effects of a Novel Blended Virtual Reality and Clinical Learning Environment on the Learning Transfer of Anesthesiology Residents | 3 (Randomized controlled trial) | 0.5 (1 institution) | 1.5 (more than 75%) | 3 (Objective measurement) | 1 (Reported) | 1 (Reported) | 0 (Not reported) | 1 (Data analysis appropriate for study design and type of data) | 2 (Beyond descriptive analysis) | 3 (Patient/health care outcome) | 16 |
| Munoz-Leija 2024 | Development and Evaluation of An In-House Lumbar Puncture Simulator for First-Year Resident Lumbar Puncture Procedure Learning. | 3 (Randomized controlled trial) | 0.5 (1 institution) | 1.5 (more than 75%) | 3 (Objective measurement) | 0 (Not reported) | 0 (Not reported) | 0 (Not reported) | 1 (Data analysis appropriate for study design and type of data) | 2 (Beyond descriptive analysis) | 3 (Patient/health care outcome) | 14 |
| Naik 2003 | Cusum analysis is a useful tool to assess resident proficiency at insertion of labour epidurals | 1 (Single group cross-sectional or single group posttest only) | 0.5 (1 institution) | 1.5 (more than 75%) | 1 (Assessment by study participant) | 0 (Not reported) | 1 (Reported) | 0 (Not reported) | 1 (Data analysis appropriate for study design and type of data) | 1 (Descriptive analysis only) | 3 (Patient/health care outcome) | 10 |
| Ocel 2006 | Formal procedural skills training using a fresh frozen cadaver model: A pilot study | 1.5 (Single group pretest and posttest) | 0.5 (1 institution) | 1.5 (more than 75%) | 1 (Assessment by study participant) | 0 (Not reported) | 0 (Not reported) | 0 (Not reported) | 1 (Data analysis appropriate for study design and type of data) | 1 (Descriptive analysis only) | 1 (Satisfaction, attitudes, perceptions, opinions, general facts) | 7,5 |
| Oxentenko 2003 | A multidimensional workshop using human cadavers to teach bedside procedures | 1.5 (Single group pretest and posttest) | 0.5 (1 institution) | 1.5 (more than 75%) | 1 (Assessment by study participant) | 0 (Not reported) | 0 (Not reported) | 0 (Not reported) | 1 (Data analysis appropriate for study design and type of data) | 2 (Beyond descriptive analysis) | 1.5 (Knowledge, skills) | 9 |
| Parlar-Chun 2019 | The effect of lumbar puncture simulations on pediatric residents | 1.5 (Single group pretest and posttest) | 0.5 (1 institution) | 0.5 (less than 50% or not reported) | 3 (Objective measurement) | 0 (Not reported) | 0 (Not reported) | 0 (Not reported) | 1 (Data analysis appropriate for study design and type of data) | 2 (Beyond descriptive analysis) | 3 (Patient/health care outcome) | 11,5 |
| Patel 2008 | Training effect of skills courses on confidence of junior doctors performing clinical procedures | 1.5 (Single group pretest and posttest) | 0.5 (1 institution) | 1.5 (more than 75%) | 1 (Assessment by study participant) | 0 (Not reported) | 0 (Not reported) | 1 (Reported) | 1 (Data analysis appropriate for study design and type of data) | 2 (Beyond descriptive analysis) | 1 (Satisfaction, attitudes, perceptions, opinions, general facts) | 9,5 |
| Reinhardt 2012 | Intern self-assessment and prediction of lumbar puncture success | 1 (Single group cross-sectional or single group posttest only) | 1,5 (more than 2 institutions) | 0.5 (less than 50% or not reported) | 3 (Objective measurement) | 0 (Not reported) | 1 (Reported) | 0 (Not reported) | 1 (Data analysis appropriate for study design and type of data) | 2 (Beyond descriptive analysis) | 3 (Patient/health care outcome) | 13 |
| Restrepo 2015 | Ability of pediatric emergency medicine physicians to identify anatomic landmarks with the assistance of ultrasound prior to lumbar puncture in a simulated obese model | 1.5 (Single group pretest and posttest) | 0.5 (1 institution) | 1.5 (more than 75%) | 1 (Assessment by study participant) | 1 (Reported) | 0 (Not reported) | 0 (Not reported) | 1 (Data analysis appropriate for study design and type of data) | 2 (Beyond descriptive analysis) | 1 (Satisfaction, attitudes, perceptions, opinions, general facts) | 9,5 |
| Roehr 2021 | The Feasibility of Virtual Reality and Student-Led Simulation Training as Methods of Lumbar Puncture Instruction | 3 (Randomized controlled trial) | 0.5 (1 institution) | 1.5 (more than 75%) | 3 (Objective measurement) | 1 (Reported) | 1 (Reported) | 0 (Not reported) | 1 (Data analysis appropriate for study design and type of data) | 2 (Beyond descriptive analysis) | 1.5 (Knowledge, skills) | 14,5 |
| Sattler 2020 | Simulation-Based Medical Education Improves Procedural Confidence in Core Invasive Procedures for Military Internal Medicine Residents. | 1.5 (Single group pretest and posttest) | 0.5 (1 institution) | 1 (50%-74%) | 1 (Assessment by study participant) | 0 (Not reported) | 0 (Not reported) | 0 (Not reported) | 1 (Data analysis appropriate for study design and type of data) | 2 (Beyond descriptive analysis) | 1 (Satisfaction, attitudes, perceptions, opinions, general facts) | 8 |
| Shaikh 2021 | Feasibility of ultrasound-assisted lumbar punctures performed by pediatric oncologists at the point of care. | 1 (Single group cross-sectional or single group posttest only) | 0.5 (1 institution) | 1.5 (more than 75%) | 3 (Objective measurement) | 0 (Not reported) | 0 (Not reported) | 0 (Not reported) | 1 (Data analysis appropriate for study design and type of data) | 1 (Descriptive analysis only) | 3 (Patient/health care outcome) | 11 |
| Shammari 2018 | Evaluation of effectiveness of a paediatric simulation course in procedural skills for paediatric residents — A pilot study | 1.5 (Single group pretest and posttest) | 0.5 (1 institution) | 1.5 (more than 75%) | 3 (Objective measurement) | 1 (Reported) | 1 (Reported) | 0 (Not reported) | 1 (Data analysis appropriate for study design and type of data) | 2 (Beyond descriptive analysis) | 1.5 (Knowledge, skills) | 13 |
| Stolarek 2007 | Procedural and examination skills of first-year house surgeons: A comparison of a simulation workshop versus 6 months of clinical ward experience alone | 2 (Nonrandomized, 2 group) | 0.5 (1 institution) | 0.5 (less than 50% or not reported) | 1 (Assessment by study participant) | 0 (Not reported) | 0 (Not reported) | 0 (Not reported) | 1 (Data analysis appropriate for study design and type of data) | 2 (Beyond descriptive analysis) | 1 (Satisfaction, attitudes, perceptions, opinions, general facts) | 8 |
| Sun 2018 | Evaluation of Problem- and Simulator-Based Learning in Lumbar Puncture in Adult Neurology Residency Training | 3 (Randomized controlled trial) | 0.5 (1 institution) | 1.5 (more than 75%) | 3 (Objective measurement) | 0 (Not reported) | 0 (Not reported) | 0 (Not reported) | 1 (Data analysis appropriate for study design and type of data) | 2 (Beyond descriptive analysis) | 3 (Patient/health care outcome) | 14 |
| Toy 2017 | Using Learner-Centered, Simulation-Based Training to Improve Medical Students' Procedural Skills. | 1.5 (Single group pretest and posttest) | 0.5 (1 institution) | 1.5 (more than 75%) | 3 (Objective measurement) | 1 (Reported) | 0 (Not reported) | 0 (Not reported) | 1 (Data analysis appropriate for study design and type of data) | 2 (Beyond descriptive analysis) | 1.5 (Knowledge, skills) | 12 |
| Udani 2014 | Simulation-Based Mastery Learning with Deliberate Practice Improves Clinical Performance in Spinal Anesthesia | 3 (Randomized controlled trial) | 0.5 (1 institution) | 1.5 (more than 75%) | 3 (Objective measurement) | 1 (Reported) | 1 (Reported) | 0 (Not reported) | 1 (Data analysis appropriate for study design and type of data) | 2 (Beyond descriptive analysis) | 3 (Patient/health care outcome) | 16 |
| Valentine 2019 | Outcomes of a boot camp for incoming neurology residents | 1.5 (Single group pretest and posttest) | 0.5 (1 institution) | 0.5 (less than 50% or not reported) | 1 (Assessment by study participant) | 0 (Not reported) | 0 (Not reported) | 0 (Not reported) | 1 (Data analysis appropriate for study design and type of data) | 1 (Descriptive analysis only) | 1 (Satisfaction, attitudes, perceptions, opinions, general facts) | 6,5 |
| Vassallo 2015 | Lumbar puncture training using simulation-based educational strategies. Experience in a clinical pediatric residency | 1.5 (Single group pretest and posttest) | 0.5 (1 institution) | 1.5 (more than 75%) | 3 (Objective measurement) | 0 (Not reported) | 0 (Not reported) | 0 (Not reported) | 1 (Data analysis appropriate for study design and type of data) | 1 (Descriptive analysis only) | 1.5 (Knowledge, skills) | 10 |
| Vilasagar 2013 | Does a pediatric lumbar puncture program lead to sustained improvement in resident skill? | 2 (Nonrandomized, 2 group) | 1 (2 institutions) | 1.5 (more than 75%) | 1 (Assessment by study participant) | 0 (Not reported) | 1 (Reported) | 0 (Not reported) | 1 (Data analysis appropriate for study design and type of data) | 2 (Beyond descriptive analysis) | 3 (Patient/health care outcome) | 12,5 |
| Von Cranach 2019 | Medical students' attitudes toward lumbar puncture—And how to change | 1.5 (Single group pretest and posttest) | 0.5 (1 institution) | 1 (50%-74%) | 1 (Assessment by study participant) | 0 (Not reported) | 1 (Reported) | 0 (Not reported) | 1 (Data analysis appropriate for study design and type of data) | 2 (Beyond descriptive analysis) | 1 (Satisfaction, attitudes, perceptions, opinions, general facts) | 9 |
| Vusse 2020 | Procedure Training Workshop for Internal Medicine Residents that Emphasizes Procedural Ultrasound: Logistics and Teaching Materials | 1.5 (Single group pretest and posttest) | 0.5 (1 institution) | 0.5 (less than 50% or not reported) | 1 (Assessment by study participant) | 0 (Not reported) | 0 (Not reported) | 0 (Not reported) | 1 (Data analysis appropriate for study design and type of data) | 2 (Beyond descriptive analysis) | 1 (Satisfaction, attitudes, perceptions, opinions, general facts) | 7,5 |
| Wang 2024 | Continuing medical education for attending physicians in anesthesia: Feasibility of an innovative blended learning approach | 3 (Randomized controlled trial) | 0.5 (1 institution) | 1.5 (more than 75%) | 3 (Objective measurement) | 0 (Not reported) | 0 (Not reported) | 0 (Not reported) | 1 (Data analysis appropriate for study design and type of data) | 2 (Beyond descriptive analysis) | 1.5 (Knowledge, skills) | 12,5 |
| Wayne 2014 | Progress toward improving medical school graduates' skills via a "boot camp" curriculum | 2 (Nonrandomized, 2 group) | 0.5 (1 institution) | 1.5 (more than 75%) | 3 (Objective measurement) | 1 (Reported) | 1 (Reported) | 1 (Reported) | 1 (Data analysis appropriate for study design and type of data) | 2 (Beyond descriptive analysis) | 1.5 (Knowledge, skills) | 14,5 |
| Weil 2017 | Learning curves for three specific procedures by anesthesiology residents using the learning curve cumulative sum (LC-CUSUM) test | 1 (Single group cross-sectional or single group posttest only) | 0.5 (1 institution) | 1.5 (more than 75%) | 1 (Assessment by study participant) | 0 (Not reported) | 1 (Reported) | 0 (Not reported) | 1 (Data analysis appropriate for study design and type of data) | 1 (Descriptive analysis only) | 3 (Patient/health care outcome) | 10 |
| Westwood 2012 | Lumbar puncture simulation training improves medical student knowledge and confidence | 1.5 (Single group pretest and posttest) | 0.5 (1 institution) | 1.5 (more than 75%) | 1 (Assessment by study participant) | 0 (Not reported) | 0 (Not reported) | 0 (Not reported) | 1 (Data analysis appropriate for study design and type of data) | 1 (Descriptive analysis only) | 1 (Satisfaction, attitudes, perceptions, opinions, general facts) | 7,5 |
| White 2012 | Transfer of simulated lumbar puncture training to the clinical setting | 1.5 (Single group pretest and posttest) | 0.5 (1 institution) | 1.5 (more than 75%) | 3 (Objective measurement) | 0 (Not reported) | 1 (Reported) | 0 (Not reported) | 1 (Data analysis appropriate for study design and type of data) | 2 (Beyond descriptive analysis) | 3 (Patient/health care outcome) | 13,5 |
| Wiggins 2018 | Using evidence-based best practices of simulation, checklists, deliberate practice, and debriefing to develop and improve a regional anesthesia training course | 1.5 (Single group pretest and posttest) | 0.5 (1 institution) | 1.5 (more than 75%) | 1 (Assessment by study participant) | 0 (Not reported) | 1 (Reported) | 0 (Not reported) | 1 (Data analysis appropriate for study design and type of data) | 1 (Descriptive analysis only) | 1 (Satisfaction, attitudes, perceptions, opinions, general facts) | 8,5 |
| Williams 2018 | Simulation-based mastery learning improves lumbar puncture but not paracentesis performance | 3 (Randomized controlled trial) | 0.5 (1 institution) | 1.5 (more than 75%) | 3 (Objective measurement) | 0 (Not reported) | 1 (Reported) | 0 (Not reported) | 1 (Data analysis appropriate for study design and type of data) | 2 (Beyond descriptive analysis) | 3 (Patient/health care outcome) | 15 |
| Wong 2018 | Developing a child neurology training program in Cambodia: A pilot study | 1.5 (Single group pretest and posttest) | 0.5 (1 institution) | 1.5 (more than 75%) | 1 (Assessment by study participant) | 0 (Not reported) | 0 (Not reported) | 0 (Not reported) | 1 (Data analysis appropriate for study design and type of data) | 1 (Descriptive analysis only) | 1 (Satisfaction, attitudes, perceptions, opinions, general facts) | 7,5 |
| Xie 2023 | Using a novel virtual-reality simulator to assess performance in lumbar puncture: a validation study | 1 (Single group cross-sectional or single group posttest only) | 0.5 (1 institution) | 1.5 (more than 75%) | 3 (Objective measurement) | 1 (Reported) | 1 (Reported) | 1 (Reported) | 1 (Data analysis appropriate for study design and type of data) | 2 (Beyond descriptive analysis) | 1.5 (Knowledge, skills) | 13,5 |
| Yanta 2020 | The Use of Hybrid Lumbar Puncture Simulation to Teach Entrustable Professional Activities During a Medical Student Neurology Clerkship. | 1.5 (Single group pretest and posttest) | 0.5 (1 institution) | 1.5 (more than 75%) | 1 (Assessment by study participant) | 0 (Not reported) | 0 (Not reported) | 0 (Not reported) | 1 (Data analysis appropriate for study design and type of data) | 2 (Beyond descriptive analysis) | 1 (Satisfaction, attitudes, perceptions, opinions, general facts) | 8,5 |
| Yee 2022 | Procedural Curriculum to Verify Intern Competence Prior to Patient Care. | 1.5 (Single group pretest and posttest) | 0.5 (1 institution) | 1.5 (more than 75%) | 3 (Objective measurement) | 0 (Not reported) | 1 (Reported) | 0 (Not reported) | 1 (Data analysis appropriate for study design and type of data) | 2 (Beyond descriptive analysis) | 1.5 (Knowledge, skills) | 12 |
| Yeo 2015 | Examination of learning trajectories for simulated lumbar puncture training using hand motion analysis | 1.5 (Single group pretest and posttest) | 0.5 (1 institution) | 1.5 (more than 75%) | 3 (Objective measurement) | 0 (Not reported) | 1 (Reported) | 0 (Not reported) | 1 (Data analysis appropriate for study design and type of data) | 2 (Beyond descriptive analysis) | 1.5 (Knowledge, skills) | 12 |
