# Supplementary material for: Training and assessment of skills in neuraxial space access: a scoping review of educational approaches to lumbar puncture, epidural anaesthesia, and spinal anaesthesia
Source: Br J Anaesth. 2025 Jul 7;135(4):1026–37. doi: 10.1016/j.bja.2025.06.008 (PMC12674033; doi:10.1016/j.bja.2025.06.008)
Supplement: Multimedia component 7 [file mmc7.docx]

*Appendix 7 - MERSQI item scores of included 99 studies*

| **Study design** (points) | **n (%)** |
| --- | --- |
| Single group cross-sectional or post-test only (1) | 24 (24) |
| Single group pretest and post-test (1.5) | 39 (39) |
| Nonrandomized controlled trial (2) | 12 (12) |
| Randomized controlled trial (3) | 24 (24) |
| **Sampling: institutions** (points) | |
| 1 institution (0.5) | 88 (89) |
| 2 institutions (1) | 2 (2) |
| 3 or more institutions (1.5) | 9 (9) |
| **Sampling: response rate** (points) |  |
| <50% or not reported (0.5) | 18 (18) |
| 50%–74% (1) | 9 (9) |
| ≥75% (1.5) | 72 (73) |
| **Type of data** (points) | |
| Assessment by study participant (1) | 41 (41) |
| Objective (3) | 58 (59) |
| **Validity evidence for evaluation instrument** (points) | |
| Internal structure (1) | 31 (31) |
| Content (1) | 48 (49) |
| Relationships to other variables (1) | 26 (26) |
| **Data analysis** (points) | |
| Appropriate for study design and type of data (1) | 97 (98) |
| Complexity: descriptive analysis only (1) | 16 (16) |
| Complexity: beyond descriptive analysis (2) | 83 (84) |
| **Outcome** (points) | |
| Satisfaction, attitudes, perceptions, opinions, general facts (1) | 28 (28) |
| Knowledge, skills (1.5) | 32 (32) |
| Behaviors (2) | 4 (4) |
| Patient/health care outcome (3) | 35 (35) |
| **Total score** | |
| Mean 11.6 points, range from 6.5-18.0 points | |
